# A phylogenetic mosaic plastid proteome and unusual plastid-targeting signals in the green-colored dinoflagellate *Lepidodinium chlorophorum*

**DOI:** 10.1186/1471-2148-10-191

**Published:** 2010-06-21

**Authors:** Marianne A Minge, Kamran Shalchian-Tabrizi, Ole K Tørresen, Kiyotaka Takishita, Ian Probert, Yuji Inagaki, Dag Klaveness, Kjetill S Jakobsen

**Affiliations:** 1 Centre for Ecological and Evolutionary Synthesis (CEES), Department of Biology, University of Oslo, N-0316 Oslo, Norway; 2 Microbial Evolutionary Research Group (MERG), Department of Biology, University of Oslo, N-0316 Oslo, Norway; 3 Japan Agency for Marine-Earth Science and Technology (JAMSTEC), Yokosuka, Kanagawa, 237-0061, Japan; 4 Roscoff Culture Collection (RCC), Station Biologique de Roscoff, Place Georges Teissier, 29682 Roscoff, France; 5 Center for Computational Sciences, Institute for Biological Sciences, University of Tsukuba, Tsukuba Ibaraki, 305-8577, Japan

## Abstract

**Background:**

Plastid replacements through secondary endosymbioses include massive transfer of genes from the endosymbiont to the host nucleus and require a new targeting system to enable transport of the plastid-targeted proteins across 3-4 plastid membranes. The dinoflagellates are the only eukaryotic lineage that has been shown to have undergone several plastid replacement events, and this group is thus highly relevant for studying the processes involved in plastid evolution. In this study, we analyzed the phylogenetic origin and N-terminal extensions of plastid-targeted proteins from *Lepidodinium chlorophorum*, a member of the only dinoflagellate genus that harbors a green secondary plastid rather than the red algal-derived, peridinin-containing plastid usually found in photosynthetic dinoflagellates.

**Results:**

We sequenced 4,746 randomly picked clones from a *L. chlorophorum *cDNA library. 22 of the assembled genes were identified as genes encoding proteins functioning in plastids. Some of these were of green algal origin. This confirms that genes have been transferred from the plastid to the host nucleus of *L. chlorophorum *and indicates that the plastid is fully integrated as an organelle in the host. Other nuclear-encoded plastid-targeted protein genes, however, are clearly not of green algal origin, but have been derived from a number of different algal groups, including dinoflagellates, streptophytes, heterokonts, and red algae. The characteristics of N-terminal plastid-targeting peptides of all of these genes are substantially different from those found in peridinin-containing dinoflagellates and green algae.

**Conclusions:**

*L. chlorophorum *expresses plastid-targeted proteins with a range of different origins, which probably arose through endosymbiotic gene transfer (EGT) and horizontal gene transfer (HGT). The N-terminal extension of the genes is different from the extensions found in green alga and other dinoflagellates (peridinin- and haptophyte plastids). These modifications have likely enabled the mosaic proteome of *L. chlorophorum*.

## Background

Establishment of the plastid by endosymbiosis of a cyanobacterium was a key event in the evolutionary history of eukaryotes. An ancient primary endosymbiosis gave rise to photosynthethic organelles in members of the Viridiplantae, rhodophytes (red algae) and glaucophytes [1-5]. Such primary plastids (bound by two membranes) were subsequently spread to other protist lineages through a series of eukaryote-eukaryote secondary and tertiary endosymbiotic events, resulting in a large diversity of photosynthetic lineages [6,7].

Both red and green algae have been involved in secondary endosymbioses. The exact number of secondary endosymbiotic events involving red algae remains controversial [8-12], with theories ranging from a single uptake in the common ancestor of all algal lineages harboring secondary plastids derived from a red alga (the chromalveolate hypothesis) to serial independent uptakes of red algal plastids or transfer of red alga-derived plastids between algal groups [4,8,13,14]. Green plastids of secondary origin are known to be found in three distinct algal lineages: the chlorarachniophytes, the photosynthetic euglenids and in the dinoflagellate genus *Lepidodinium*. The chlorarachniophytes are marine phototrophic amoeboflagellates belonging to the lineage Cercozoa within the eukaryote 'supergroup' Rhizaria [15-17], while the euglenids are the only photosynthetic lineage in the 'supergroup' Excavata [18-20]. The dinoflagellate genus *Lepidodinium *contains a secondary chlorophyll *a *and *b *containing plastid derived from a green alga [21-24]. The distant evolutionary relationships between the chlorarachniophyte, euglenid and dinoflagellate host lineages indicate that their plastids originate from three distinct endoymbiotic events [7,18,25,26].

One of the main processes during plastid establishment is massive transfer of genes from the endosymbiont to the host nucleus, and the invention of protein trafficking machineries that involve protein synthesis in the host cytoplasm and direction of proteins to the plastids [27,28]. Consequently, the plastids themselves encode only a minor fraction of the genes necessary for proper plastid function. In phototrophic organisms harboring primary plastids, these proteins are targeted back to the plastid by a transit peptide that directs the proteins across the double membrane [29]. In algae with secondary and tertiary plastids, proteins need (i) to have the N-terminal bipartite presequences that code for a signal peptide that directs the protein to the host endomembrane system and a transit peptide to lead the protein to the plastid, and (ii) to be transported across 3-4 membrane layers resulting from the establishment of the organelle [5,30].

Intriguingly, several algal lineages, such as the red algae *Cyanidioschyzon merolae*, the land plant *Arabidopsis thaliana*, various chromalveolates and the chlorarachniophyte *Bigelowiella natans*, seem to have nucleus-encoded plastid-targeted protein genes originating from phylogenetically divergent algae [15,31-36]. The plastid proteomes of such organisms are therefore not acquired only from their current plastids, but may also include proteins originating from other sources.

Dinoflagellates are unique among eukaryotic lineages in having undergone several plastid replacement events [37-41], and are therefore a key source of insights into the process of transformation of an endosymbiotic alga into an organelle. The most canonical dinoflagellate plastid type contains chlorophyll *c *and the pigment peridinin and is likely the ancestral plastid of the group [9,42]. However, in several dinoflagellate lineages the peridinin-containing plastid has been lost, giving rise to a range of heterotrophic species, or has been replaced by plastids acquired from other algal groups (i.e. haptophytes, heterokonts, cryptomonads, and green algae). The level of plastid integration varies among dinoflagellates, ranging from fully integrated permanent plastid replacements such as the tertiary haptophyte-derived plastids found in *Karenia *and *Karlodinium *species [31] to less integrated plastids (i.e. permanent plastids that have not been degenerated) or temporary associations [39,41]. *Lepidodinium *is the only dinoflagellate genus that has replaced the red alga-derived peridinin-containing plastid by a green plastid [21,37], and thus represents a model for studying events occurring in the process of plastid replacement. In this context, an important issue is to understand how protein-targeting signals of pre-existing proteins from the original plastid have adapted to the green plastid environment. Furthermore, another key question concerns the evolutionary origins of plastid-targeted protein genes in the host genome in *Lepidodinium*. In fact, the plastid-targeted GAPDH gene in *L. chlorophorum *was recently shown to be of haptophyte origin [24], demonstrating that plastid-targeted genes in this species may originate from sources other than the original peridinin-containing plastid or the current green algal plastid.

We addressed these questions by investigating the nuclear-encoded plastid proteome of *L. chlorophorum*. We present 22 plastid-targeted genes identified in a cDNA library. The complete N-terminal extensions were obtained by 5'- RACE experiments of 20 of the 22 plastid-targeted genes. Phylogenetic analyses of the plastid-targeted protein genes reveal a mosaic plastid proteome in *L. chlorophorum*; many of the *L. chlorophorum *sequences showed apparent affinities to homologs from green algae, haptophytes, heterokonts, and peridinin-containing dinoflagellates. The N-terminal presequences (likely associated with plastid targeting) of *L. chlorophorum *differ from those of other dinoflagellates in having relatively low levels of serine and threonine residues, a high level of acidic residues and possibly an abnormally long hydrophilic region near the N-terminal end.

## Results

### Plastid-targeted proteins in *L. chlorophorum*

4,746 clones from the *L. chlorophorum *cDNA-library were sequenced from the 5'-end, and 22 plastid-targeted protein genes were identified (Table 1). These correspond to a wide range of plastid-associated processes, and include components of photosystem II (PsbO, PsbP, PsbR), the gamma subunit of ATP synthase, ferredoxin B, ferredoxin NADP reductase, the small subunit of ribulose-1,5-bisphosphate carboxylase/oxygenase (RuBisCO), RuBisCO activase, fructose-1,6-bisphosphatase, phosphoribulokinase and several isoforms of light harvesting proteins. In addition, three genes encoding enzymes involved in the non-mevalonate isopentyl disphosphate (isoprenoid biosynethesis) pathway were identified.

**Table 1:** Plastid-targeted genes detected in the *Lepidodinium chlorophorum *cDNA library

Protein	Origin^1)^	Support^2)^
Plastid lipid associated protein kinase	Green algae	85
PsbO	Green algae	53
PsbP	Green algae	53
PsbR	Green algae	100
RuBisCO activase	Green algae	99
Chlorophyll a/b binding protein (several isoforms)	Green algae	<50
1-deoxy-D-xylulose 5-phosphate reductoisomerase	Peridinin dinoflagellate	100
4-diphosphocytidyl-2C-methyl-D-erythritol kinase	Peridinin dinoflagellate	95
Ferredoxin NADP reductase	Peridinin dinoflagellate	95
RuBisCO small subunit	Streptophyte	83
Plastid-targeted GAPDH	Haptophyte	
Phosphoribulokinase	Heterokont	<50
Csp41	Heterokont	84
Chloroplast stability factor hcf 136	Red algae or derivate	98
Fructose 1,6 bisphosphatase	Red algae or derivate	80
Transketolase	Red algae or derivate	95
ATP synthase gamma	Red algae or derivate	<50
Chlorophyll a/c binding protein (several isoforms)	Red algae or derivate	<50
Sedoheptulose bisphosphatase	Unresolved phylogeny	
3,8 divinyl protochlorophyllide	Unresolved phylogeny	
DnaJ/Hsp40	Unresolved phylogeny	
Ferredoxin B	Unresolved phylogeny	

1) Phylogenetic origin of the genes as inferred by RAxML 2) Bootstrap support (100 pseudoreplicates and one heuristic search for each replicate) for the phylogenetic placement.

### The plastid-targeted genes have several evolutionary origins

**Figure 1:**
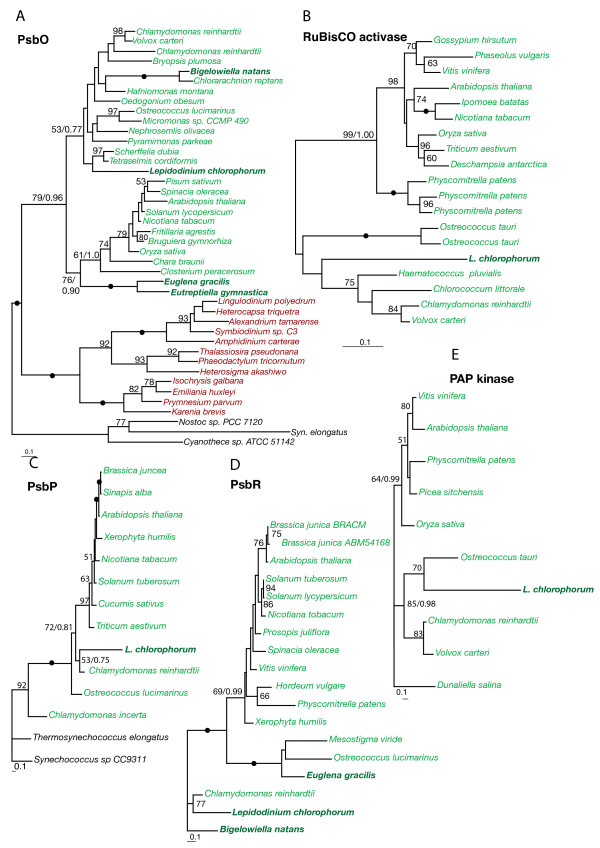
**Genes of green algal origin**. Maximum likelihood trees inferring a green-algal origin of *L. chlorophorum *of PsbO, RuBisCO activase, PsbP, PsbR and PAP protein kinase. The phylogenies were inferred using RAxML. Bootstrap values >50%are indicated on the branches. Green and red lineages are indicated by color. Secondary green algae are in bold. Filled dots indicate 100% bootstrap support. Bayesian posterior probability values are indicated for some of the most important splits.

**Figure 2:**
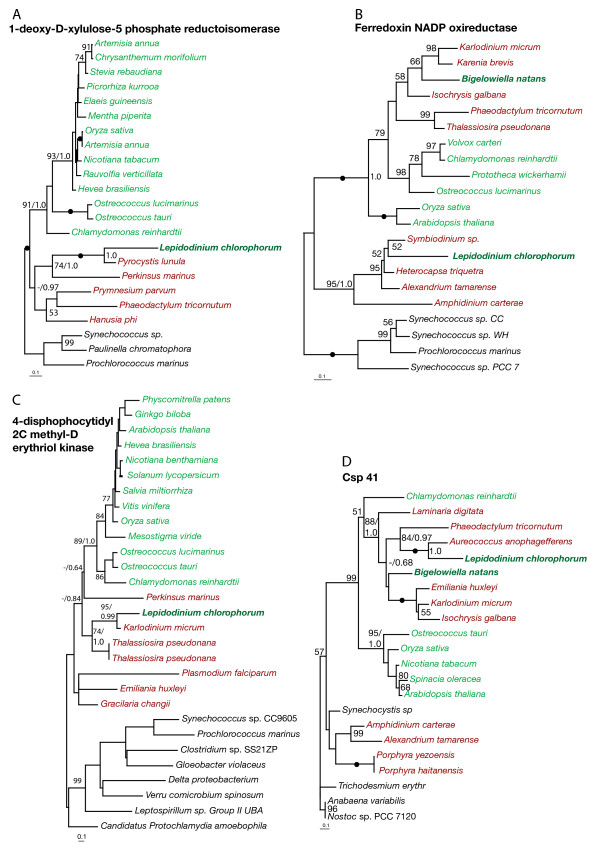
**Genes of peridinin- and heterokont origin**. Maximum likelihood trees of 1-deoxy-D-xylolose-5 phosphate reductoisomerase (Dvr1), ferredoxin NADP oxireductase, 4-disphophocytidyl-2C-methyl-D-erythriol kinase demonstrating that these genes are of peridinin dinoflagellate origin. Csp41 clusters with heterokonts. All trees were inferred using RAxML. Bootstrap values >50%are indicated on the branches. Green and red lineages are indicated by color and secondary green algae are in bold. Filled dots indicate 100% bootstrap support. Bayesian posterior probability values are indicated for some of the most important splits.

Phylogenies of all identified plastid-targeted proteins were inferred including sequences from members of as many major photosynthetic lineages as possible. Both Maximum likelihood, Bayesian analyses and distance methods were applied, resulting in qualitatively similar results. In five phylogenies - PsbO, PsbP, PsbR, RuBisCO activase and plastid lipid associated protein kinase (PAP kinase) - the *L. chlorophorum *homologs were unambiguously related to those of green algae and land plants (see Fig. 1 and Table 1). In the PsbO phylogeny (Fig. 1A), the *L. chlorophorum *sequence clustered within green algae and landplants, and was differentiated from red algal sequences with the maximum bootstrap support. PsbP, PsbR, RuBisCO activase and PAP kinase encode plastid proteins found exclusively (or almost exclusively) in green algae and land plants: (Fig. 1B-E) [43]. We also found genes possibly originating from the ancient peridinin plastid exemplified by the phylogenies of ferredoxin NADP reductase (Fig. 2B) and 1-deoxy-D-xylolose 5-phosphate reductoisomerase (Fig. 2A) where *L. chlorophorum *formed strongly supported clades with the peridinin-containing dinoflagellates (95% and 100% bootstrap supports). In the phylogeny of 4-disphophocytidyl 2C methyl-D erythriol kinase, *L. chlorophorum *branched with *K. veneficum *with high support (95%) (Fig. 2C and Table 1). In this tree, the two dinoflagellates grouped with heterokonts to the exclusion of haptophytes, even though the plastid in *K. veneficum *is of haptophyte origin indicating that both *K. veneficum *and *L. chlorophorum *either still utilize the homolog from the peridinin-containing plastids or that they both recruited this gene from the same or very closely related organisms.

**Figure 3:**
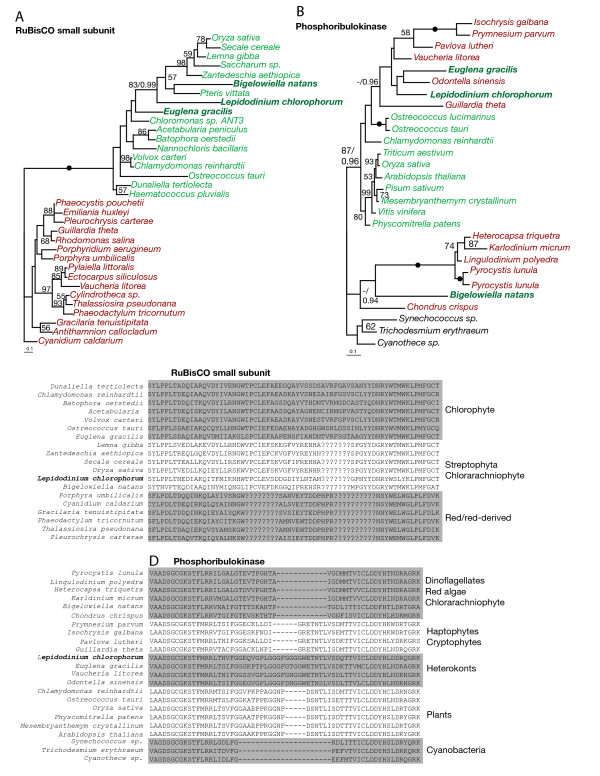
**Phylogenies supported by shared sequence characteristics**. **A: **Maximum likelihood trees inferring a heterokont origin of RuBisCO small subunit and phosphoribulokinase in *L. chlorophorum*. **B: **Alignment of the genes demonstrating shared sequence characteristics between *L. chlorophorum *and streptophytes and heterokonts, respectively. Filled dots indicate 100% bootstrap support. Bayesian posterior probability values are indicated for some of the most important splits.

Some of the other phylogenies strongly indicated that *L. chlorophorum *also harbors plastid-targeted protein genes that originate from sources other than green algae and the peridinin-containing dinoflagellate plastid. The phylogeny of RuBisCO small subunit (RbcS, Fig. 3A) shows that the *L. chlorophorum *homolog clusters with streptophytes with 81% bootstrap support; this origin is further supported by the presence of a streptophyte-specific insertion in the *L. chlorophorum *gene (Fig. 3C). In phylogenies of phosphoribulokinase (PRK, Fig. 3B) and csp41 (Fig. 2D), the *L. chlorophorum *homolog clustered with those of heterokonts. For csp41, this phylogenetic affinity was supported by 100% bootstrap support. The position of *L. chlorophorum *in the PRK phylogeny, however, is without significant statistical support, but the *L. chlorophorum *homolog bears a heterokont-specific insertion strongly indicating affinity to the homologs of heterokonts (Fig. 3D). A sequence identical to that of the previously reported plastid-targeted GAPDH gene was identified. This gene has previously been shown to be of haptophyte origin [24]. In four cases (fructose 1,6 bisphosphate, chloroplast stability factor hcf 136, transketolase and ATP synthase gamma, see Additional file 1 Figure S1 and Table 1), the *L. chlorophorum *homologs were excluded from those of green algae with high support, but their precise positions among red-algal derived homologs are unclear. In four of the phylogenies (Sedoheptulose bisphosphatase, 3,8 divinyl-protochlorophyllide, DnaJ and Ferredoxin B), the position of the *L. chlorphorum *gene-homolog remained unresolved due to insufficient taxon sampling and/or phylogenetic information (Additional file 2 Figure S2 and Table 1). Several isoforms of Chlorophyll a/b binding protein and Chlorophyll a/c binding proteins were also identified (Table 1).

### The N-terminal extensions (presequences) of *L. chlorophorum *are different from those found in other dinoflagellates and green algae

Based on the results of 5' RACE, the putative N-terminal presequences of 20 of the 22 plastid-targeted protein genes were identified. The dinoflagellate-specific spliced leader sequence [44] was detected in the 5'-end of all sequences, confirming that the entire 5'-end had been retrieved. High S-score values of neural networks implemented in the program SignalP 3.0 (SignalP-NN) [45] were obtained in all of the N-terminal extensions, indicating the presence of a signal peptide and therefore of a secretory pathway (Additional file 3 Figure S3, hydrophobicity plots). The transit peptide cleavage sites were also estimated using SignalP. Only three of the sequences (csp41, dvr1 and psbO) gave the definite cleavage sites of signal peptides when estimating with both SignalP-NN and hidden Markov models, also implemented in SignalP 3.0 (SignalP-HMM). In the remaining sequences, the two programs (SignalP-NN and SignalP-HMM) often inferred different positions of the cleavage site and the statistical significance of the estimated sites was rather low in several cases. This may be caused by an abnormally long hydrophilic region at the beginning of the N-terminal extension. Such a hydrophilic region has not previously been detected in dinoflagellates, but a similar feature has been reported from the early diverging alveolate *Perkinsus *[46]. After removing the hydrophilic region of N-terminal extensions of plastid-targeted proteins other than csp41, dvr1 and psbO, the probability for the cleavage sites was increased with SignalP (Fig. S3), but still remained relatively low. Although the precise cleavage positions of the 17 plastid-targeted proteins could not be conclusively determined in this study, it is likely that the signal peptides are cut off after targeting plastid ER, and we consider the putative transit peptide cleavage sites of these proteins as reasonable estimates for investigating the transit peptide characteristics. A logoplot based on the putative cleavage sites estimated using SignalP-HMM is shown in Additional file 4 Figure S4 - illustrating that no conserved sequence motif was found. Additional file 5 Figure S5 displays the distribution of amino acids in the estimated transit peptides and compares the distribution in transit peptides of various eukaryotic lineages [47]. The putative *L. chlorophorum *transit peptides have lower levels of serine and threonine residues compared to those of green algae, while the proportion of acidic residues is increased compared to most other transit peptides. Conserved cleavage site sequences and stop membrane anchors (STMAs) were not detected in the estimated transit peptides.

## Discussion

### *L. chlorophorum *contains a true plastid with a phylogenetically hybrid plastid proteome

The genus *Lepidodinium *is the only known dinoflagellate lineage that possesses a secondary plastid of green algal origin rather than the red algal derived peridinin-containing plastid found in most photosynthetic dinoflagellates [22-24,37]. The green *L. chlorophorum *plastid is maintained for years in laboratory cultures. This, together with the identified plastid-targeted genes of probable green algal origin encoded by the nucleus of *L. chlorophorum *presented in this study, strongly indicates that the green algal plastid is a permanent and fully integrated organelle. Among the transferred plastid-targeted genes are genes characteristic of the green lineages, such as RuBisCO activase and components of the photosynthetic pathway (psbR, psbP), which have never previously been detected in dinoflagellates. All *L. chlorophorum *plastid-targeted proteins have been extended by a signal peptide to transport the protein across the additional membranes resulting from the secondary endosymbiosis, and are thus likely functional and transported to the organelle, demonstrating that the plastid is indeed stable and dependent on the host. This means that full plastid replacement events have occurred on at least two occasions among dinoflagellates (*Lepidodinium *and *Karlodinium/Karenia*) [9,23,24,38].

In addition to genes originating from the current green plastid, we also identified putative plastid-targeted genes (with N-terminal extensions) that clustered with genes from the peridinin plastid in phylogenetic trees, implying that proteins from the ancient endosymbiont are still functional and now targeted to the new plastid. This shows that nuclear-encoded plastid-targeted protein genes can be retained even though the plastid itself is replaced, providing further support for the view that a plastid replacement event does not necessarily lead to loss of all characters from the previous plastid as earlier predicted [31,48,49]. The retention of "old" genes in the new plastid proteome mirrors the results of studies of the transcriptome of *K. veneficum *and *K. brevis*, which harbor tertiary haptophyte-derived plastids [31,33]. Together, these results show that combining genes transferred via endosymbiotic gene transfer (EGT) from the new plastid and recycled genes from the ancient endosymbiont is a general trait among dinoflagellate lineages that have undergone repeated uptake and loss of plastids. This supports that the ancient plastid plays a role in the replacement process and likely facilitates integration of the new plastid [4].

It is unclear whether the original peridinin plastid of *L. chlorophorum *was replaced while still photosynthetically functional, or if the new plastid was established after the lineage had lost its ability to photosynthesize or lost its plastids altogether. In *K. veneficum*, the apparent lack of peridinin-plastid derived photosynthesis genes suggests that its ancestor was heterotrophic when the new haptophyte plastid was acquired, and that the relic plastid was retained at that time for anabolic purposes [31]. In contrast, identification of the ancient dinoflagellate form of ferredoxin NADP reductase (an iron-sulfur protein involved in several photosynthetic reactions) in *L. chlorophorum *implies that the green plastid was engulfed either while the host was still photosynthetic, or very soon after its ability to photosynthesize was lost.

### Horizontal or endosymbiotic gene transfer?

The retention of nuclear-encoded genes from the peridinin-containing plastid in the *L. chlorophorum *plastid proteome is analogous with the situation in *K. veneficum *and *K. brevis *[31,33]. However, the *L. chlorophorum *nucleus also contains plastid-targeted genes of other origins, including streptophytes, heterokonts and haptophytes, indicating that this lineage may be substantially affected by horizontal gene transfer (HGT). While HGT is well known in prokaryotes and have been identified in other dinoflagellate lineages [50], the significant impact of gene transfers in eukaryotic evolutionary history has only recently been recognized [51,52]. Another plastid proteome that seems to be heavily affected by HGT is the chlorarachniophyte *B. natans*, in which about 20% of the plastid-targeted genes originate from sources other than its current green algal plastid [15]. The high degree of 'foreign' genes in *B. natans *has been suggested to be a result of horizontal transfer associated with the mixotrophic mode of nutrition of this organism [15]. The analogous pattern of horizontally transferred genes in dinoflagellates might also be due to this type of life style [53]. Recently, a distinct footprint of a green algal lineage (the prasinophytes) was revealed in major chromalveolate lineages, suggesting that a cryptic endosymbiont was present in the their ancestor [34]. If this theory is correct, at least 3 plastids (green, red, green) have been involved in shaping the plastid proteome of *L. chlorophorum*. Additionally, the high amount of foreign genes observed in *L. chlorophorum *and *B. natans *may reflect an ever more complex evolutionary history where genes were acquired via gene transfers from a series of cryptic endosymbionts. However, whether the *L. chlorophorum *genes originating from other sources than the known plastids were supplied by additional endosymbionts (EGT) or from prey (HGT) is difficult to infer without a larger dataset, and remains unclear.

### Import systems in mixed chloroplast proteomes

The green plastid in *L. chlorophorum *has changed environment from being a primary plastid with two surrounding membranes in green alga to becoming a secondary plastid in dinoflagellates with four enveloping membranes. Accordingly, we found that the presequences of the plastid-targeted proteins in *L. chlorophorum *are different from those usually observed in green algae (see Additional file 5 Figure S5).

Green algal targeting-transit peptides have elevated levels of hydroxylated Ser and Thr residues, a net positive charge due to depletion of acidic residues, and elevated levels of Ala [47]. However, in *L. chlorophorum*, the transit peptides of plastid-targeted proteins are depleted of Ser and Thr residues relative to those of green algae. The N-terminal extension of plastid-targeted proteins from peridinin-containing dinoflagellates comprises a FVA/SP-motif and two classes of sequences are recognized, of which class I contains an additional hydrophobic region that functions as a stop transfer membrane anchor (STMA) downstream of the transit peptide region (probably involved in a transportation pathway through the Golgi apparatus), while class II is deprived of a transmembrane region [54]. We did not observe the conserved FVAP motif common in dinoflagellates with peridinin-containing plastids (this is, however, somewhat uncertain due to the difficulties in estimating the cleavage sites). STMAs were not observed and the levels of acidic residues were higher than those found in other transit peptides (except for *K. veneficum*). The putative change of transit peptide characteristics is also seen in *K. veneficum*, whose transit signals are different from those of peridinin-containing plastids and those of haptophyte plastids [31]. It is interesting to note that both dinoflagellate lineages with replaced plastids most likely use a new type of transit peptide rather than recycling the transit peptide from the ancestral condition or the endosymbiont. These modifications may have been driven by the co-existence of two divergent plastids after the uptake of a new plastid, which required a way to discriminate between the plastid genes [31]. Accordingly, it is intriguing to speculate that modification of N-terminal presequences is related to the mosaic plastid proteomes which have co-evolved and adapted to the trafficking machinery of the host [55].

## Conclusions

In this study, we investigated the evolutionary origin of the plastid-targeted proteins of *L. chlorophorum *and analyzed their N-terminal presequences that are likely associated with plastid targeting. The plastid proteome of *L. chlorophorum *is a mixture of proteins with different phylogenetic origins. This hybrid proteome is seemingly shaped by the current plastid, the previous peridinin-type plastid and by horizontally transferred genes from various algal lineages. However, additional cryptic endosymbioses involving additional algal groups (streptophytes, heterokonts, haptophytes) cannot be ruled as the causes of phylogentic proteome mosaics. The *L. chlorophorum *transit peptides differ from the transit peptides found in green algae or other dinoflagellates. The characteristics of the altered transit peptides found in *L. chlorophorum *as well as *Karenia/Karlodinium *are likely to have played an important role for shaping the mosaic plastid proteome of these species.

## Methods

### Culturing, library construction, cDNA-sequencing and 5' RACE

The monoclonal *L. chlorophorum *strain RCC1488 (Roscoff Culture Collection: http://www.sb-roscoff.fr/Phyto/RCC) was cultured in K/2(-Tris,-Si) medium [56] at 17°C with illumination provided by daylight neon tubes at an intensity of 150 μE.m^2^.s^1 ^and a photoperiod of 14L:10 D. Pre-cultures were treated for 24 hours with a range of concentrations of Provasoli antibiotic mixture (Sigma Aldrich) and sub-cultured regularly over a 3-week period in order to minimize bacterial contamination. Two 10 liter cultures were harvested in mid to late exponential growth phase by centrifugation (5 min at 6,000 rpm) in sterile 1 liter polycarbonate centrifuge flasks (Nalgene). Cell concentrates were immediately flash frozen in liquid nitrogen and stored at -80°C pending analysis.

RNA was isolated and a non-normalized, directional, cDNA library was constructed in the plasmid vector pAGEN-1 by Agencourt Bioscience Corp. (Beverly, MA, USA). 4,746 randomly picked clones were 5'-end sequenced, and subsequently quality checked and assembled to contigs using a Phred/Phrap pipeline at the open-access Bioportal service at University of Oslo http://www.bioportal.uio.no. BLASTx analyses http://www.ncbi.nlm.nih.gov/BLAST and gene annotation of *L. chlorophorum *singletons and contigs were performed using Blast2GO [57].

The 5'-ends of the cDNA sequences were amplified by performing rapid amplification of cDNA ends (RACE), using the GeneRacer kit with SuperScript III RT (Invitrogen, Carlsbad, USA) using specific primers and a nested PCR approach. The products were subsequently cloned using the TOPO-TA cloning kit for sequencing (Invitrogen, Carlsbad, USA).

### Identification of plastid targeted sequences

Plastid targeted sequences among singletons and contigs were identified according to their phylogenetic relationship to other plastid homologs, participation in plastid-located processes, and/or possession of plastid-targeted sequence comprising a signal peptide and a transit peptide [15]. Searches for possible signal peptides and their cleavage sites were estimated using SignalP (http://www.cbs.dtu.dk/services/SignalP[45]. Logoplots were constructed using WebLogo http://weblogo.berkeley.edu/logo.cgi, while transmembrane helices were identified using TMHMM server v.2.0 http://www.cbs.dtu.dk/services/TMHMM and the hydrophobicity profile of Kyte and Doolittle [58].

### Alignment construction and phylogenetic analyses

Amino-acid sequence alignments were created by downloading homologous sequences from the nonredundant (NCBInr) and EST (NCBIest) databases in GenBank for each putative plastid-targeted gene detected in *L. chlorophorum*. We aimed to include taxa from all main photosynthetic eukaryotic lineages in each alignment. The sequences were initially aligned using MAFFT [59], and subsequently edited manually in MacClade [60]. Ambiguously aligned characters were detected by eye and removed manually. Maximum likelihood trees were inferred using RAxML [61]. Trees were identified with 100 separate heuristic searches from random starting trees, while bootstrap analyses involved 100 pseudoreplicates and one heuristic search for each replication with the same substitution model as the initial search. Bayesian analyses were performed using MrBayes v.3.1.2 [62,63] Trees were generated from two runs with one heated and three cold chains in the MCMC, using a random starting tree and 2,000,000 generations. Tree sampling were done every 100 generations. Burn-in trees were set according to the assessment of the likelihood plots and the convergence diagnostics implemented in MrBayes, and the consensus of the sampled trees were used to calculate the posterior probabilities. The best fitting evolutionary model for each alignment according to the AIC (for ML) and BIC (for Bayesian) was applied (i.e. WAG, RtRev or CtRev;. estimated by Prottest v.2.4 [64]).

Accession numbers for the sequences used in the alignments are shown in Additional file 6 Table S1.

## Authors' contributions

MAM screened the EST library for plastid-associated genes, carried out the contig assembly, alignment construction and phylogenetic analyses, helped with the 5'-RACE experiments and wrote the manuscript. KST helped to design the study and contributed to the manuscript. OKT and KT performed 5'-RACE experiments and analyzed the signaling sequences. IP carried out the algal culturing. OKT, YI, KT and IP contributed to the manuscript. DK was involved in scientific design and technical discussions. KSJ conceived the study, was the project leader and contributed to the manuscript. All authors read and approved the final manuscript.

## Supplementary Material

Additional file 1 **Supplementary Figure S1: Genes of red algal origin**. Maximum likelihood trees inferring a red-algal origin of 4 plastid-associated genes. All trees were inferred using RAxML. Bootstrap values >50%are indicated on the branches. Green and red lineages are indicated by color, secondary green algae are in bold. Filled dots indicate 100% bootstrap support. Bayesian posterior probability values are indicated for some of the most important splits. Click here for file

Additional file 2 **Supplementary Figure S2: Unresolved phylogenies**. Maximum likelihood trees demonstrating an unresolved position for *L. chlorophorum*. Filled dots indicate 100% bootstrap support. Bayesian posterior probability values are indicated for some of the most important splits. Click here for file

Additional file 3 **Supplementary Figure S3: Kyte-Doolittle hydrophobicity plots of the N-terminal extensions (presequences)**. Click here for file

Additional file 4 **Supplementary Figure S4:Signal peptide sequence.** Weblogo plot of the putative signal peptides sequence estimated using SignalP-HMM. Click here for file

Additional file 5 **Supplementary Figure S5: Amino acid composition of transit peptides**. Percentage bars of transit peptides of chlorophytes, peridinin-containing dinoflagellates, *K. veneficum *(all derived from Patron & Waller 2007), *L. chlorophorum *and the entire *L. chlorophorum *mature peptide. Click here for file

Additional file 6 **Supplementary Table S1: Accession numbers of sequences used in phylogenetic analyses**. Click here for file
