# Supplementary figures and images for: A phylogenetic mosaic plastid proteome and unusual plastid-targeting signals in the green-colored dinoflagellate Lepidodinium chlorophorum
Source: BMC Evol Biol. 2010 Jun 21;10:191. doi: 10.1186/1471-2148-10-191 (PMC3055265; doi:10.1186/1471-2148-10-191)

Fructose-1,6-bisphosphatase

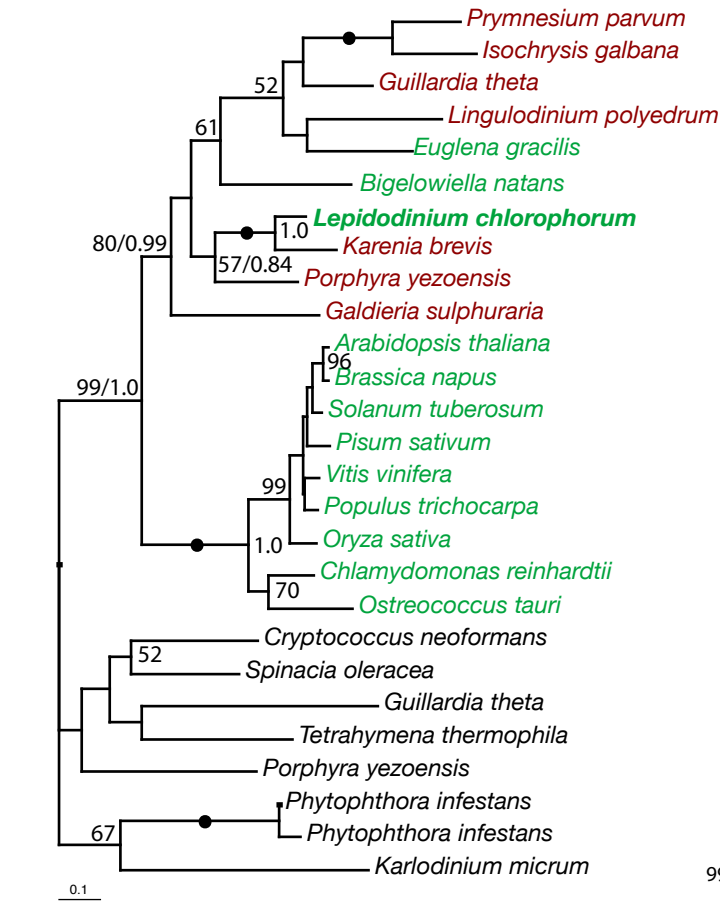

Transketolase

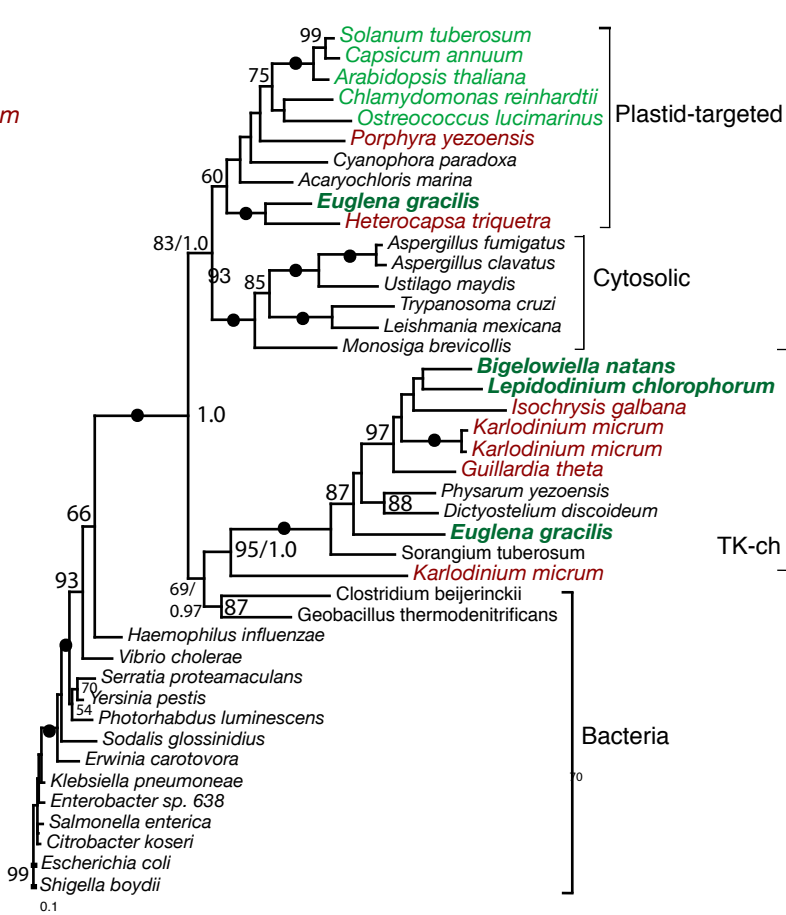

ATP synthase gamma

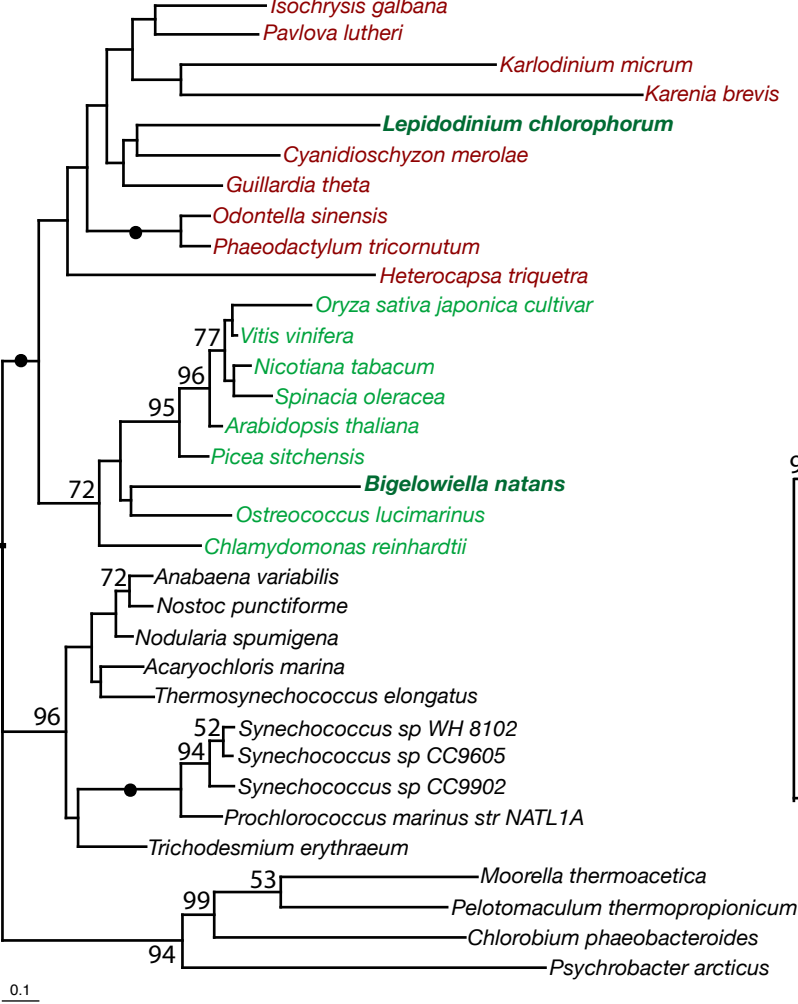

Hcf136

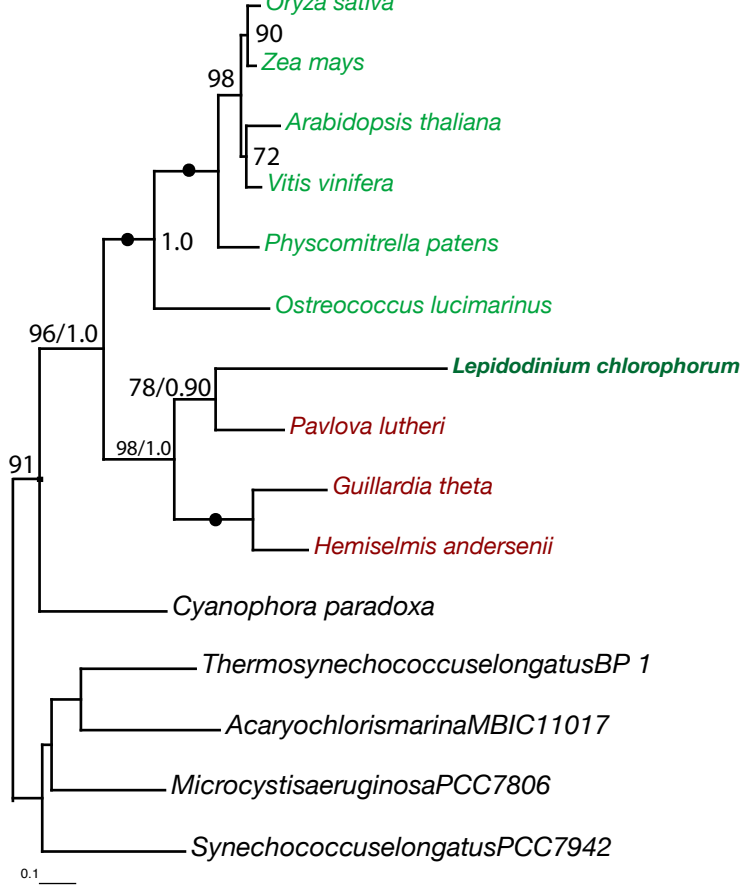

Supplement: Additional file 1 — Supplementary Figure S1: Genes of red algal origin. Maximum likelihood trees inferring a red-algal origin of 4 plastid-associated genes. All trees were inferred using RAxML. Bootstrap values >50%are indicated on the branches. Green and red lineages are indicated by color, secondary green algae are in bold. Filled dots indicate 100% bootstrap support. Bayesian posterior probability values are indicated for some of the most important splits. [file 1471-2148-10-191-S1.PDF]

### 3,8 divinyl protochlorophyllide

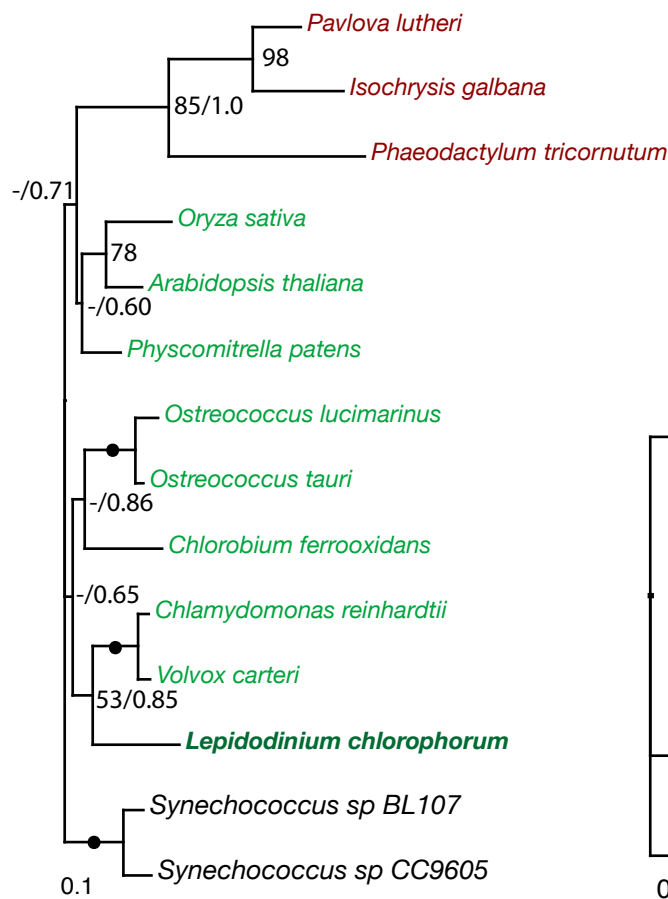

### Sedoheptulose bisphosphatase

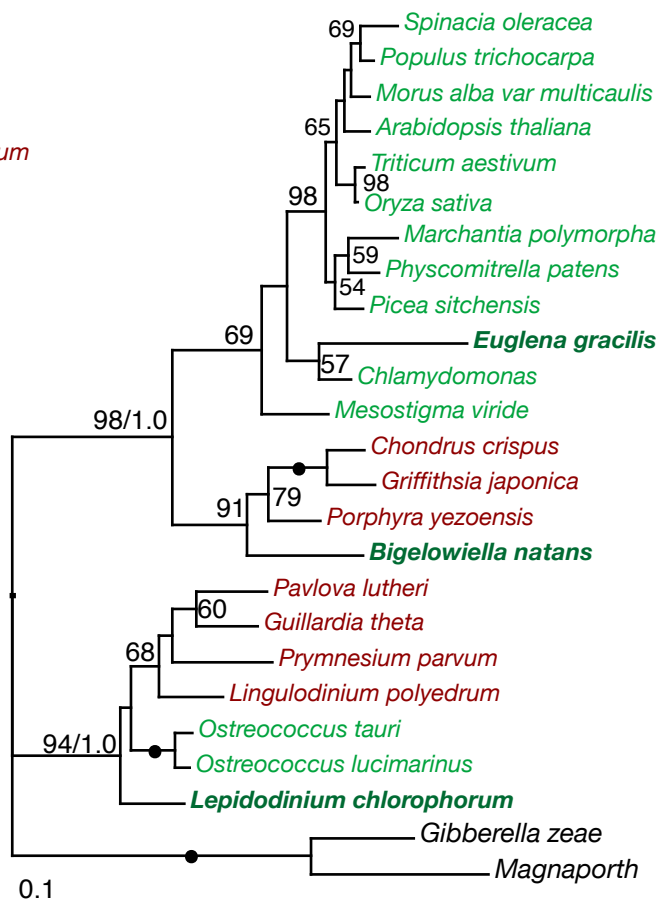

### DnaJ/Hsp40

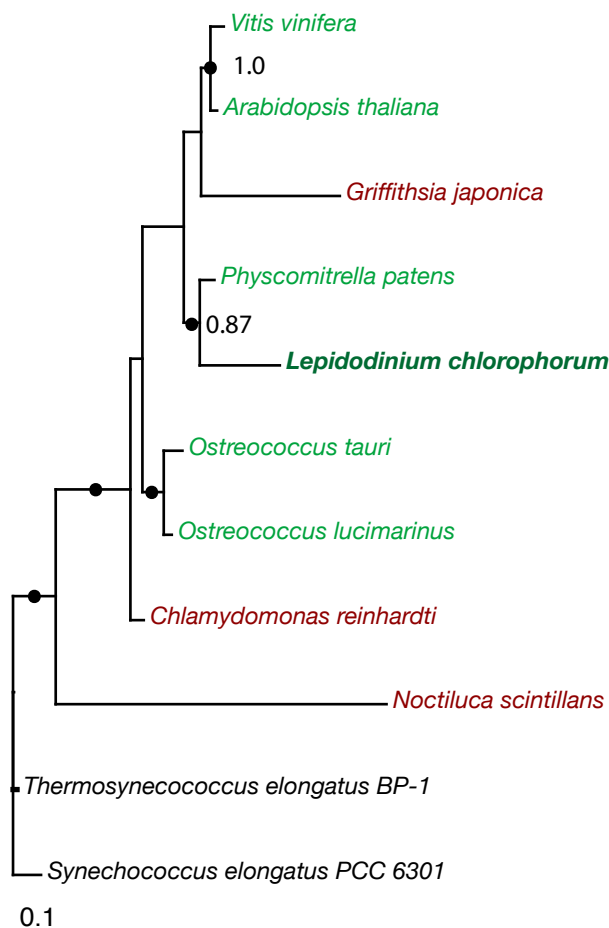

### Ferredoxin B

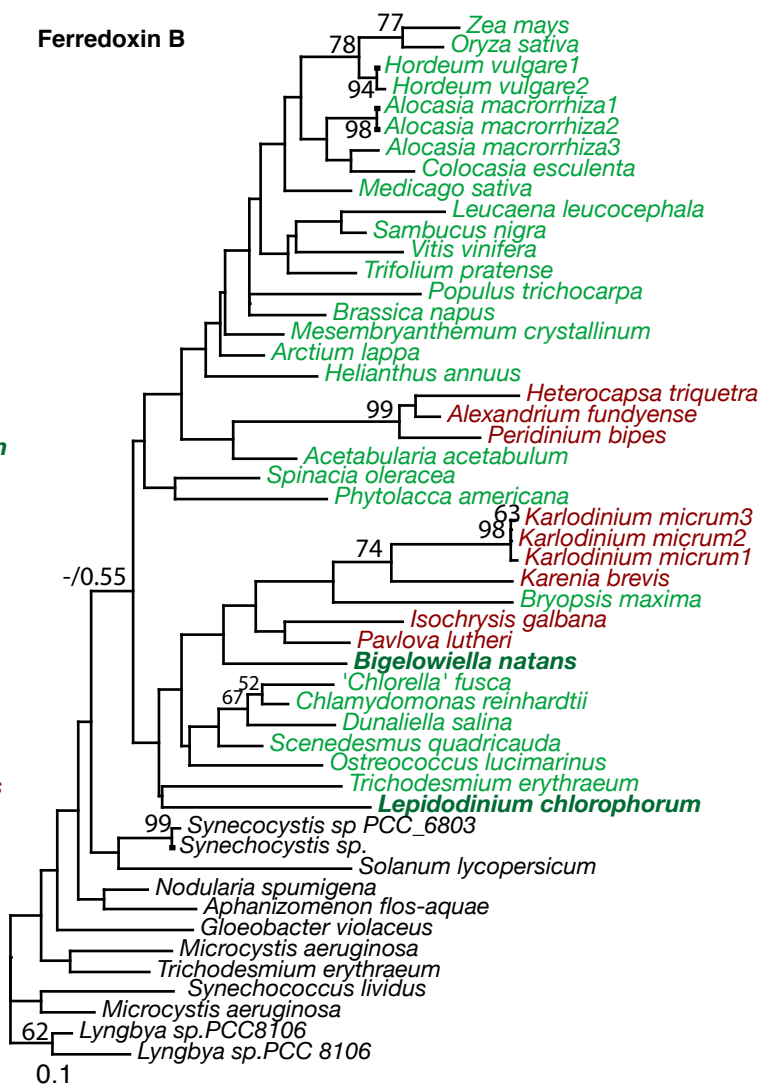

Supplement: Additional file 2 — Supplementary Figure S2: Unresolved phylogenies. Maximum likelihood trees demonstrating an unresolved position for L. chlorophorum. Filled dots indicate 100% bootstrap support. Bayesian posterior probability values are indicated for some of the most important splits. [file 1471-2148-10-191-S2.PDF]

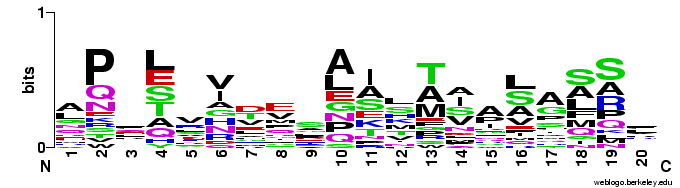

Supplement: Additional file 4 — Supplementary Figure S4:Signal peptide sequence. Weblogo plot of the putative signal peptides sequence estimated using SignalP-HMM. [file 1471-2148-10-191-S4.PNG]

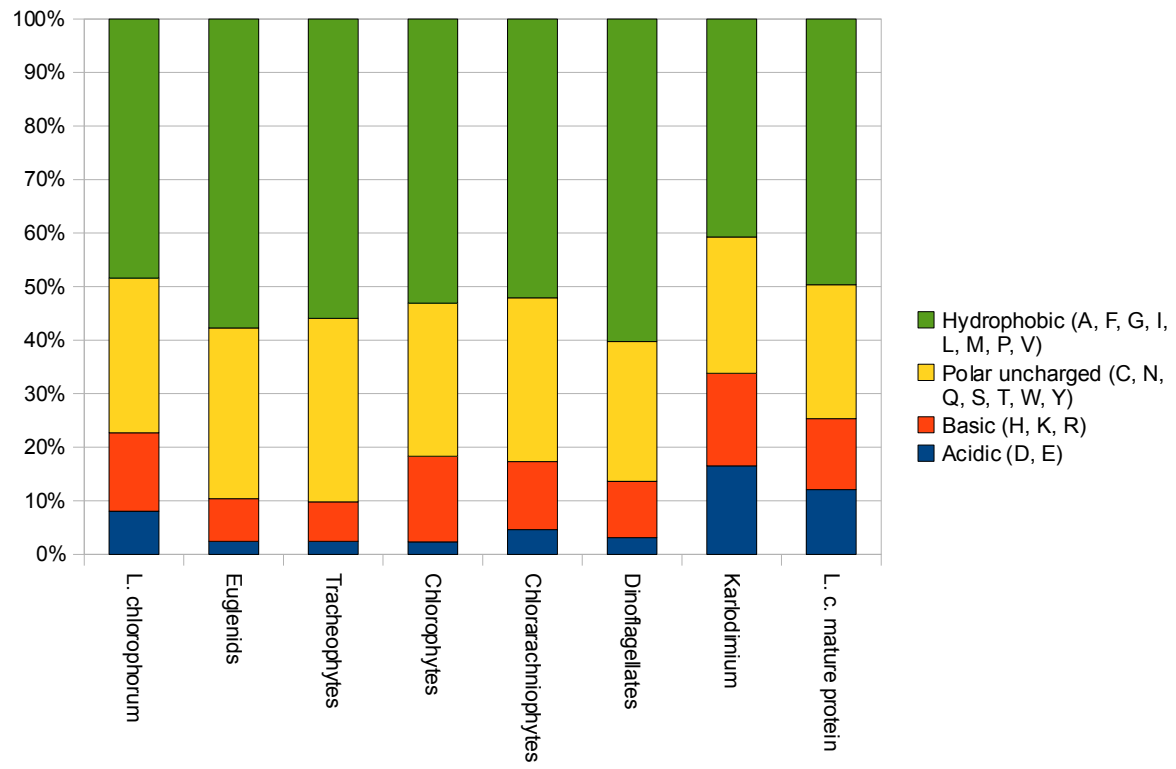

Supplement: Additional file 5 — Supplementary Figure S5: Amino acid composition of transit peptides. Percentage bars of transit peptides of chlorophytes, peridinin-containing dinoflagellates, K. veneficum (all derived from Patron & Waller 2007), L. chlorophorum and the entire L. chlorophorum mature peptide. [file 1471-2148-10-191-S5.PDF]
