# Supplementary material for: A phylogenetic mosaic plastid proteome and unusual plastid-targeting signals in the green-colored dinoflagellate Lepidodinium chlorophorum
Source: BMC Evol Biol. 2010 Jun 21;10:191. doi: 10.1186/1471-2148-10-191 (PMC3055265; doi:10.1186/1471-2148-10-191)

>atpc_signal_transit_sequence

MSVITRCVAVALSAVAAAANFQPVVSSGKTFSPFGLRGDSRRSSG

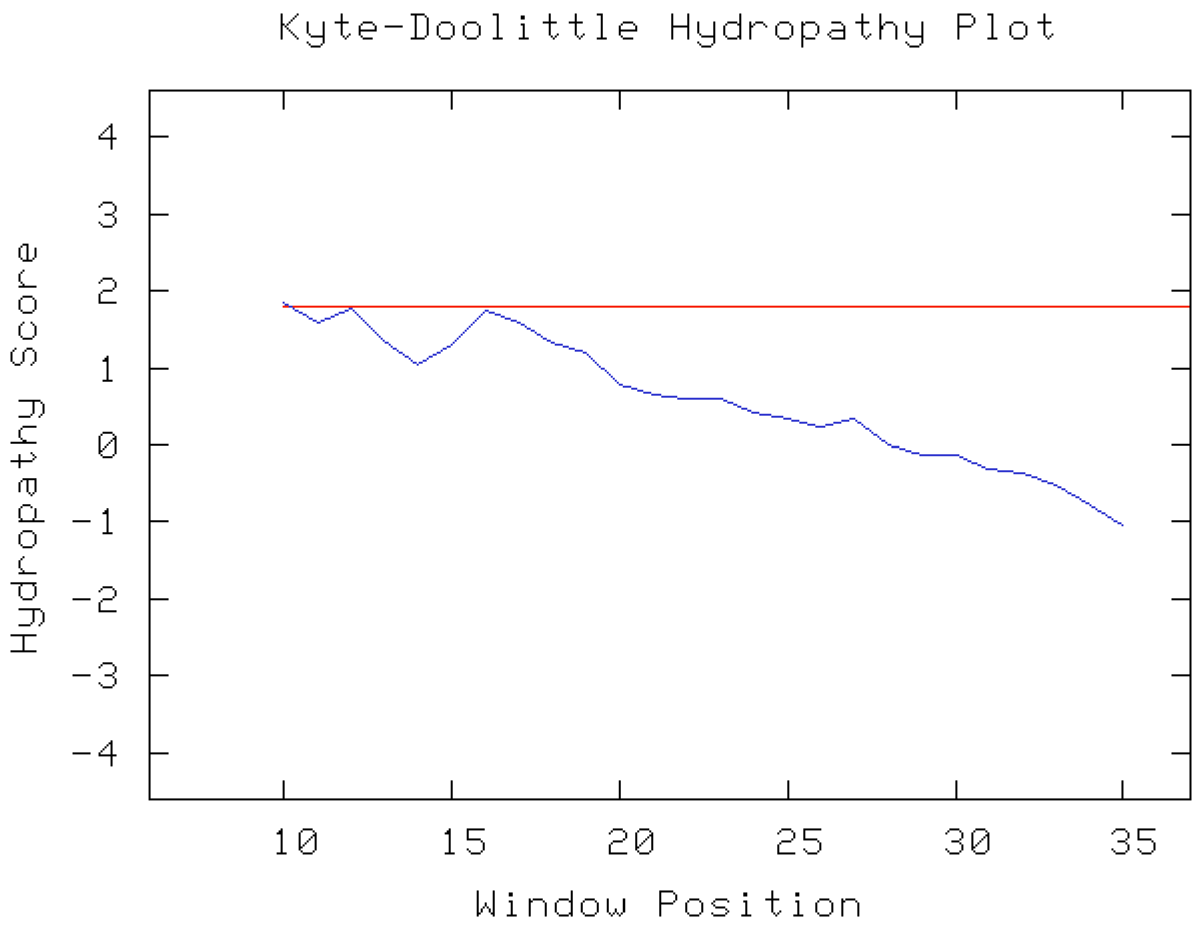

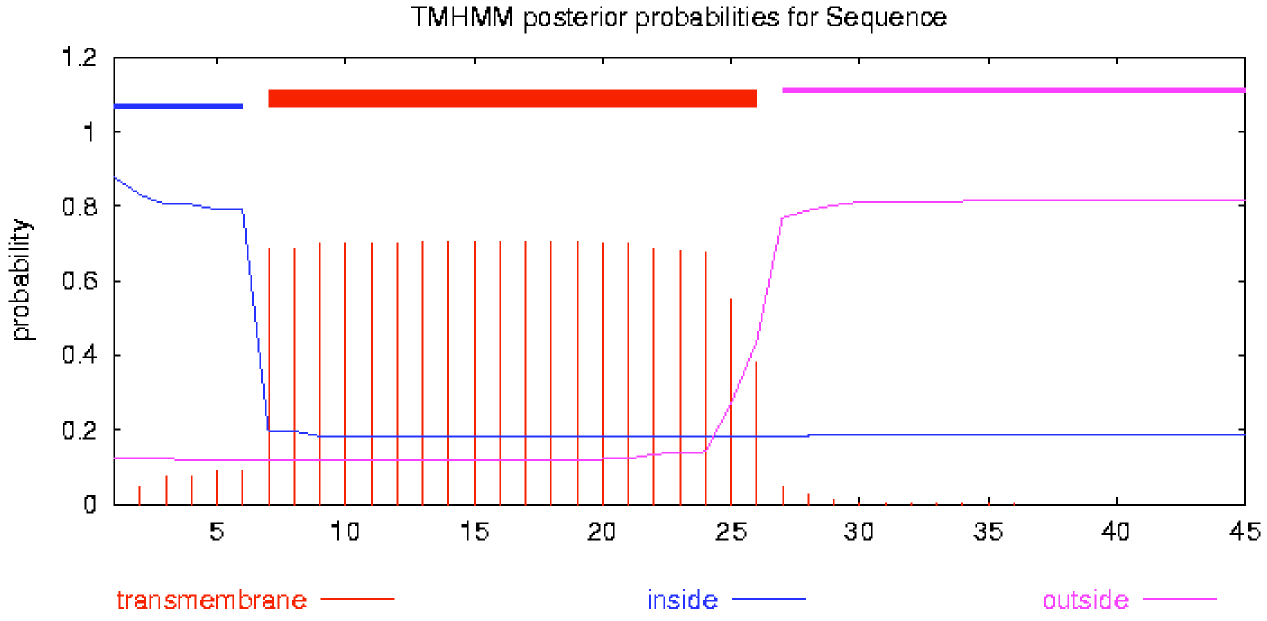

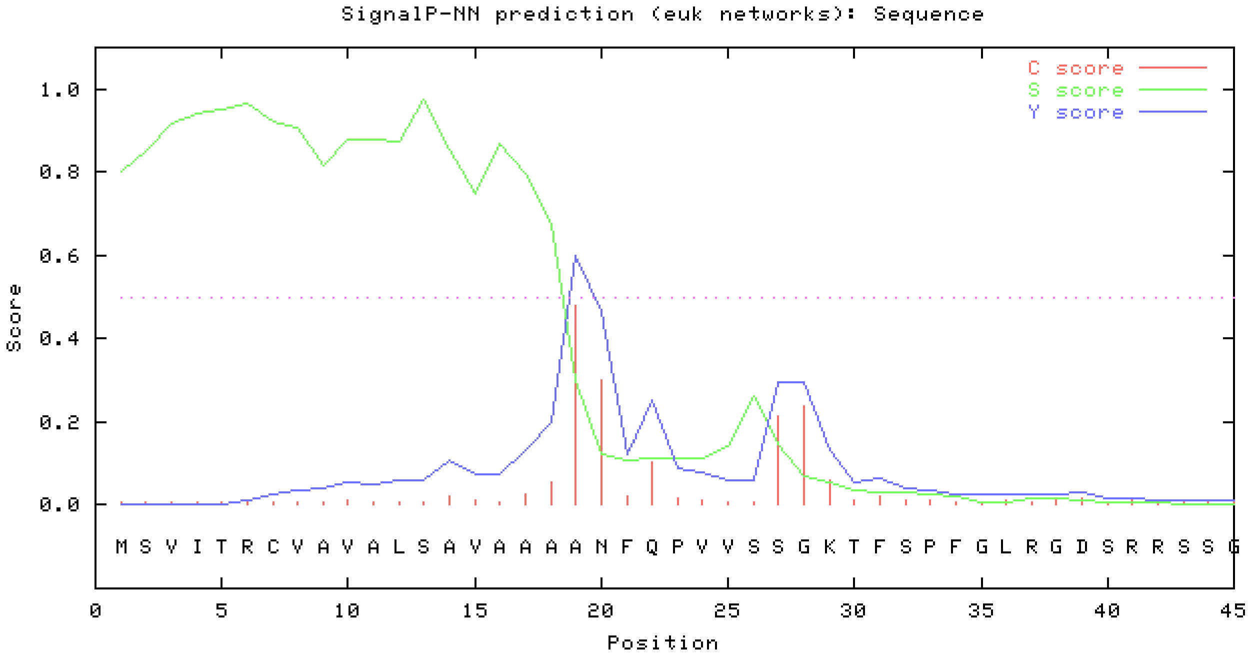

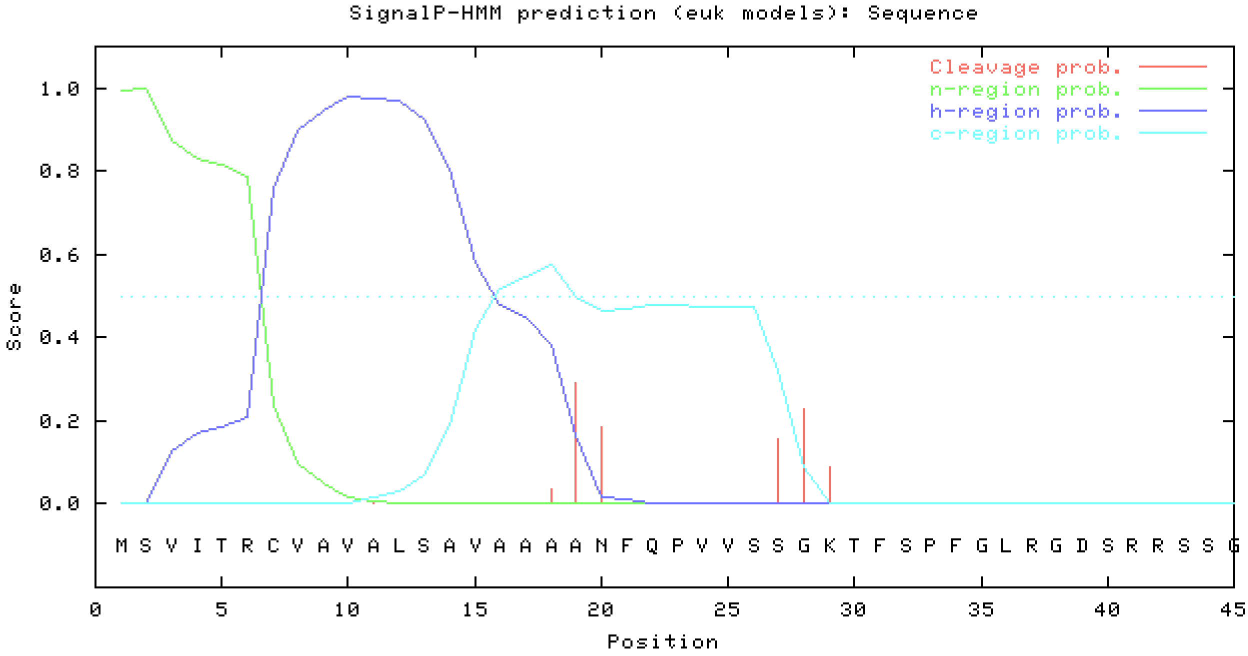

>chlpg_signal_transit_sequence

MSVQAALLDGDAERNTYARFFKAGVAFISGSCLAFLGVTAAVSTYTHTLHSSNAVESASLLDMAAIRGPSMTLLNLPGAS
PWKELAIAGIESSGQCSRDVSSNAFKKVMSTMSTAEVTAVEKAEEVVQKKAEDILK

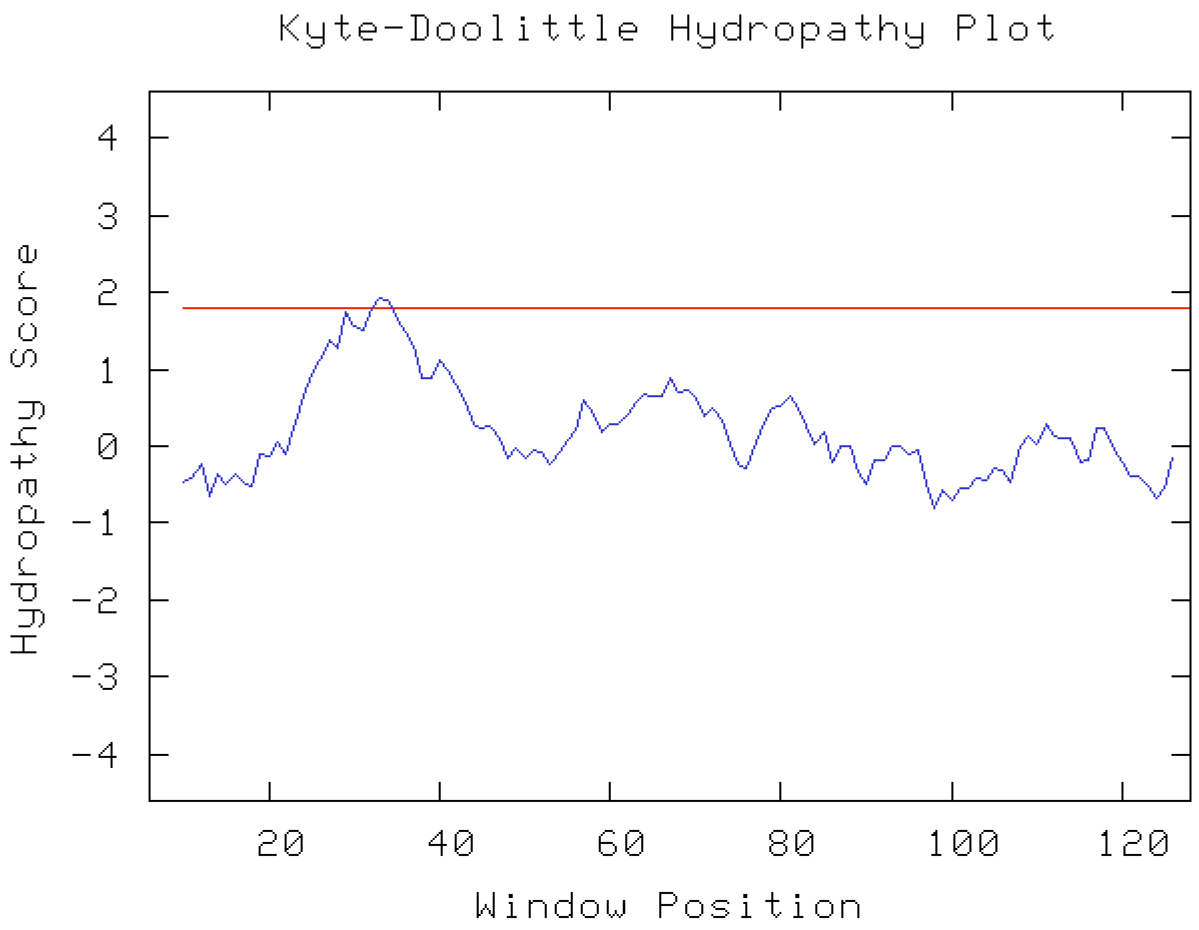
Kyte-Doolittle Hydropathy Plot
Hydropathy Score
Window Position
4
3
2
1
0
-1
-2
-3
-4
20
40
60
80
100
120

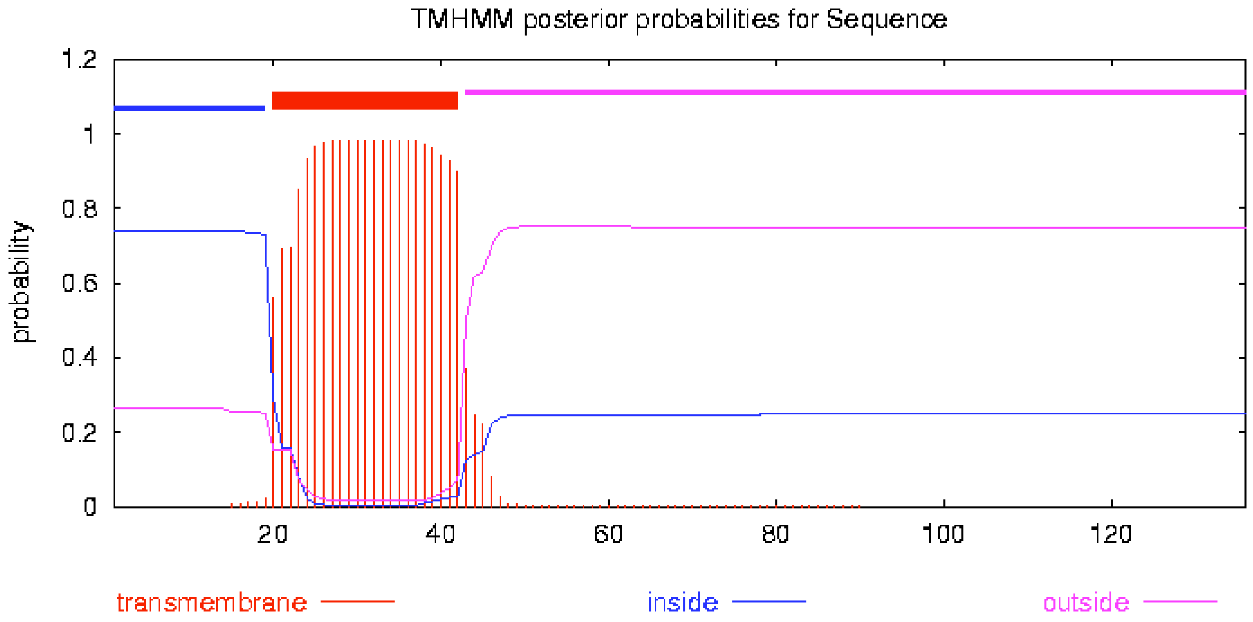
TMHMM posterior probabilities for Sequence
probability
1.2
1
0.8
0.6
0.4
0.2
0
20
40
60
80
100
120
transmembrane
inside
outside

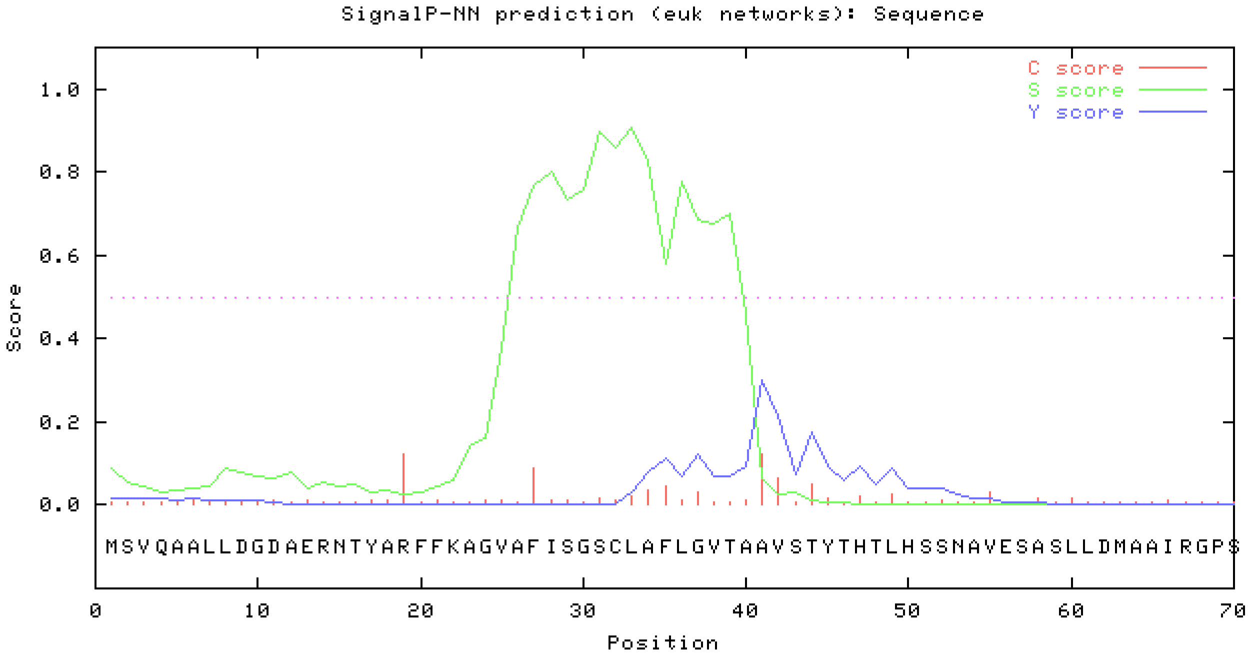

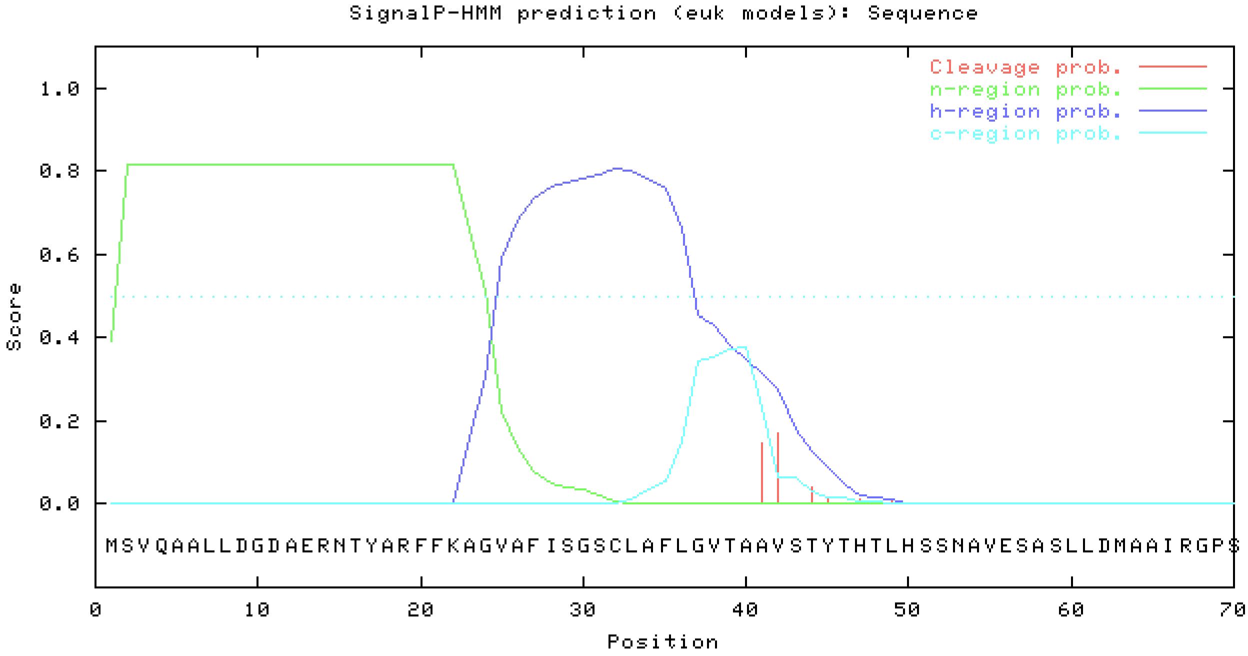

After N-20aa cut

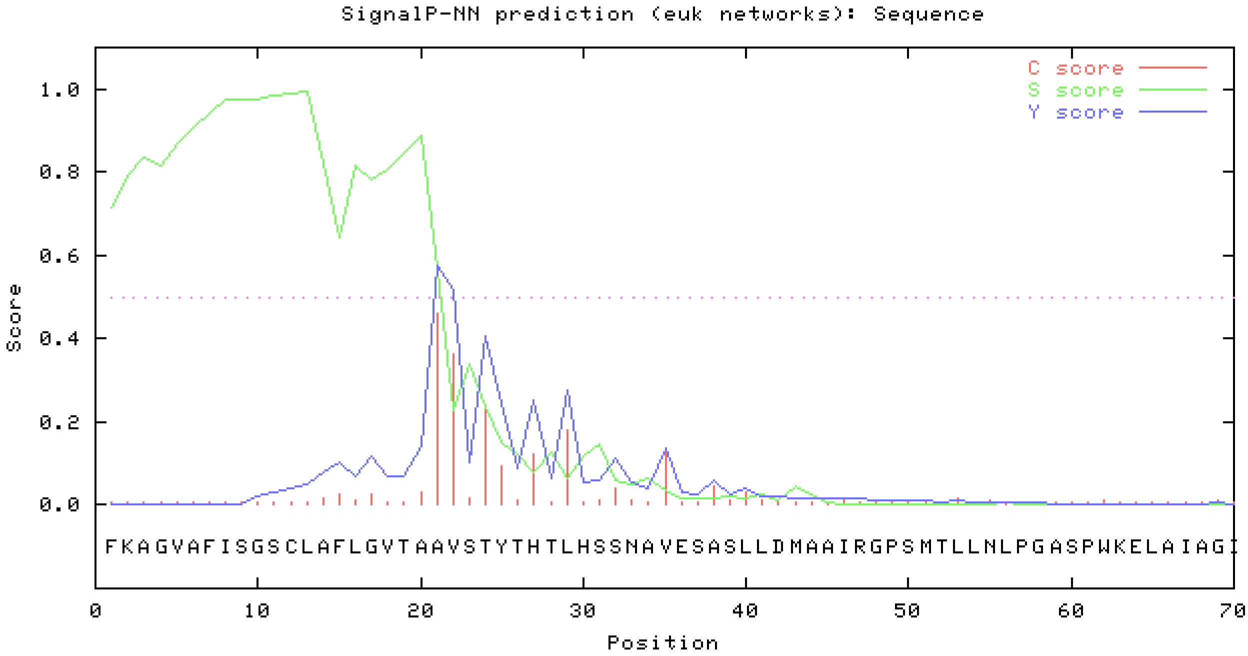

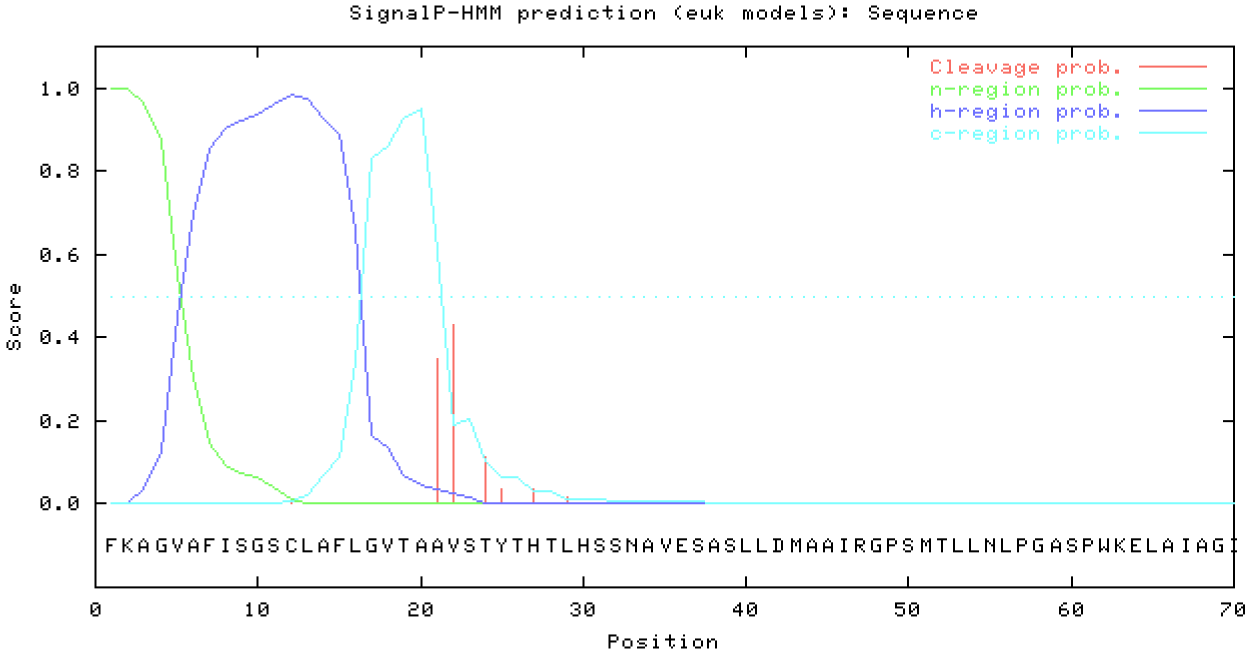

>cmk_signal_transit_sequence

MCCATAPRRASAVAATATAAALALAASSATTFAAFRSVHAAAPETLGGTSPSKRPIVSAAREGARTTCRRWGPRGFLAML

GLVAAQRRRPRKCRMRHGNRLRHQSLVERAADPQEGKS

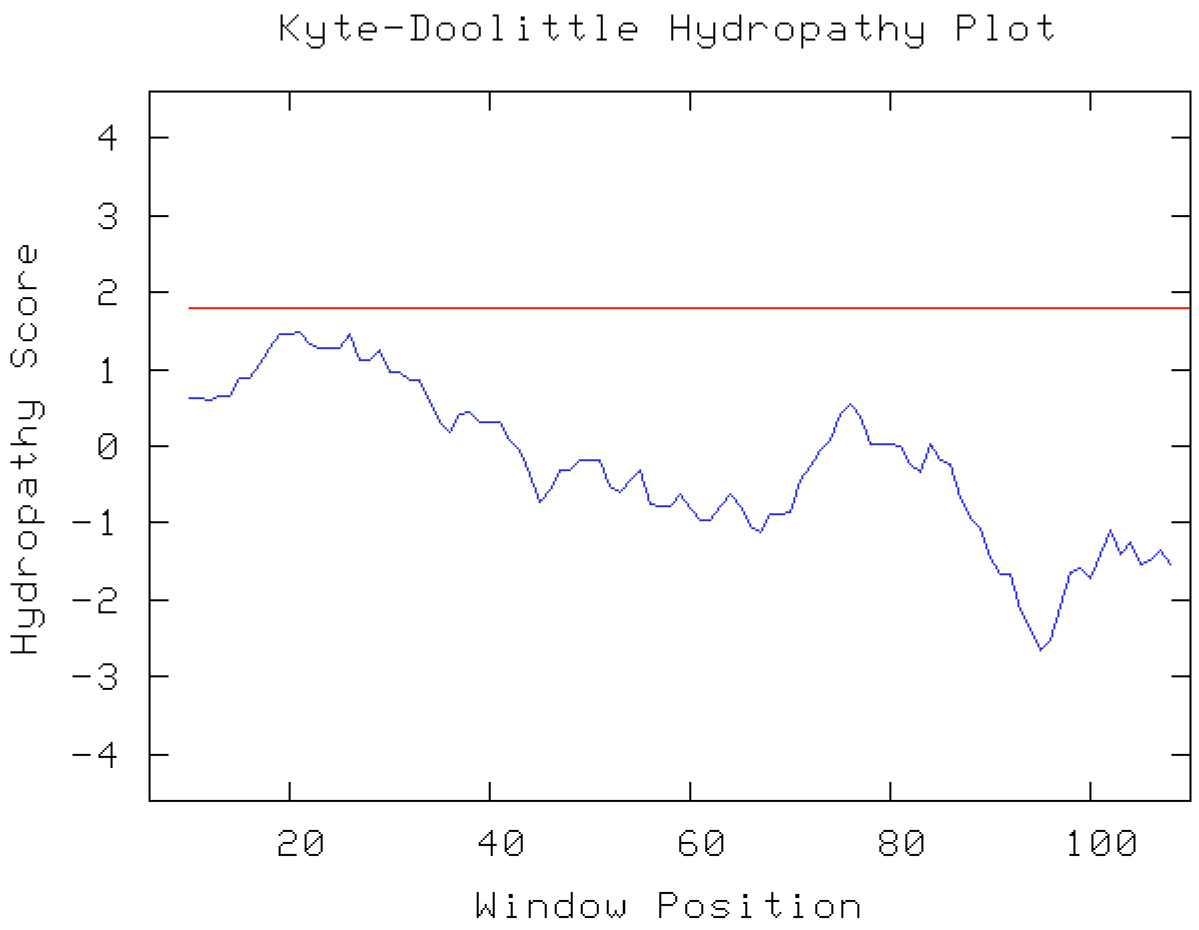
Kyte-Doolittle Hydropathy Plot
Hydropathy Score
4
3
2
1
0
-1
-2
-3
-4
20
40
60
80
100
Window Position

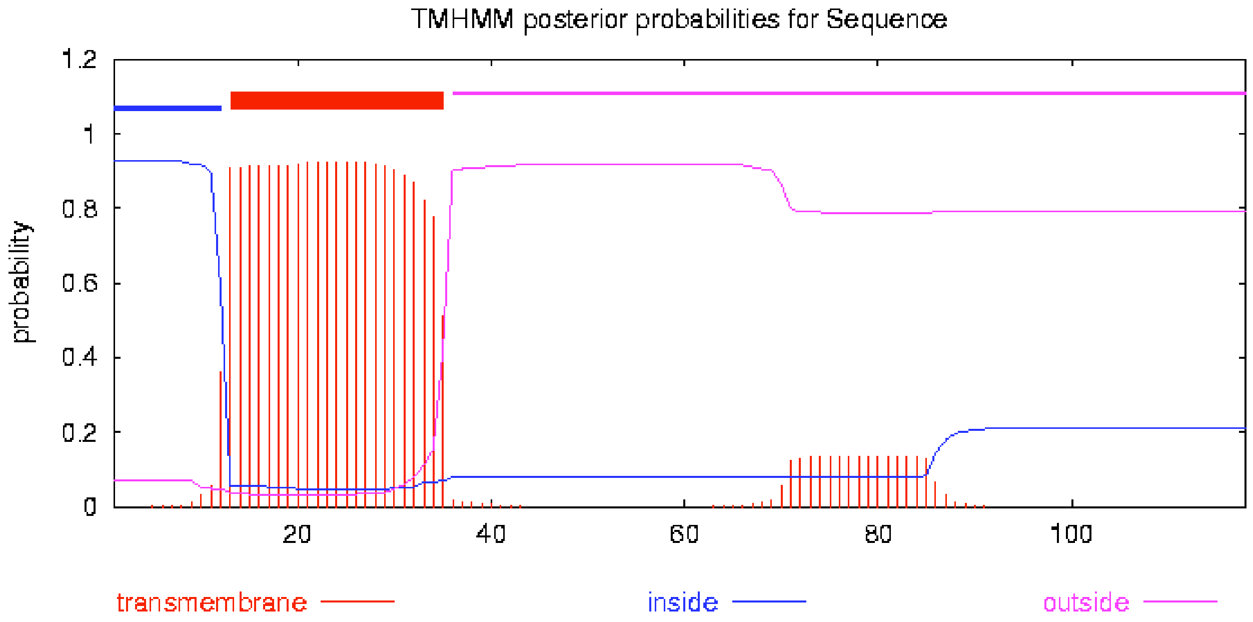
TMHMM posterior probabilities for Sequence
probability
1.2
1
0.8
0.6
0.4
0.2
0
20
40
60
80
100
transmembrane
inside
outside

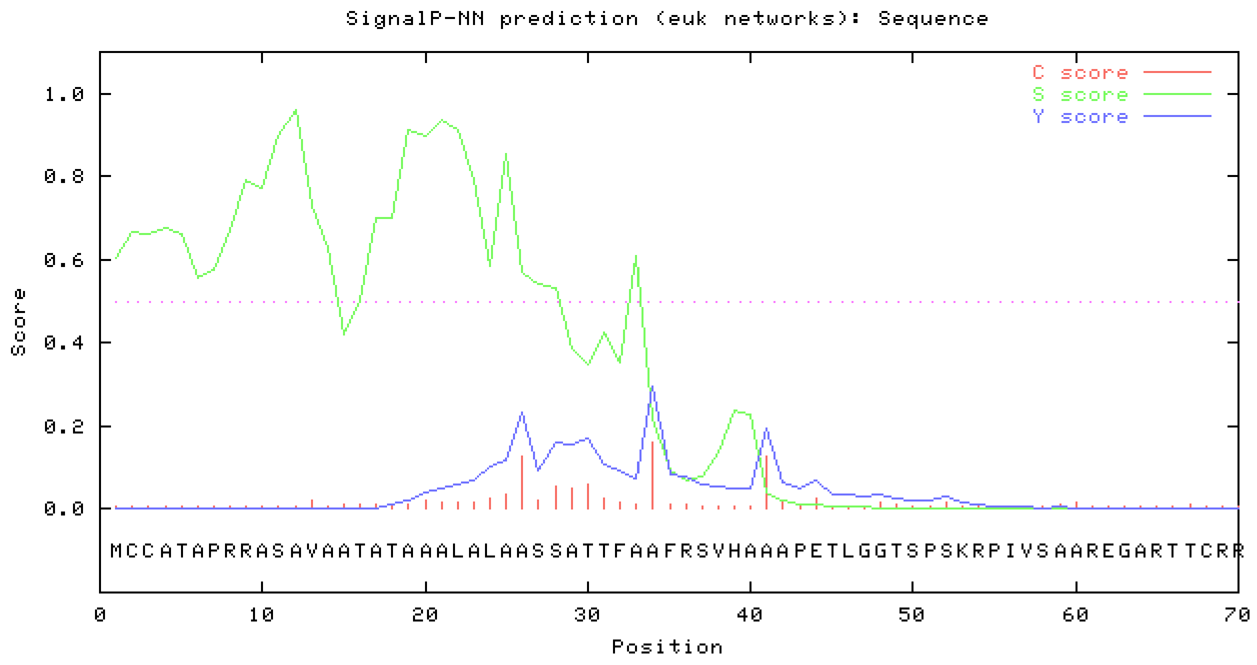

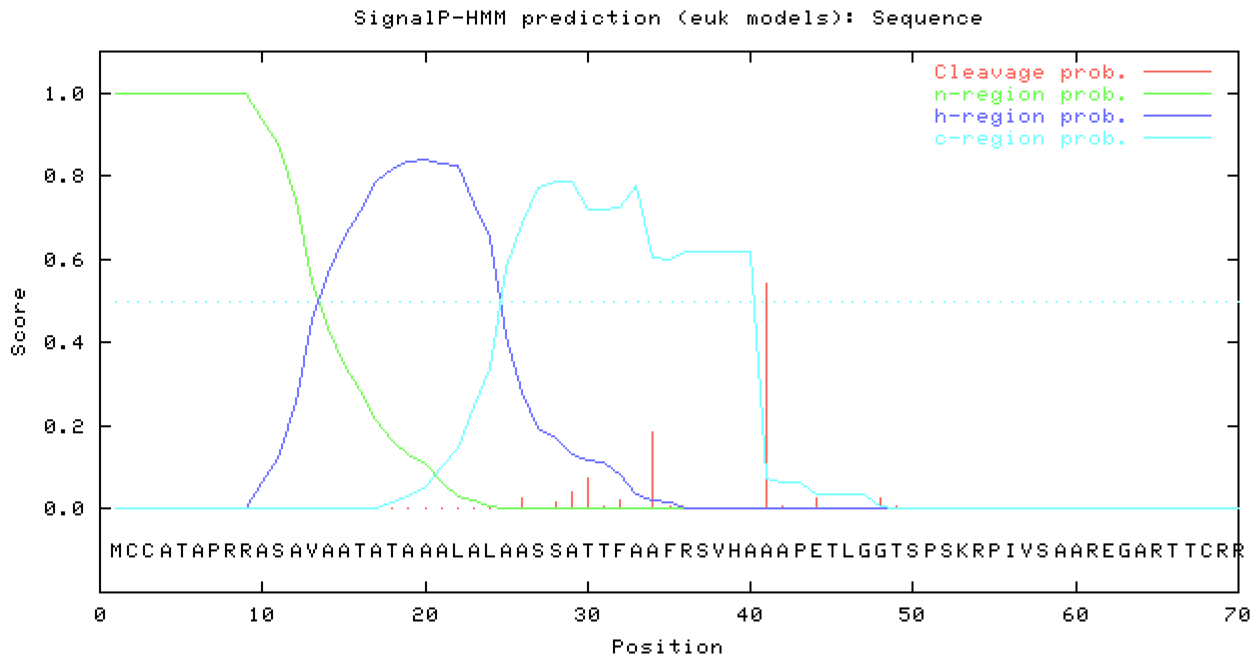

After N-12aa cut

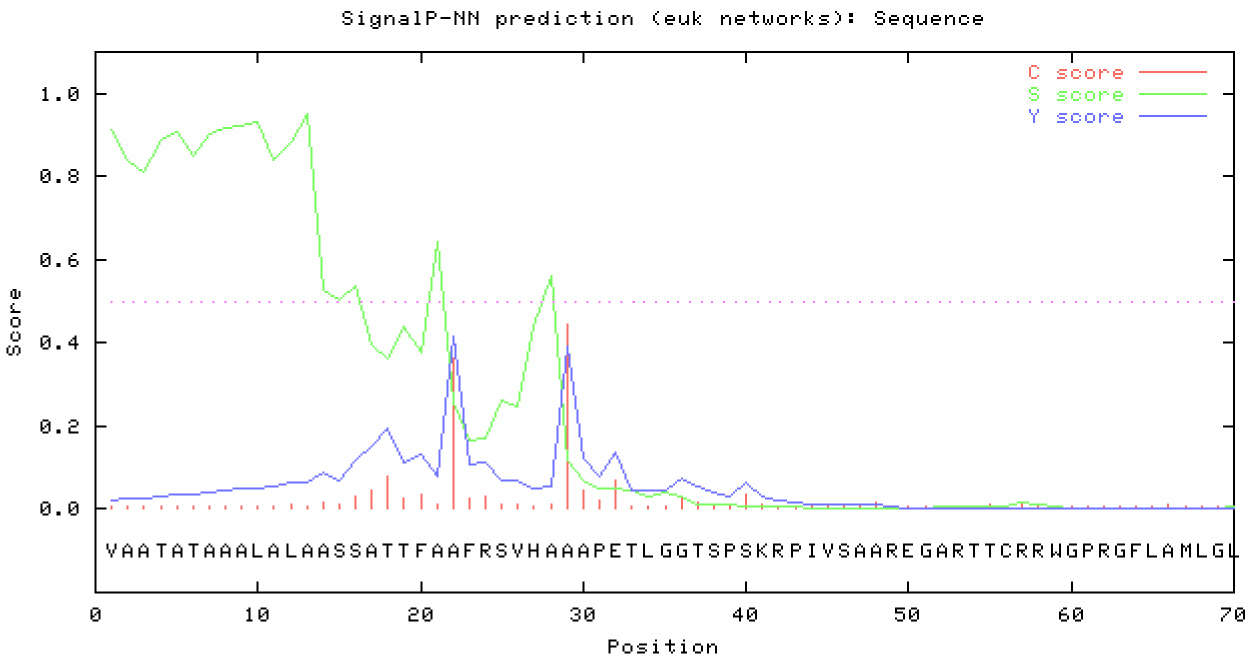

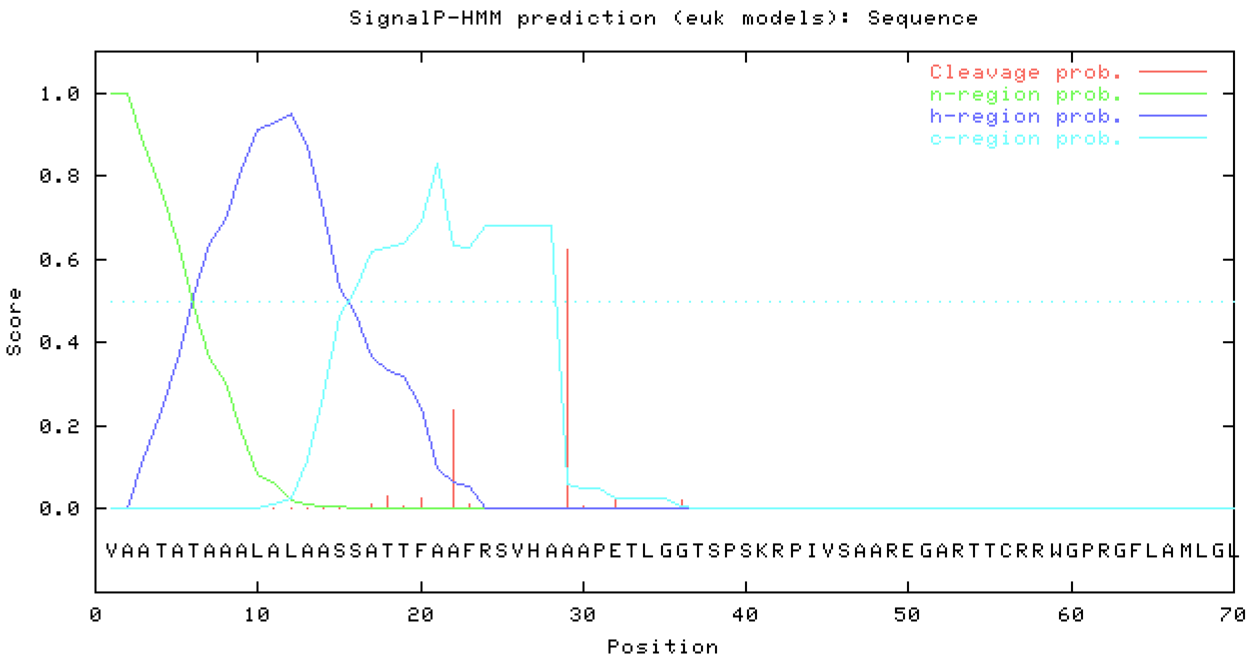

>csp41_signal_transit_sequence

MLRDVVLLLLLLAVIARTHA*AEEQEVNHMLNEHSDGLANRMSQPAVVQHADLDGATLGKTSQFAPHMSRIAQSTMSSSS
VTGYSTCRLAPLSRSFSKYTPQYNPAYYPNQGSNKKTTGGGSPLPMWISTEGFSQEQLDFLQRRQADKG

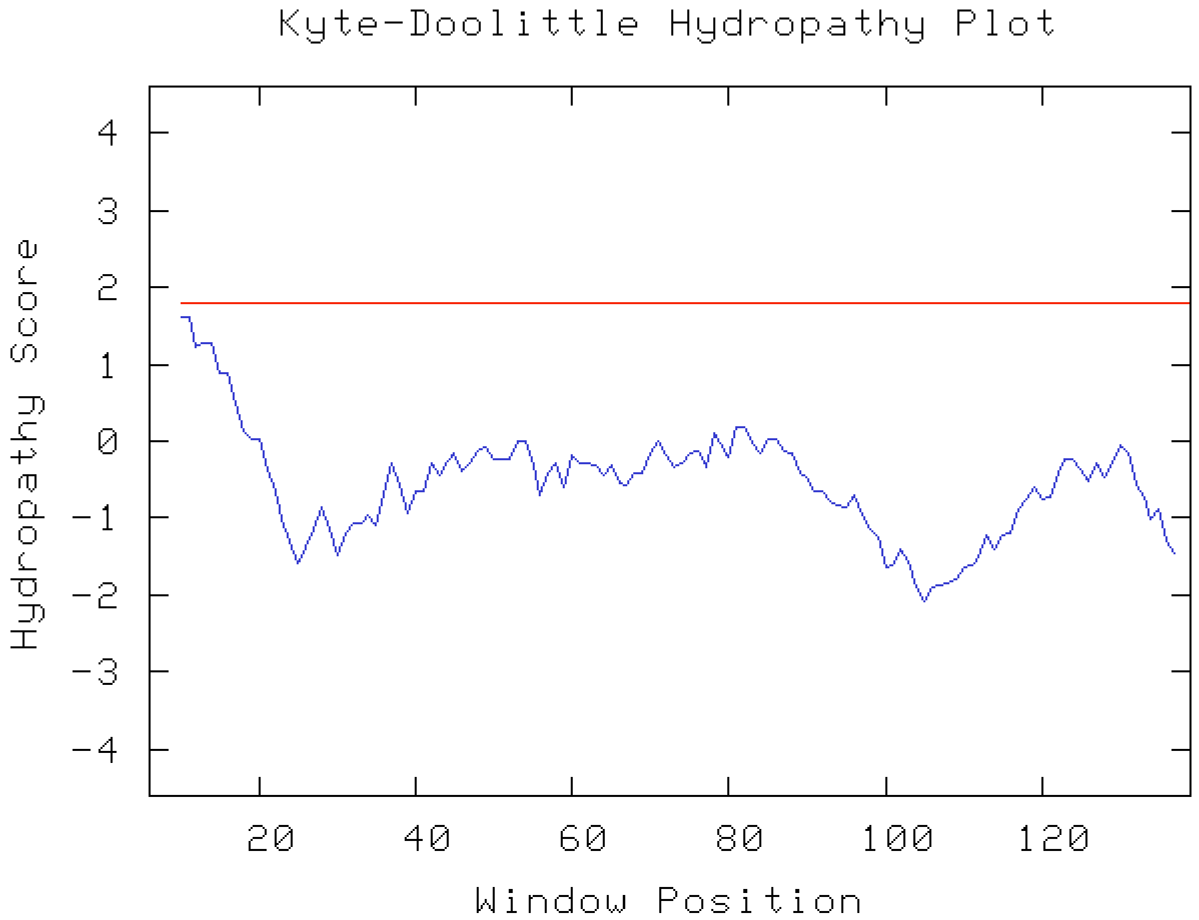

Kyte-Doolittle Hydropathy Plot
Hydropathy Score
4
3
2
1
0
-1
-2
-3
-4
20
40
60
80
100
120
Window Position

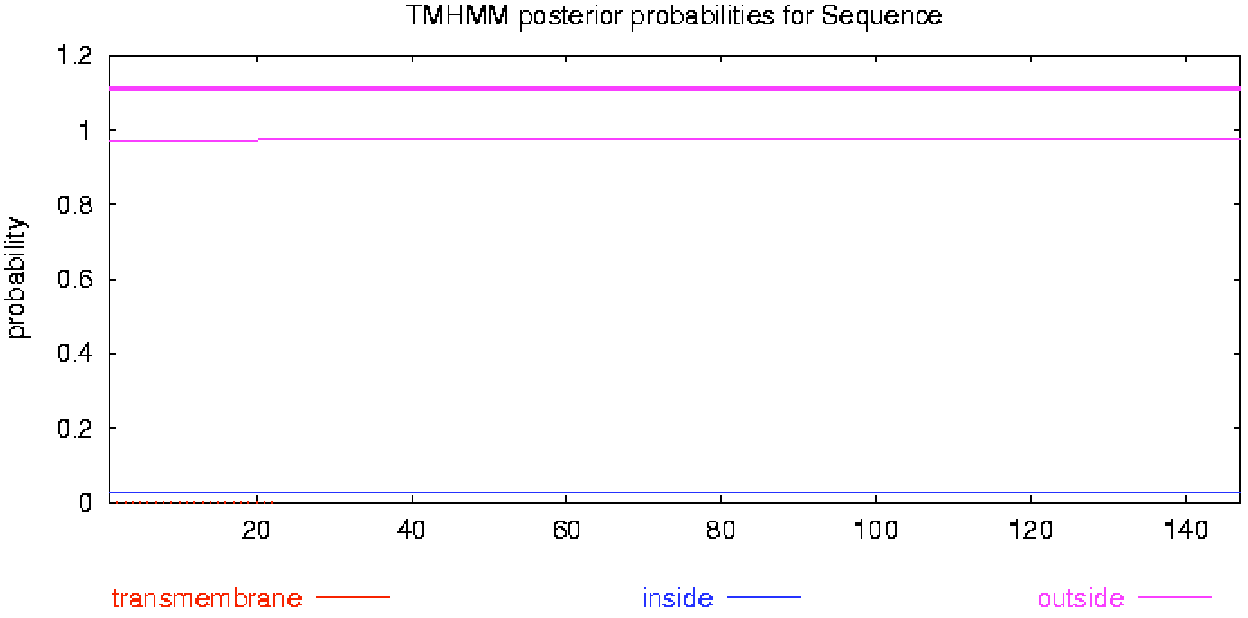

TMHMM posterior probabilities for Sequence
probability
1.2
1
0.8
0.6
0.4
0.2
0
20
40
60
80
100
120
140
transmembrane
inside
outside

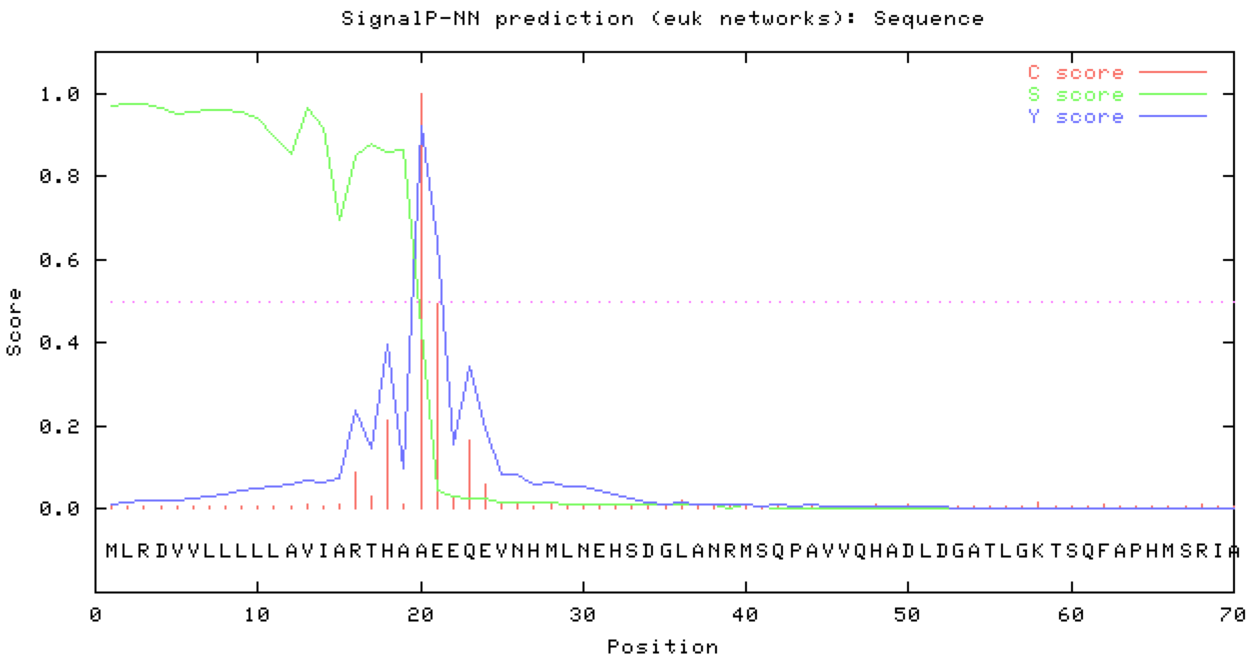

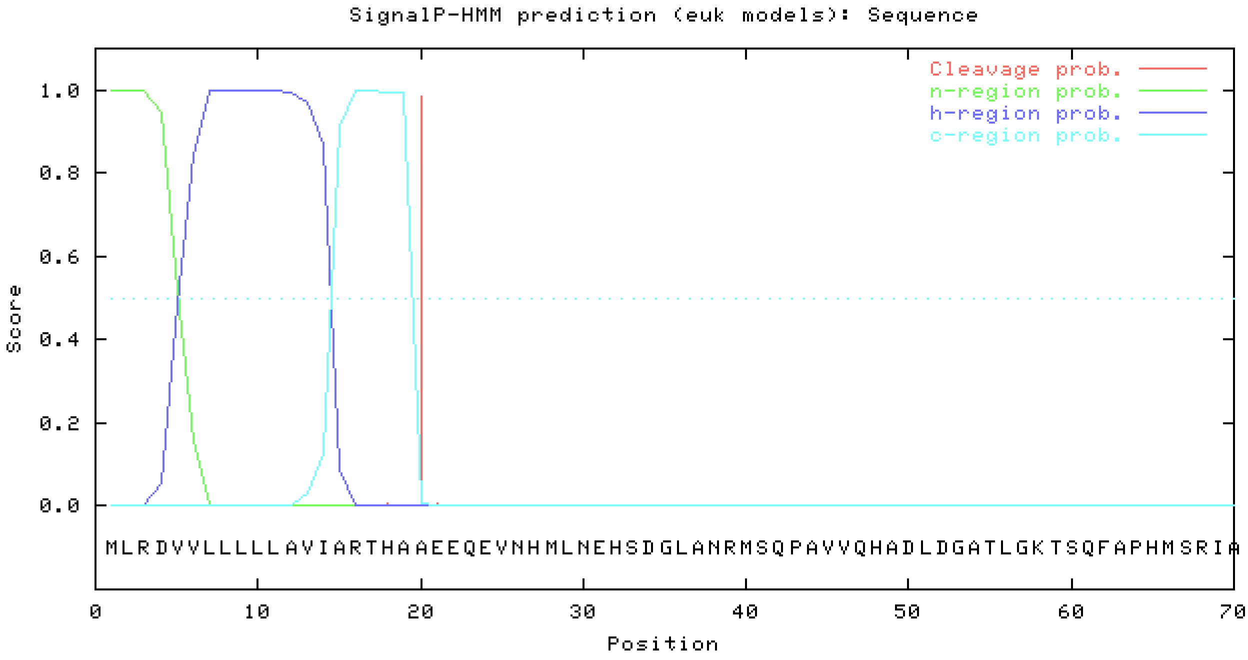

>dnaJ_signal_transit_sequence

MADHTVDLVDSLLQQAVLRNDRCSGNVLMVLSWLIGLCFAVLLSNPVLRITVIQEPTVALGLAPLRARFAIGSPTSAGQN
SQFQPGRRVSQLESESESATLWRTMPHRFHAGWPRPVIPRRRPSFPRADRNSEATCKTCPPPQ

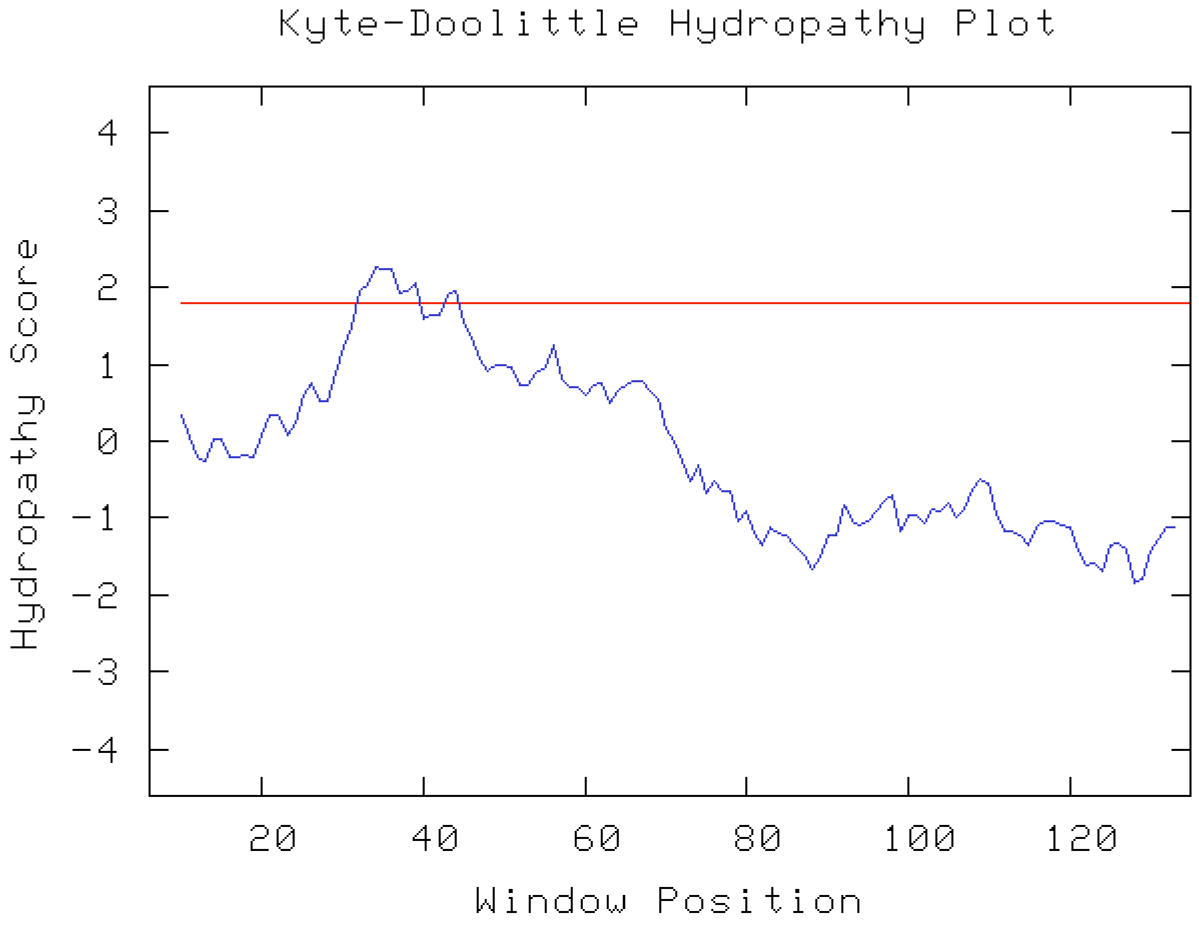

Kyte-Doolittle Hydropathy Plot
Hydropathy Score
4
3
2
1
0
-1
-2
-3
-4
20
40
60
80
100
120
Window Position

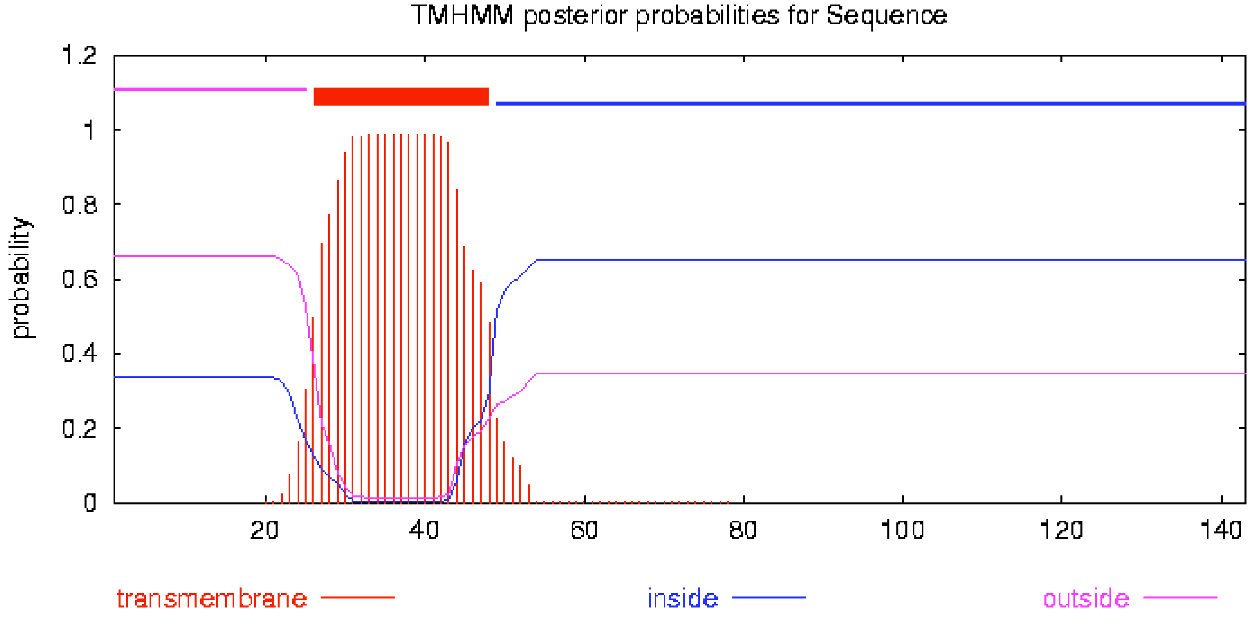

TMHMM posterior probabilities for Sequence
probability
1.2
1
0.8
0.6
0.4
0.2
0
20
40
60
80
100
120
140
transmembrane
inside
outside

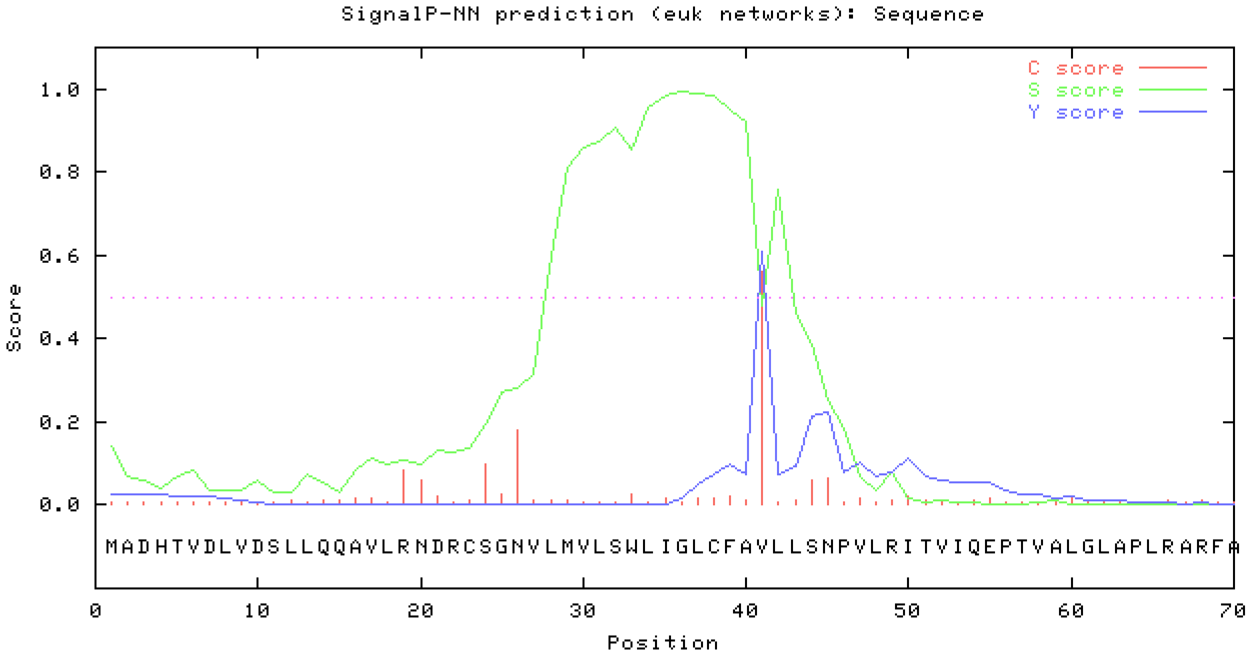

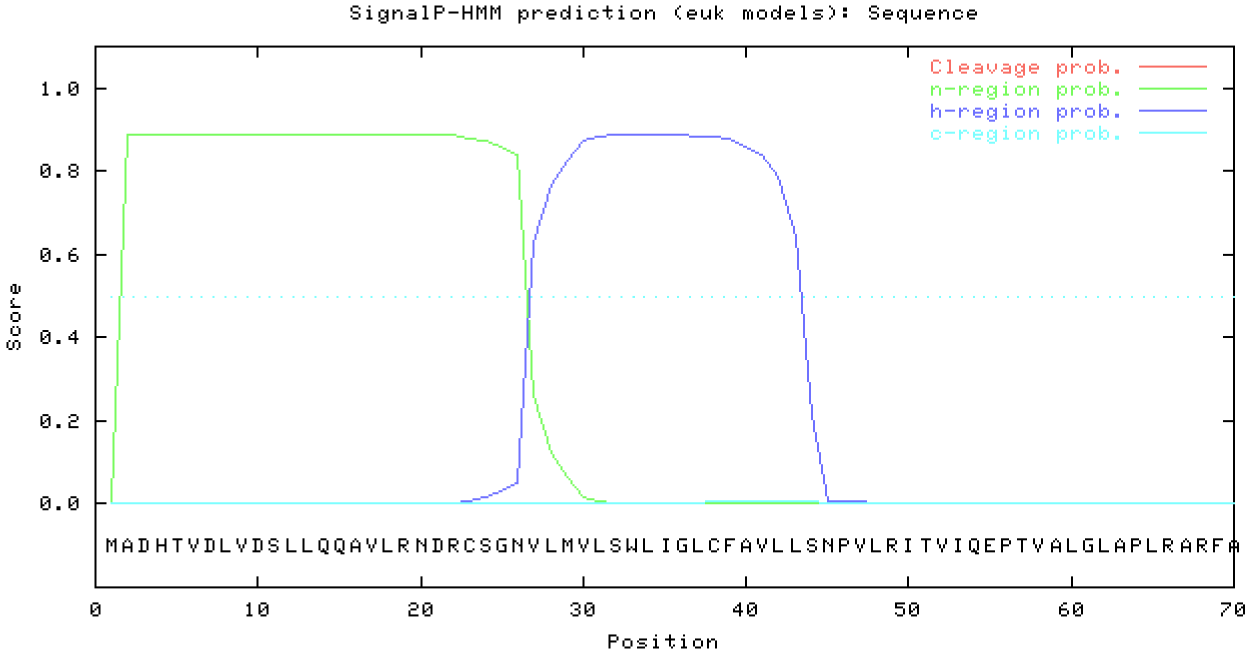

After N-27aa cut

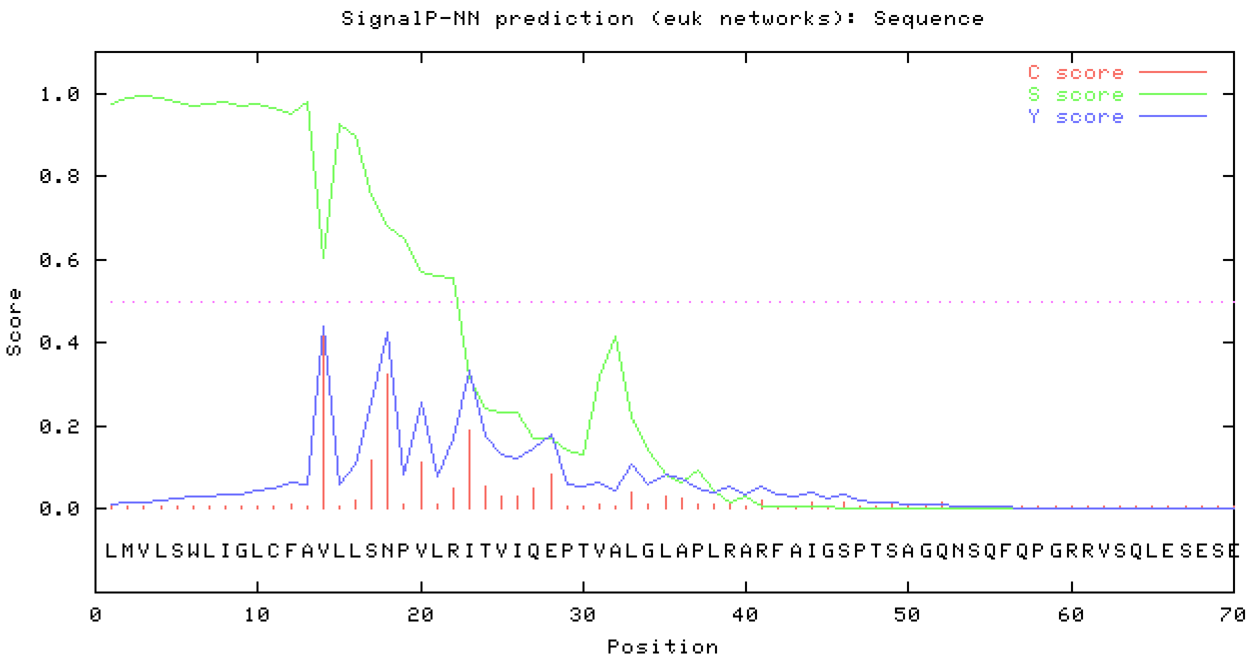

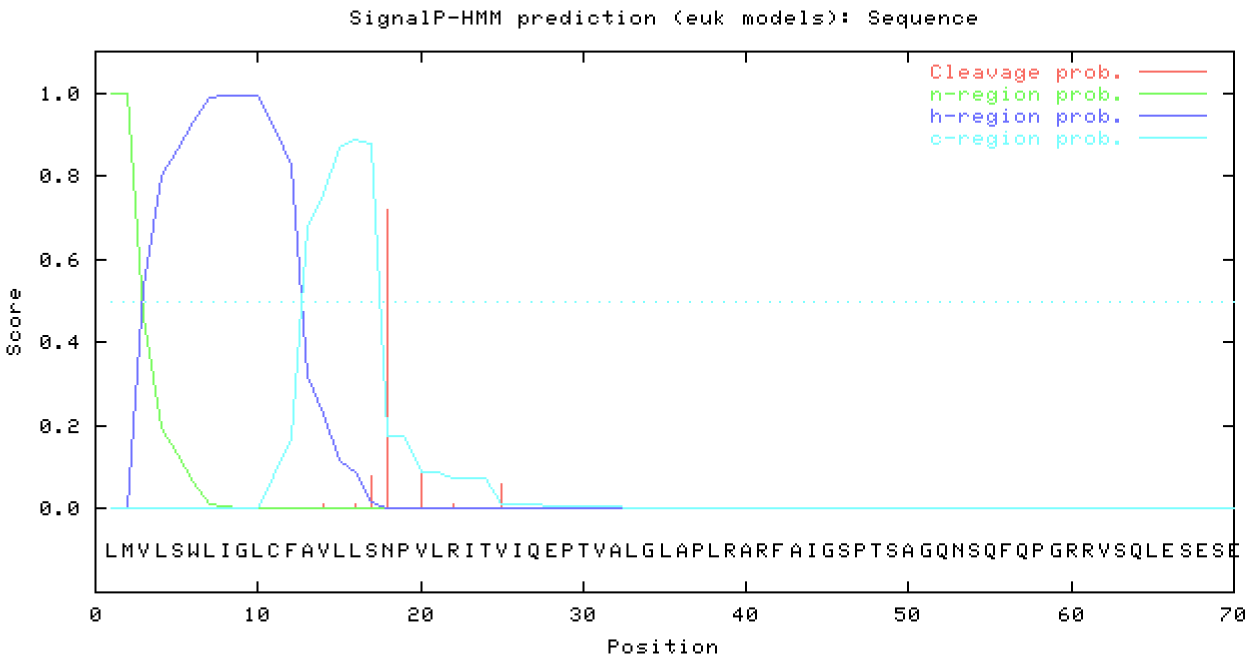

>dvr1_signal_transit_sequence

MQRVVIVTLLASIARTSG*TELLNVQPGTLTIPRSINHYKSEQRRAYKMPTNSRTKAIREQHGAPDRNWPSDAPKATWA
LMGNRQNRIAAAKAIAADGLSTA

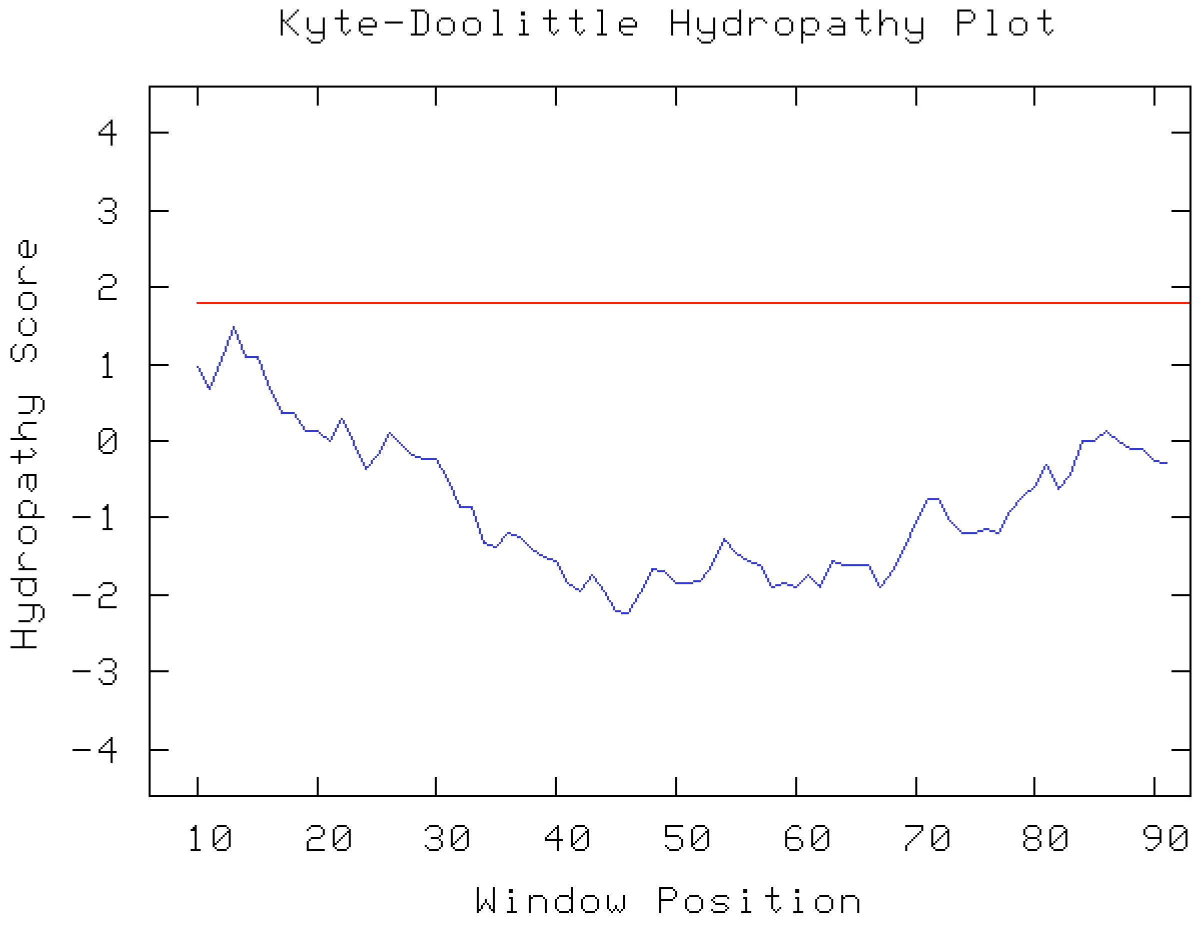
Kyte-Doolittle Hydropathy Plot
Hydropathy Score
Window Position
4
3
2
1
0
-1
-2
-3
-4
10
20
30
40
50
60
70
80
90

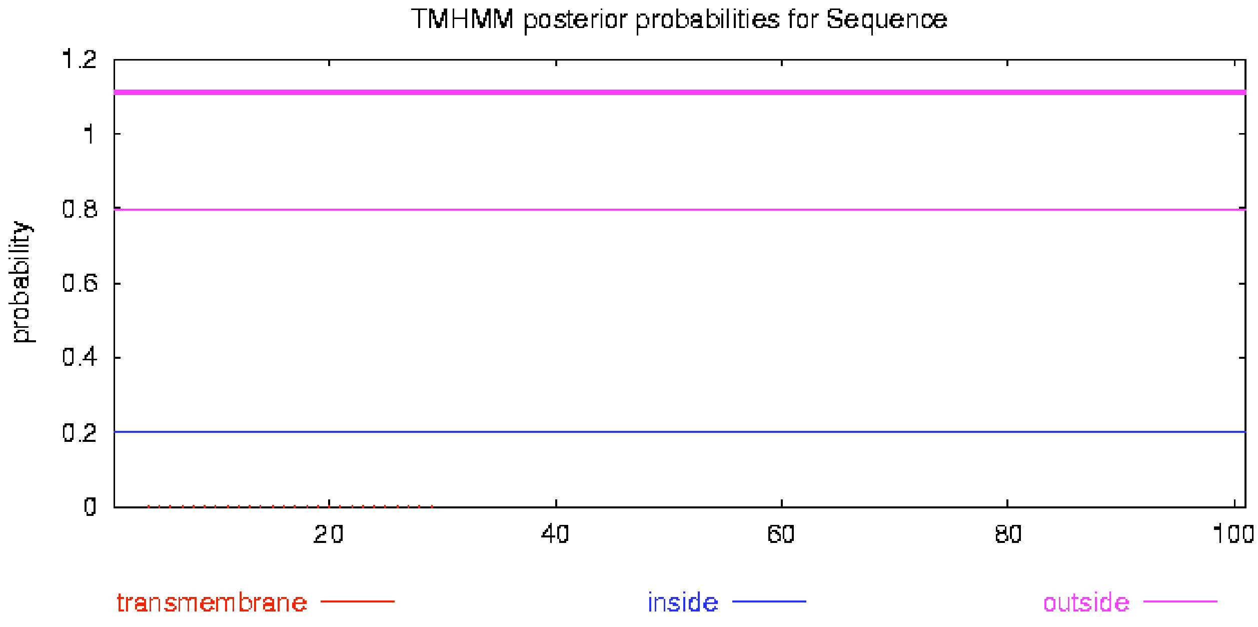
TMHMM posterior probabilities for Sequence
probability
1.2
1
0.8
0.6
0.4
0.2
0
20
40
60
80
100
transmembrane
inside
outside

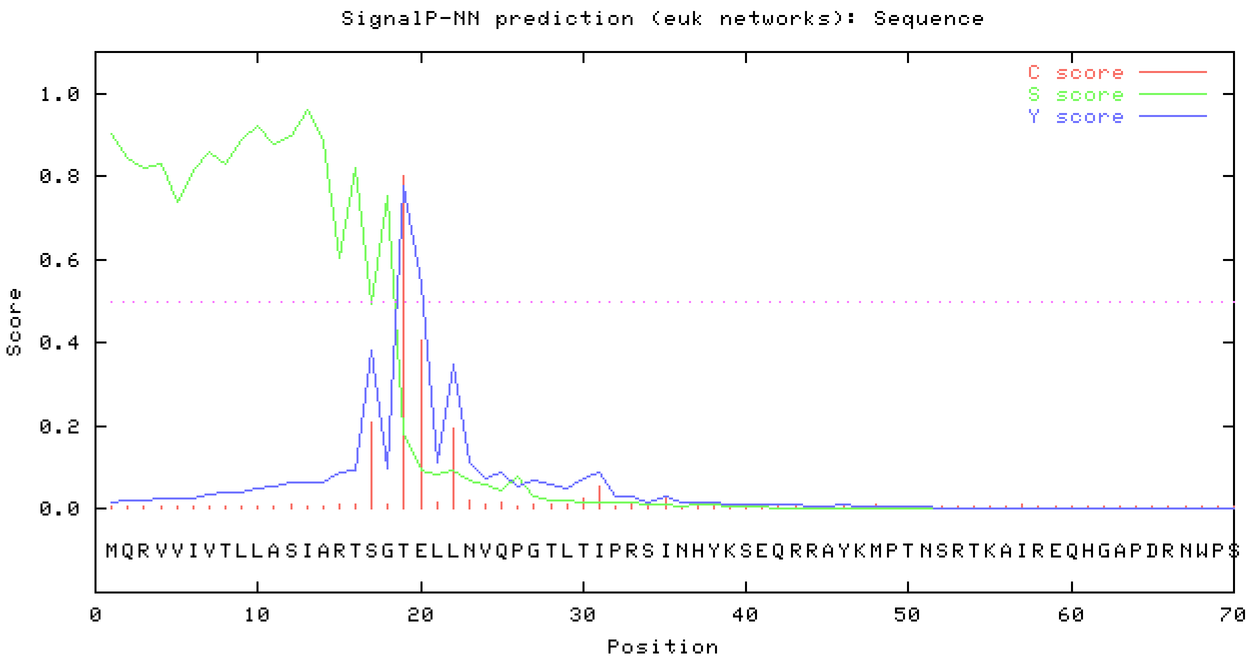

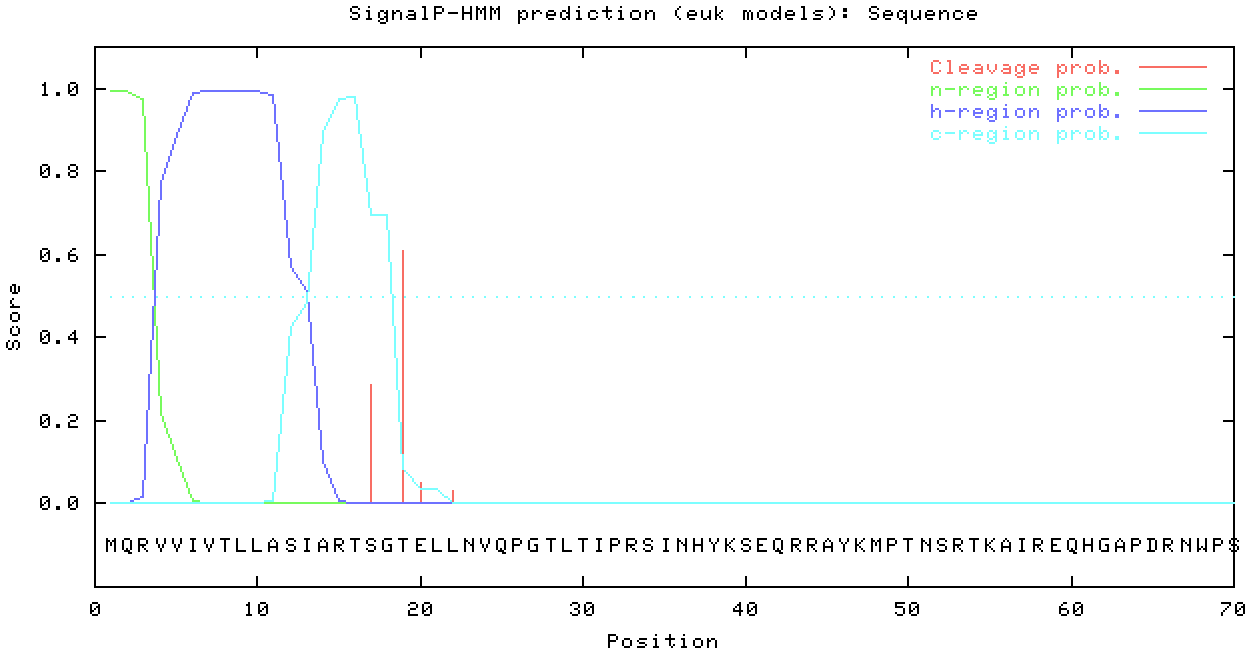

>dxr_signal_transit_sequence

MADDYIYYQIAPGGGVDSQPASVKRRFPIGITLIVGFSVALVCFLAASVSPWEYRAIAAASTNLIGSPFASKMGKLSNKP
QQGLTLSLSSGRSVTPVASWPSVWSKLRGVAKERHHNPVYQCRAAQSSWESGRNLVATNTARDSSELPVK

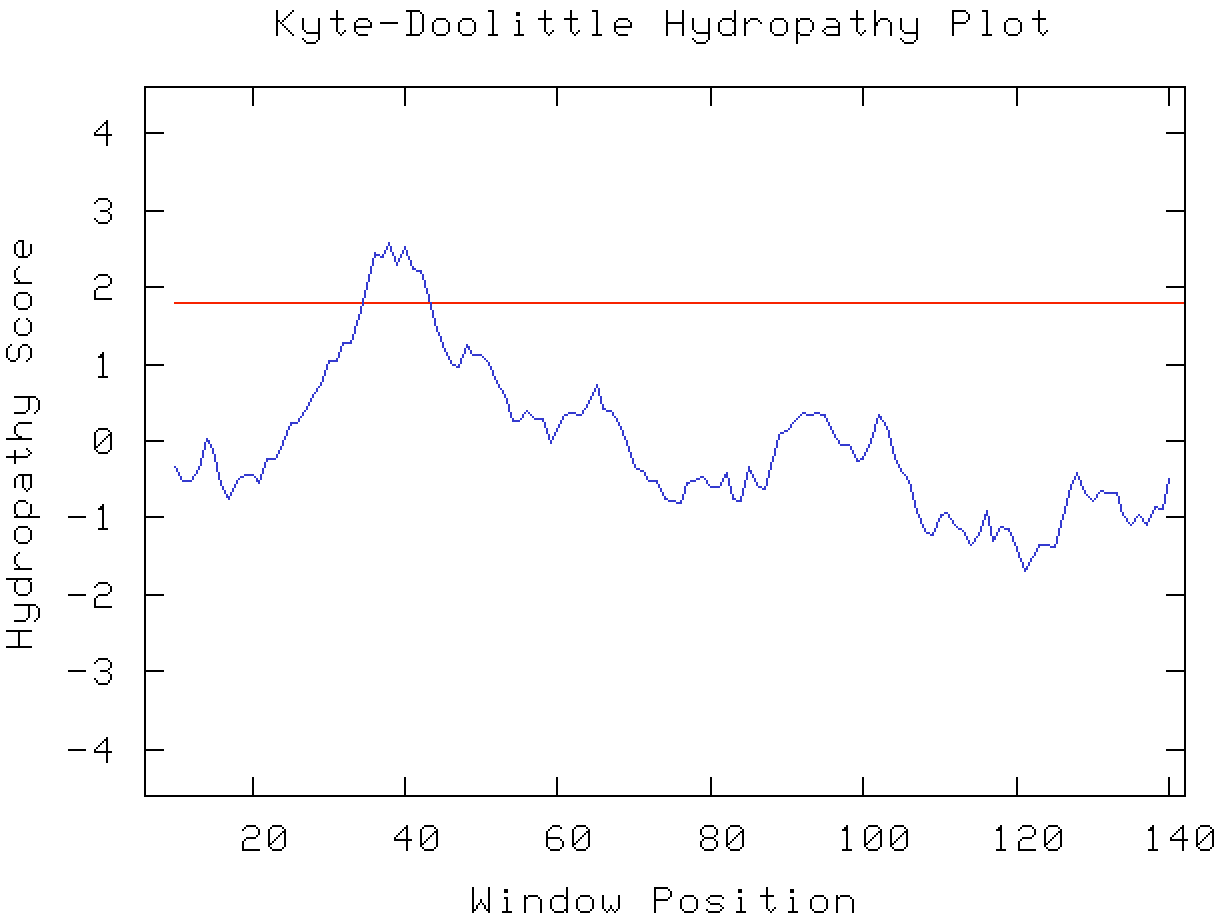

Kyte-Doolittle Hydropathy Plot
Hydropathy Score
Window Position
4
3
2
1
0
-1
-2
-3
-4
20
40
60
80
100
120
140

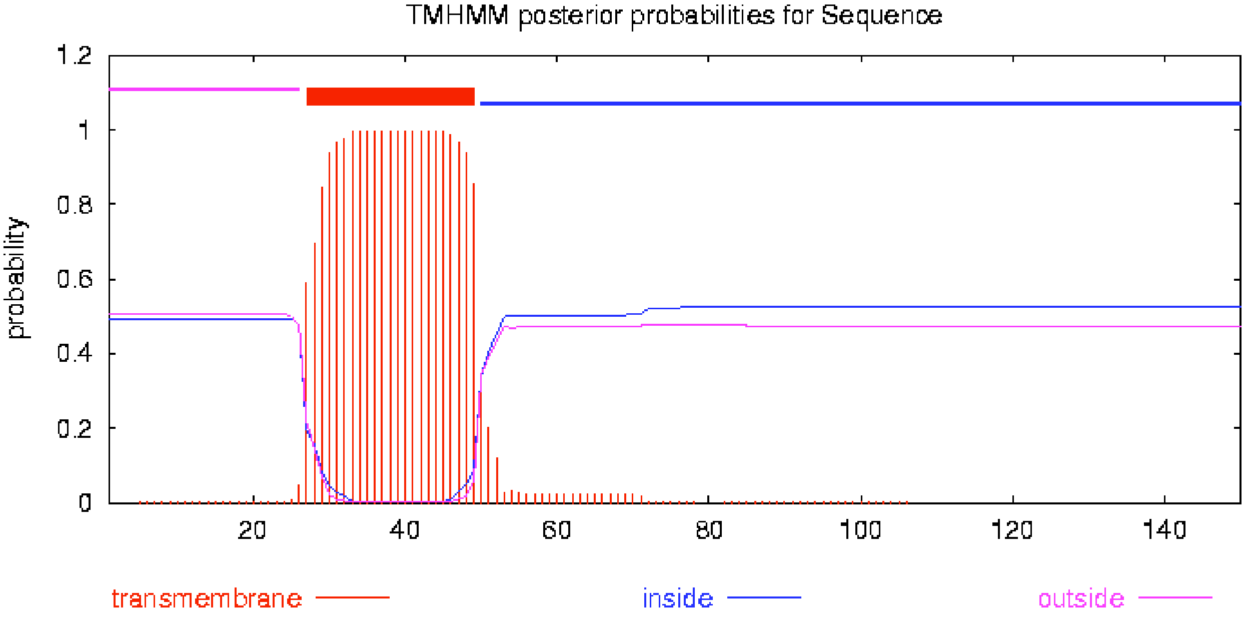

TMHMM posterior probabilities for Sequence
probability
1.2
1
0.8
0.6
0.4
0.2
0
20
40
60
80
100
120
140
transmembrane
inside
outside

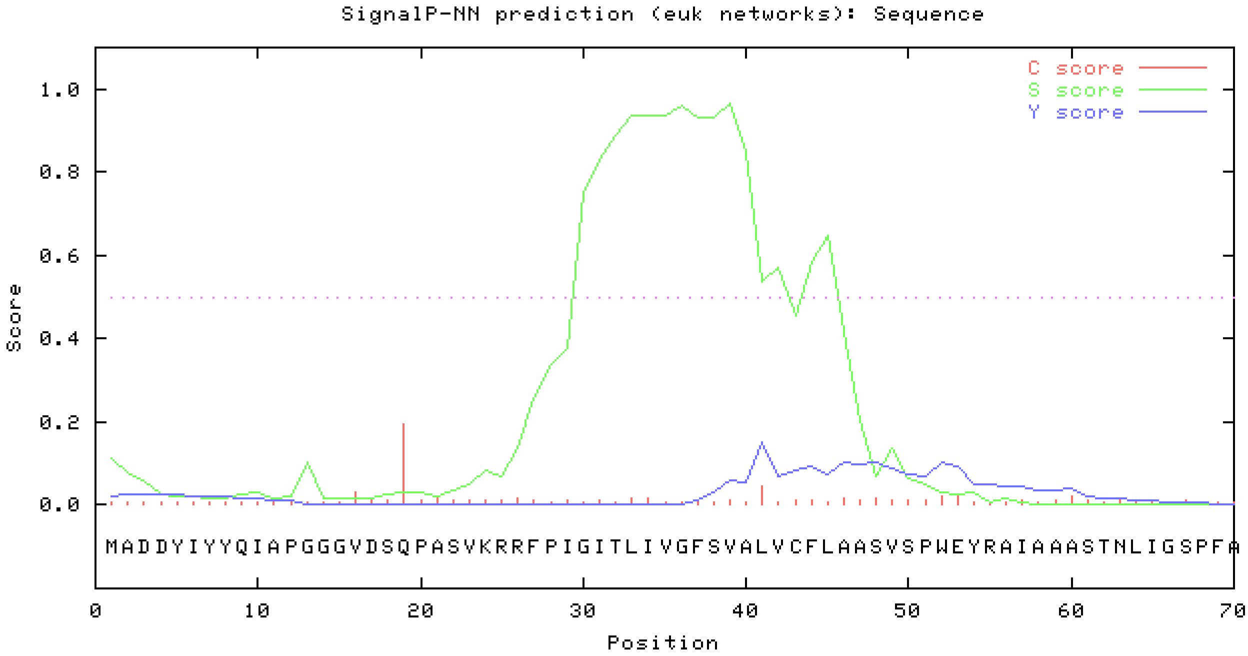

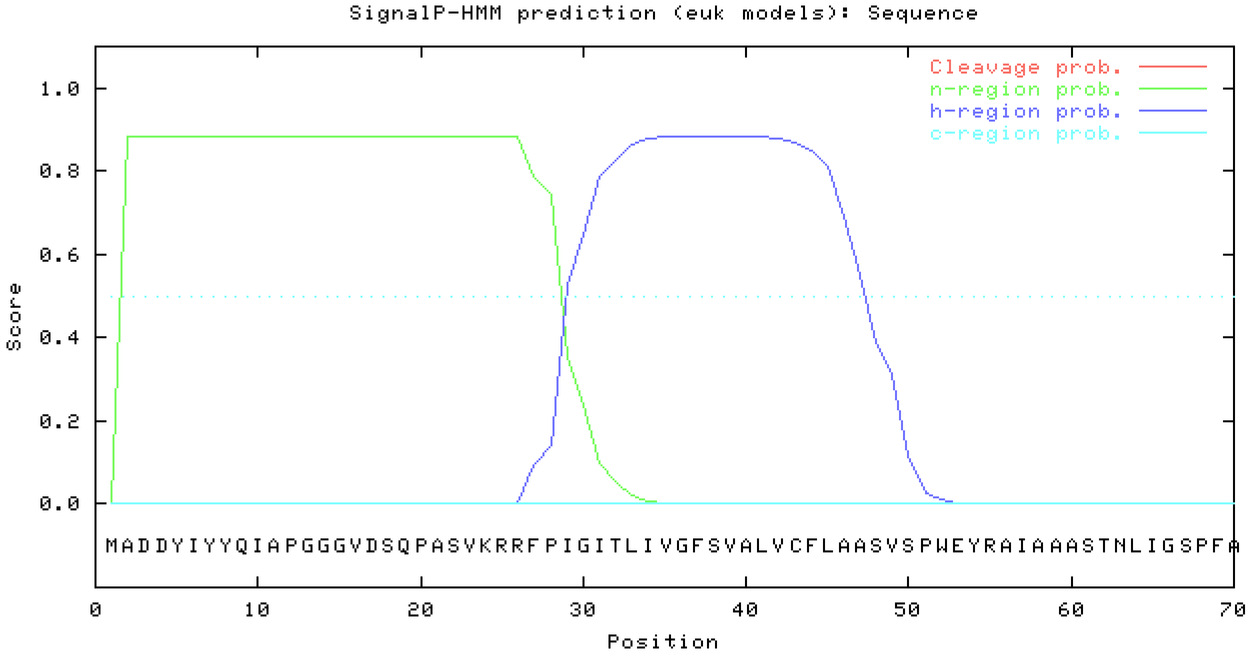

After N-27aa cut

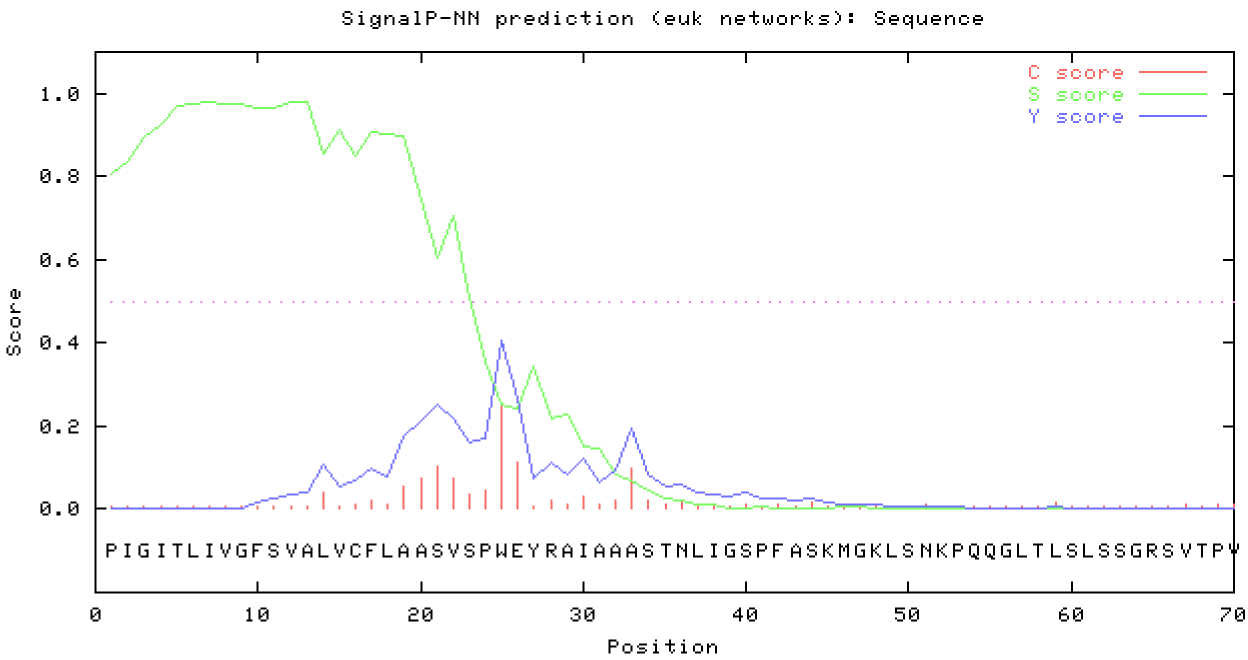

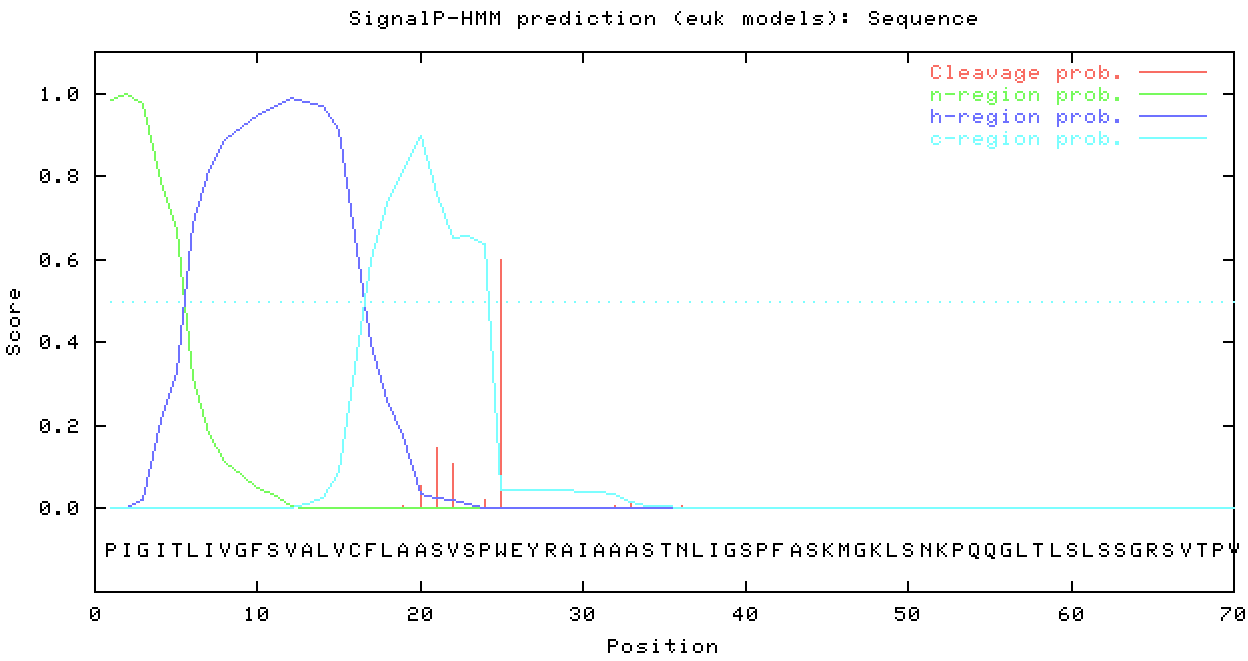

>fbp_signal_transit_sequence

MASPSTVLAQPLTQVEDASRGARNNVKVIAFSLFFGFGCAVLWNQVISAAARQHSVVQEPIVMSALTQSTGAWQPLRQTM

EHAKAGQSIQAPRWPHQPKNWNPMDWSQMPPRH

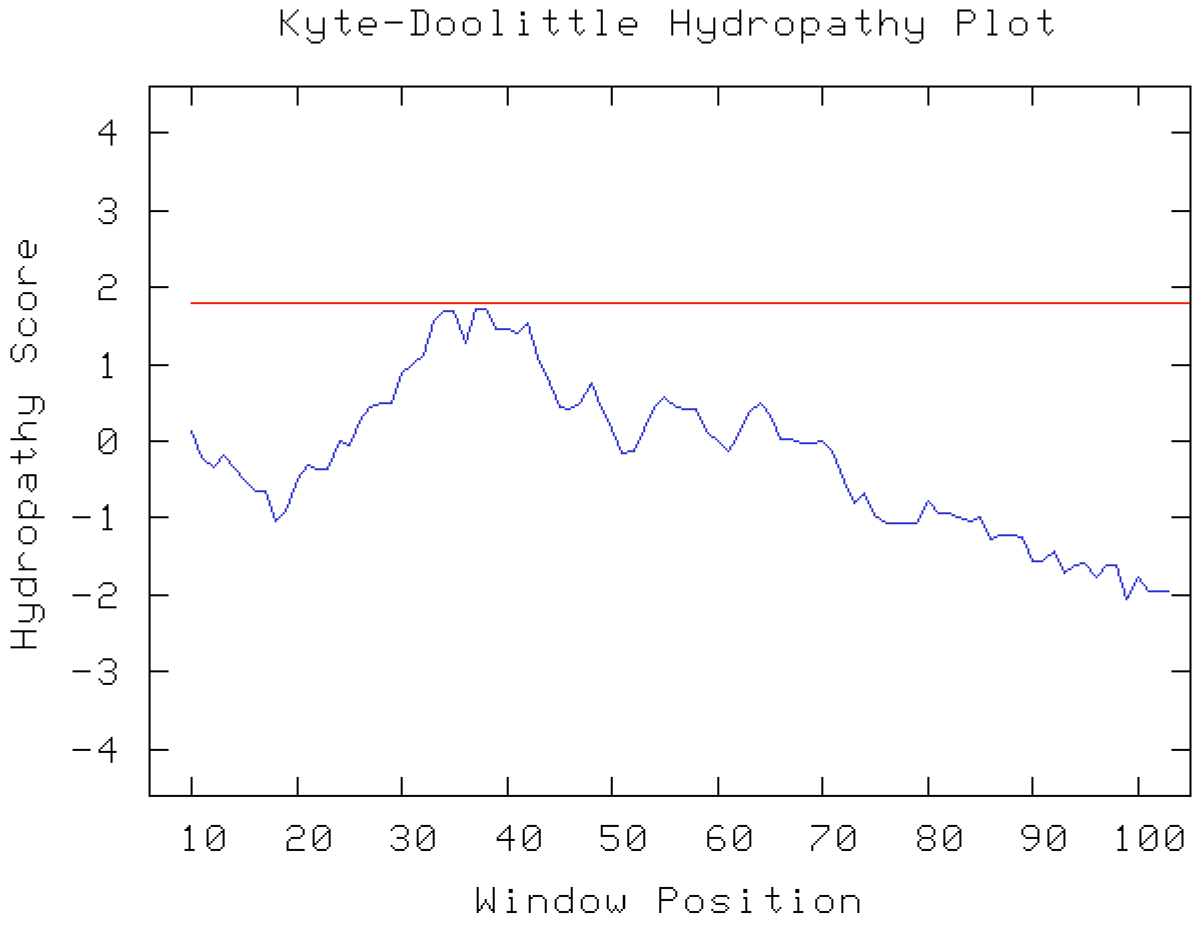
Kyte-Doolittle Hydropathy Plot
Hydropathy Score
4
3
2
1
0
-1
-2
-3
-4
10
20
30
40
50
60
70
80
90
100
Window Position

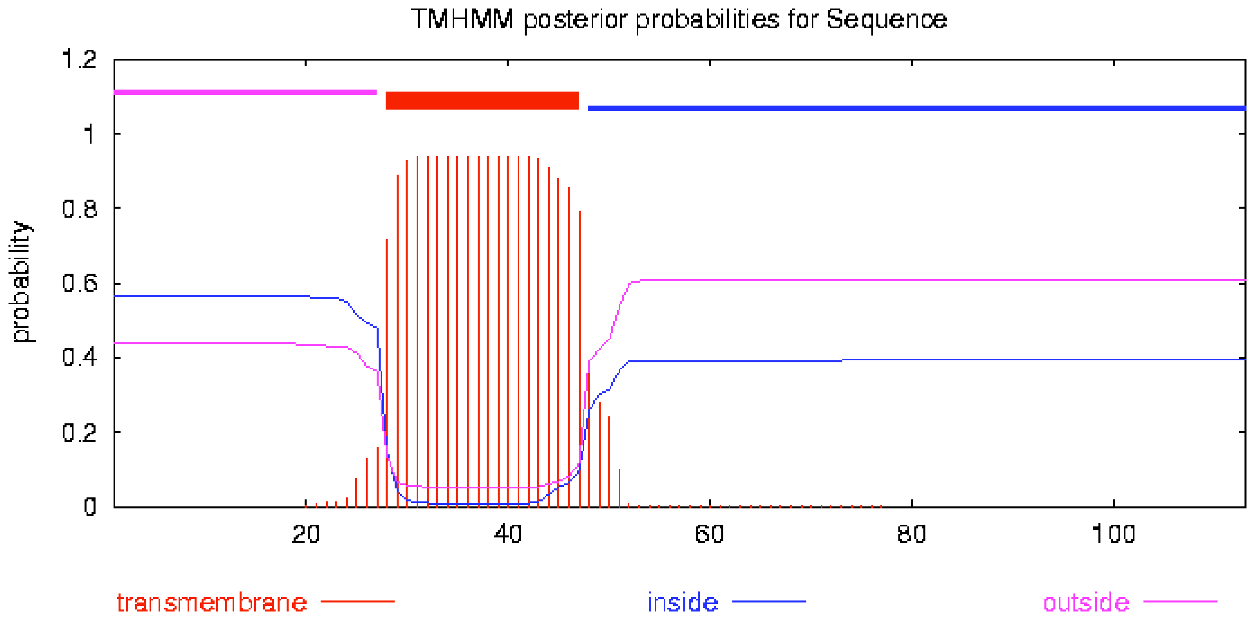
TMHMM posterior probabilities for Sequence
probability
1.2
1
0.8
0.6
0.4
0.2
0
20
40
60
80
100
transmembrane
inside
outside

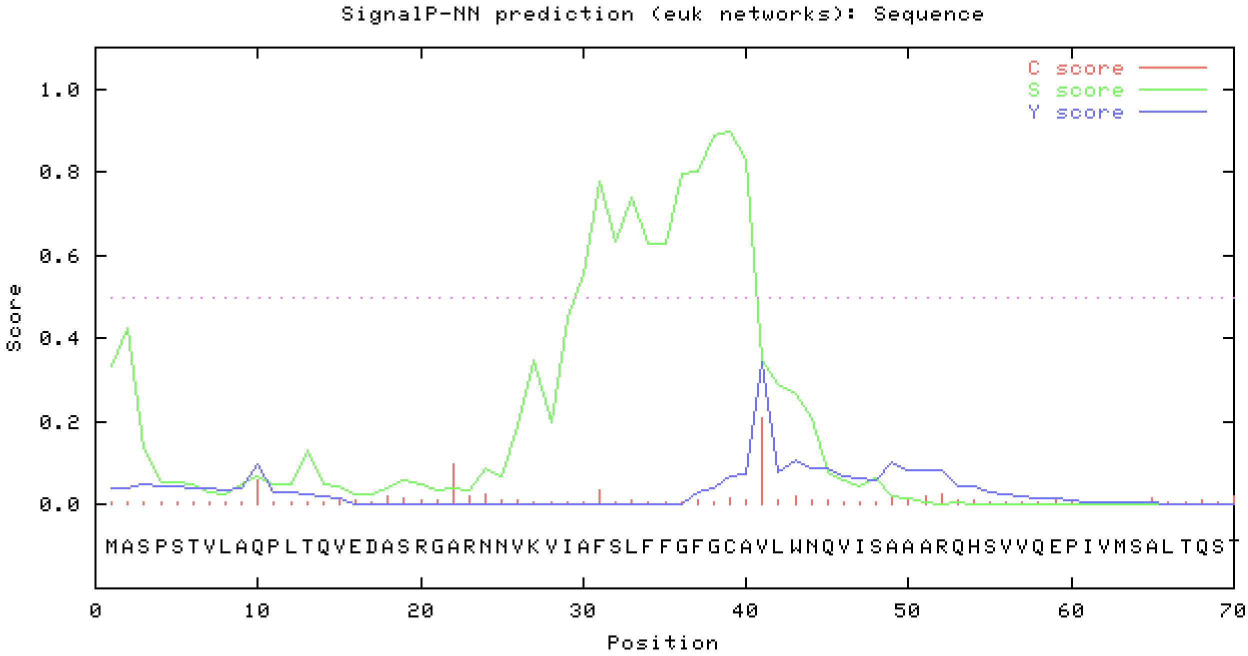

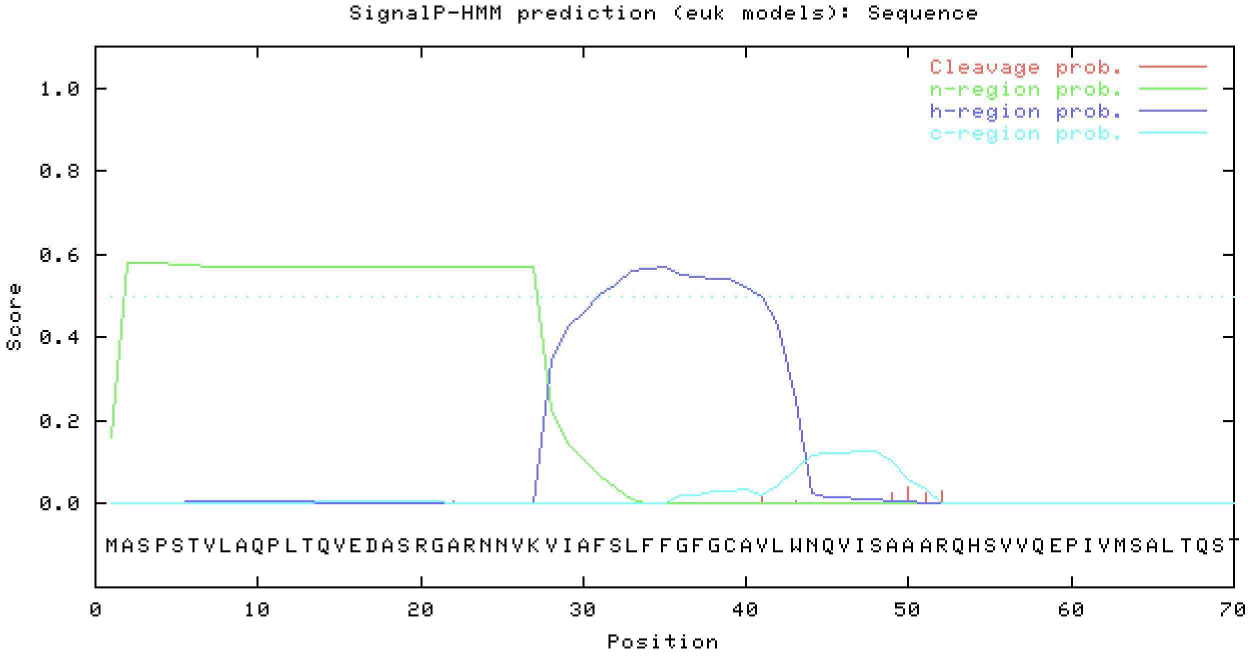

After N-28aa cut

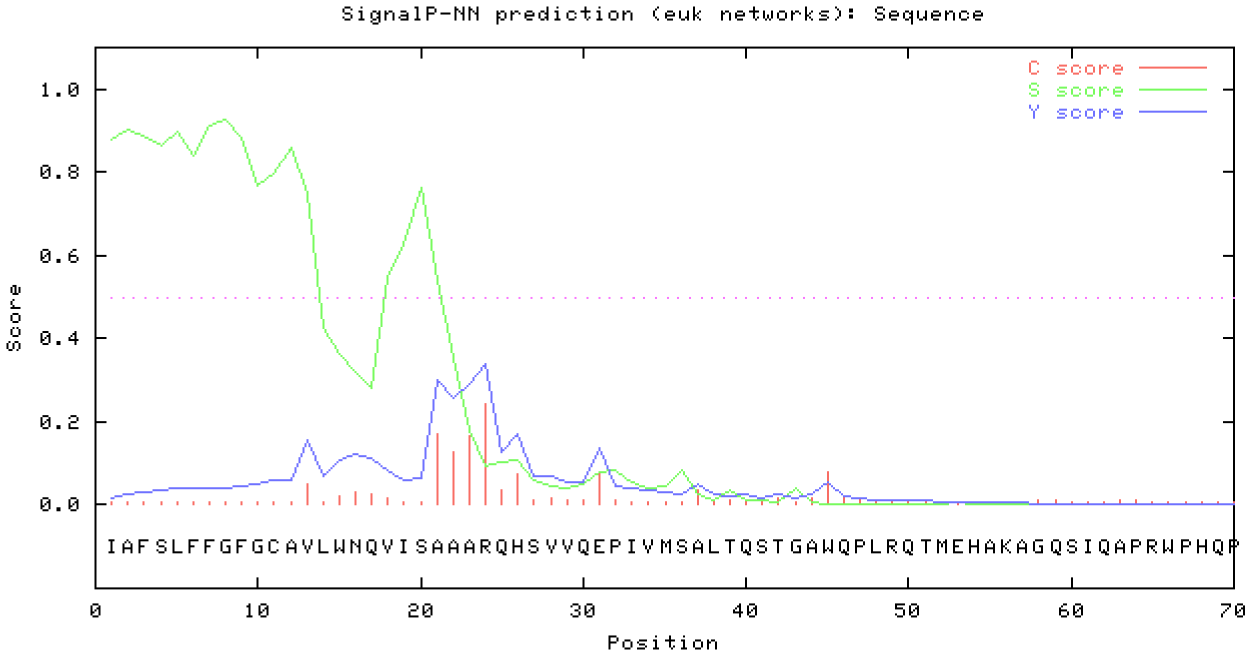

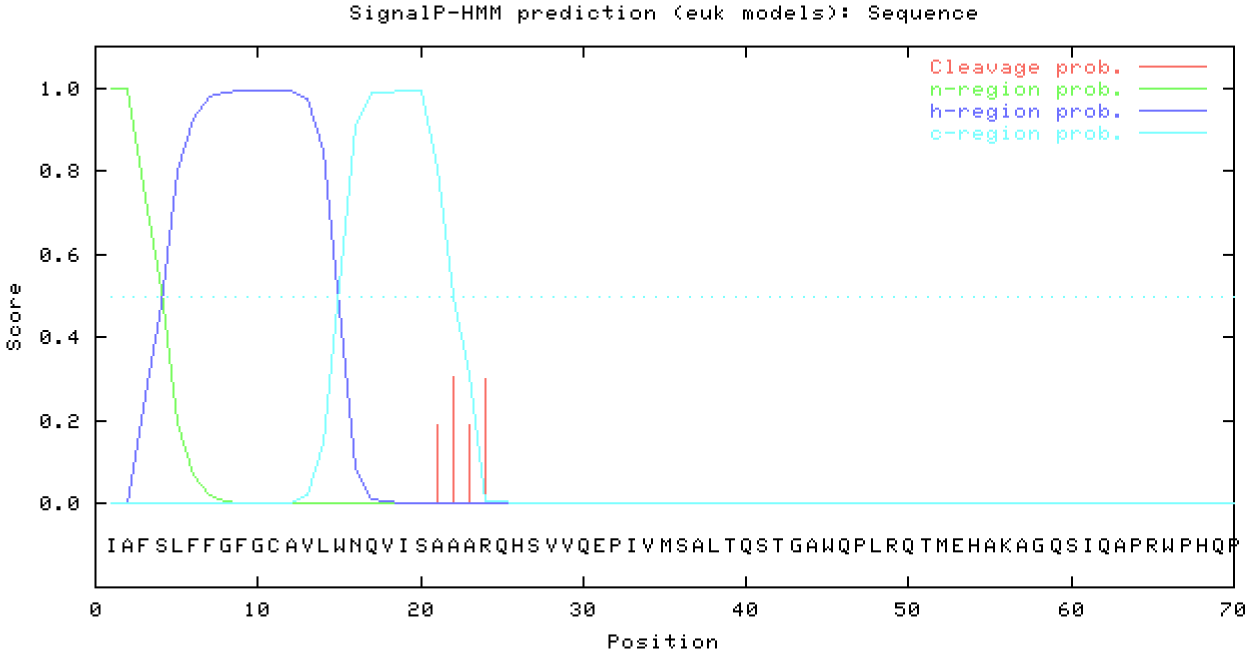

>fer2_signal_transit_sequence

MSSATVALSPKGNLFMLFGLLLGGTALLSLSLKYSRREEPAALWANAQGAFARMSPVRAKPWMASPGLRRPLNSPRMIPR

DLKVR

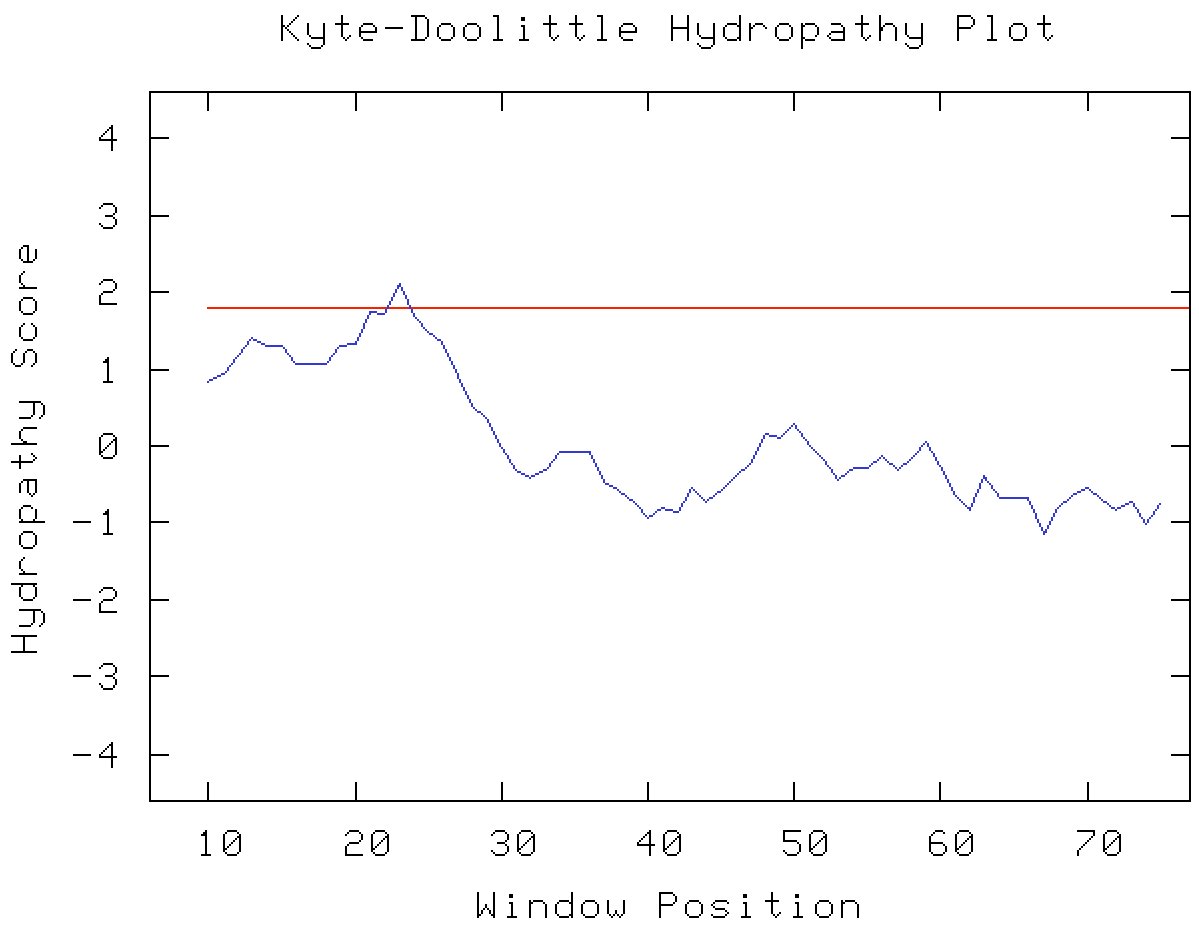
Kyte-Doolittle Hydropathy Plot
Hydropathy Score
4
3
2
1
0
-1
-2
-3
-4
10
20
30
40
50
60
70
Window Position

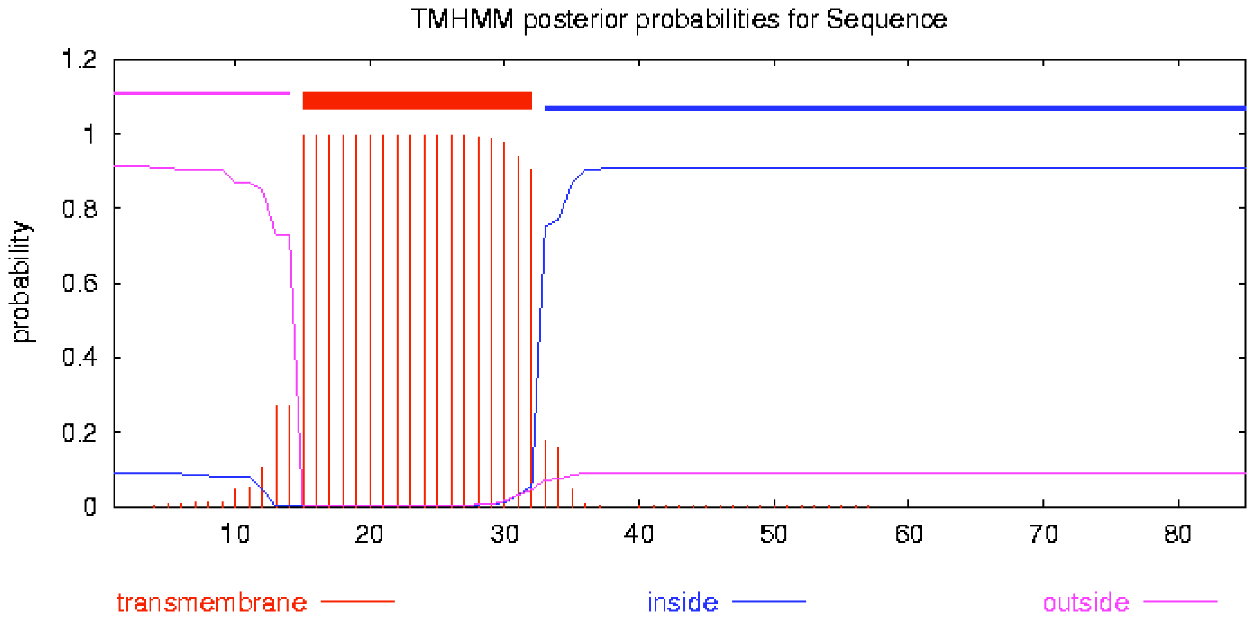
TMHMM posterior probabilities for Sequence
probability
1.2
1
0.8
0.6
0.4
0.2
0
10
20
30
40
50
60
70
80
transmembrane
inside
outside

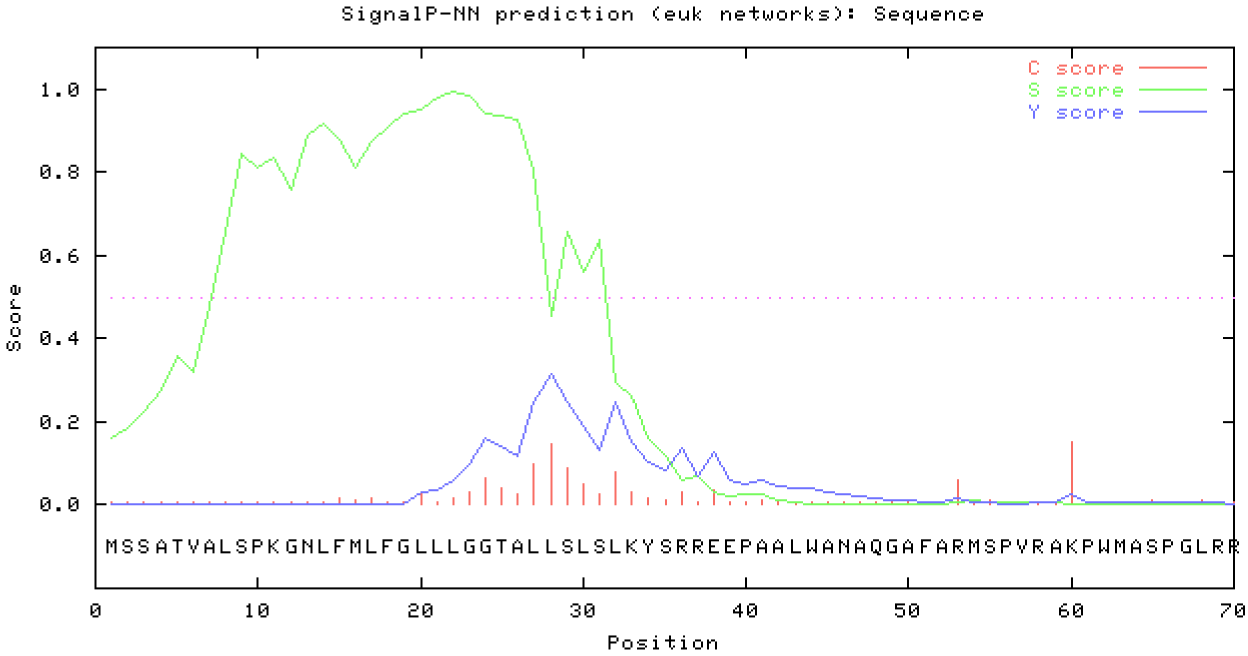

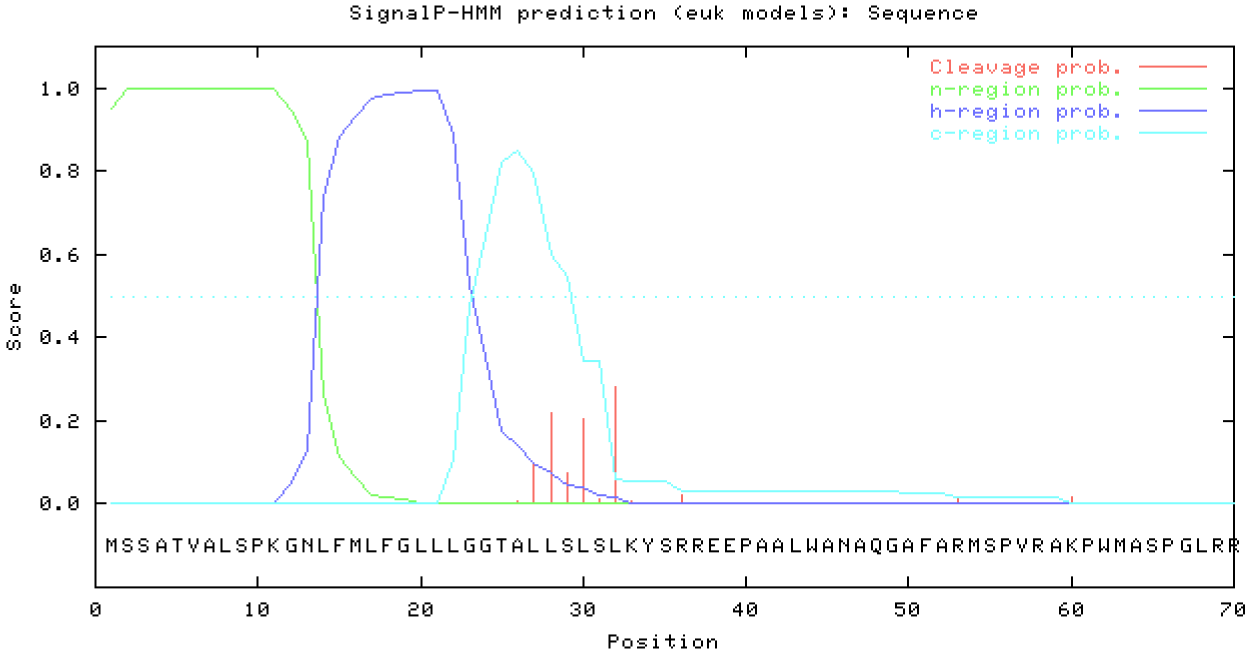

After N-14aa cut

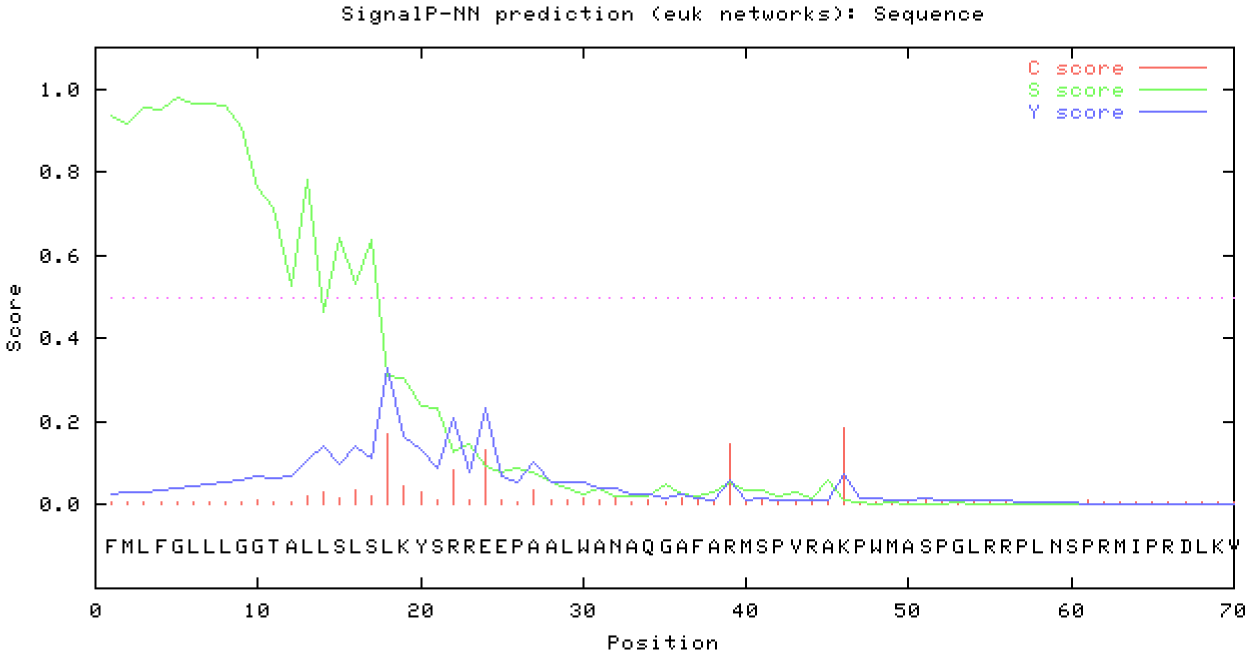

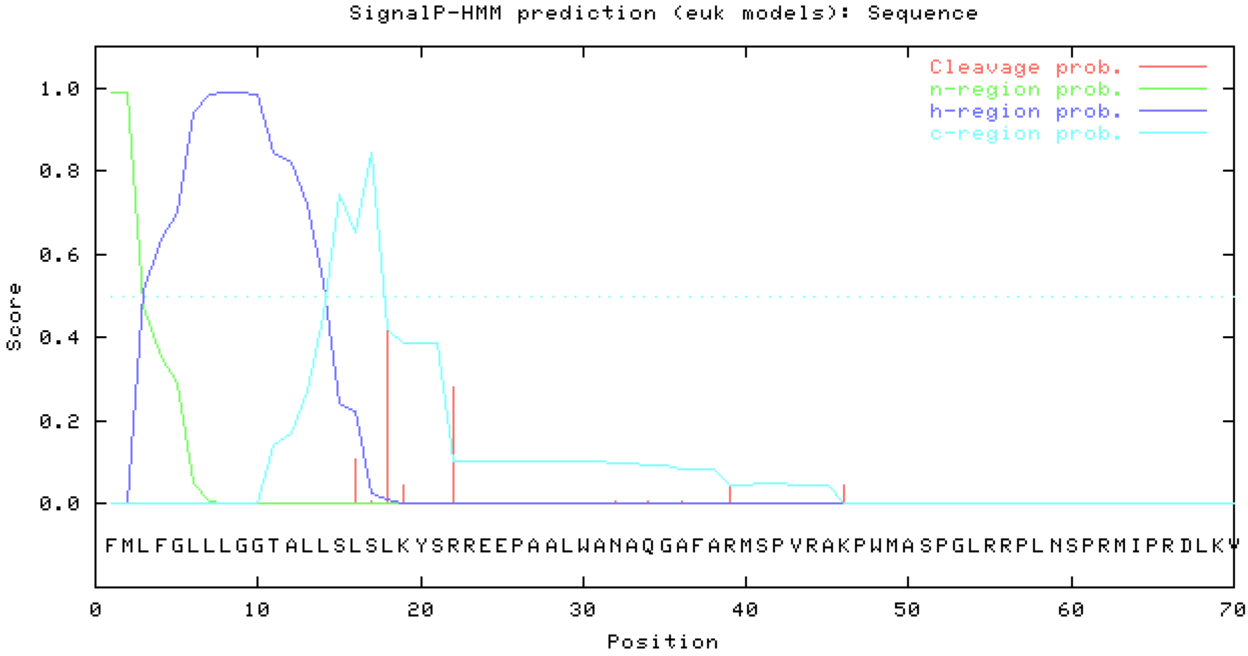

>fnr_signal_transit_sequence

MADQSFAPFEPMNQEEGKGRKKVLRSIASLLIGFGVGCALVAKAAMQPSAVDELAISEAWSPLQRSSATLGHRFMMPSAR

FPVGPVREAVNEKMYCRQAVILNAEVAAPPKKKGVKIVDGR

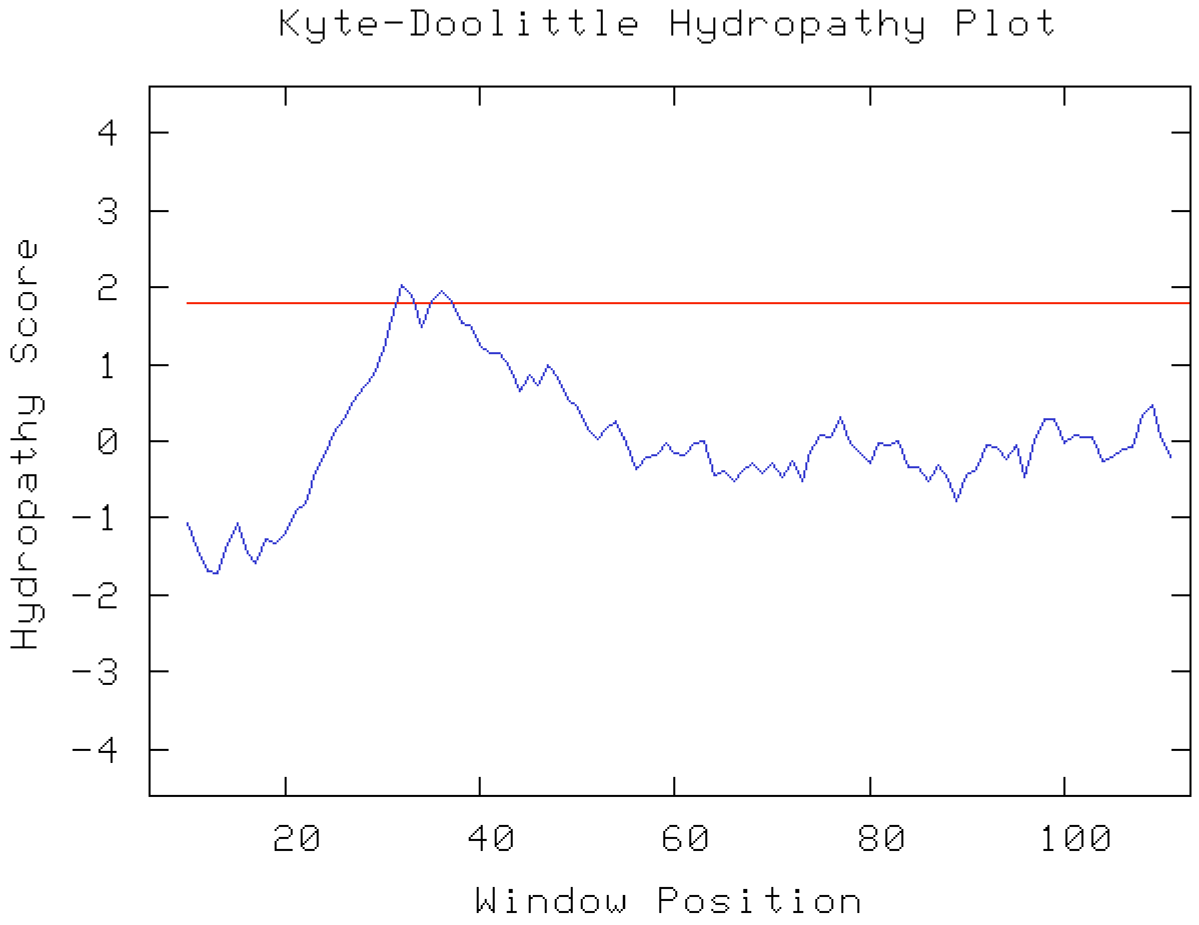

Kyte-Doolittle Hydropathy Plot
Hydropathy Score
4
3
2
1
0
-1
-2
-3
-4
20
40
60
80
100
Window Position

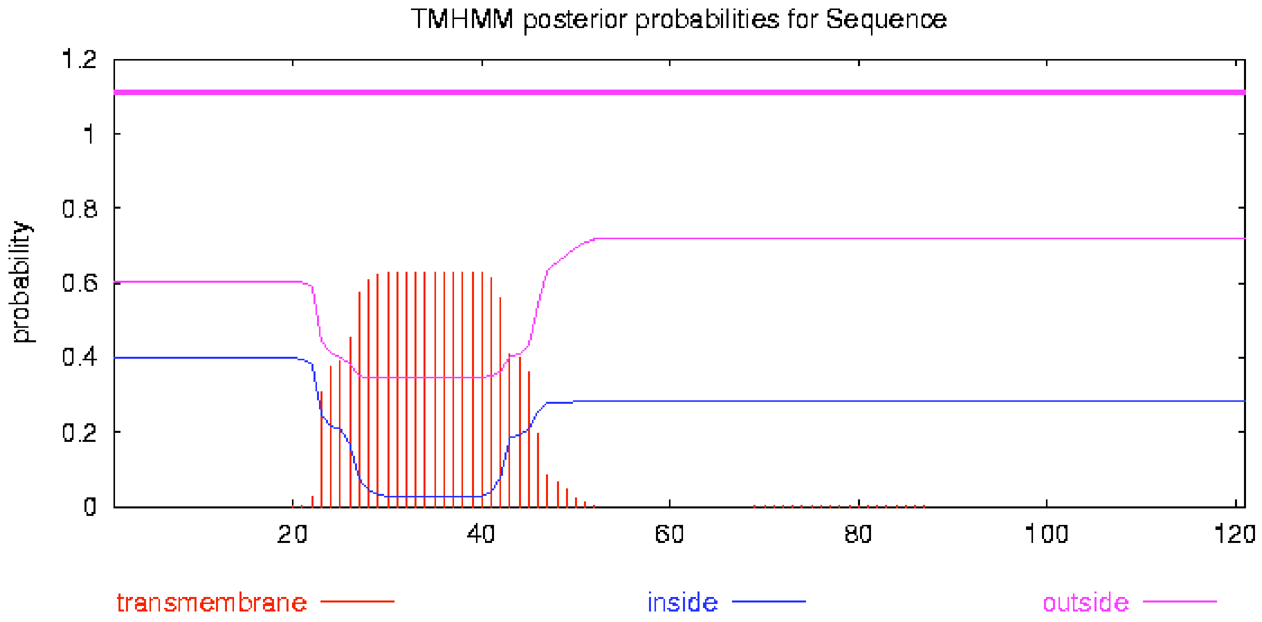

TMHMM posterior probabilities for Sequence
probability
1.2
1
0.8
0.6
0.4
0.2
0
20
40
60
80
100
120
transmembrane
inside
outside

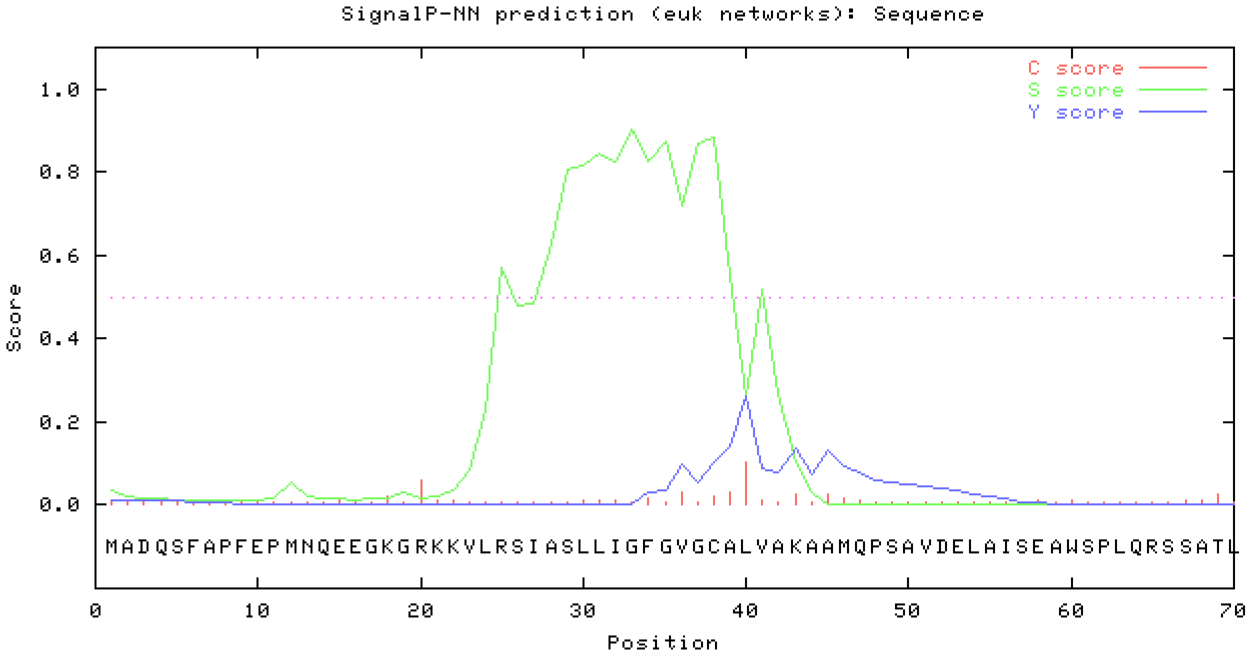

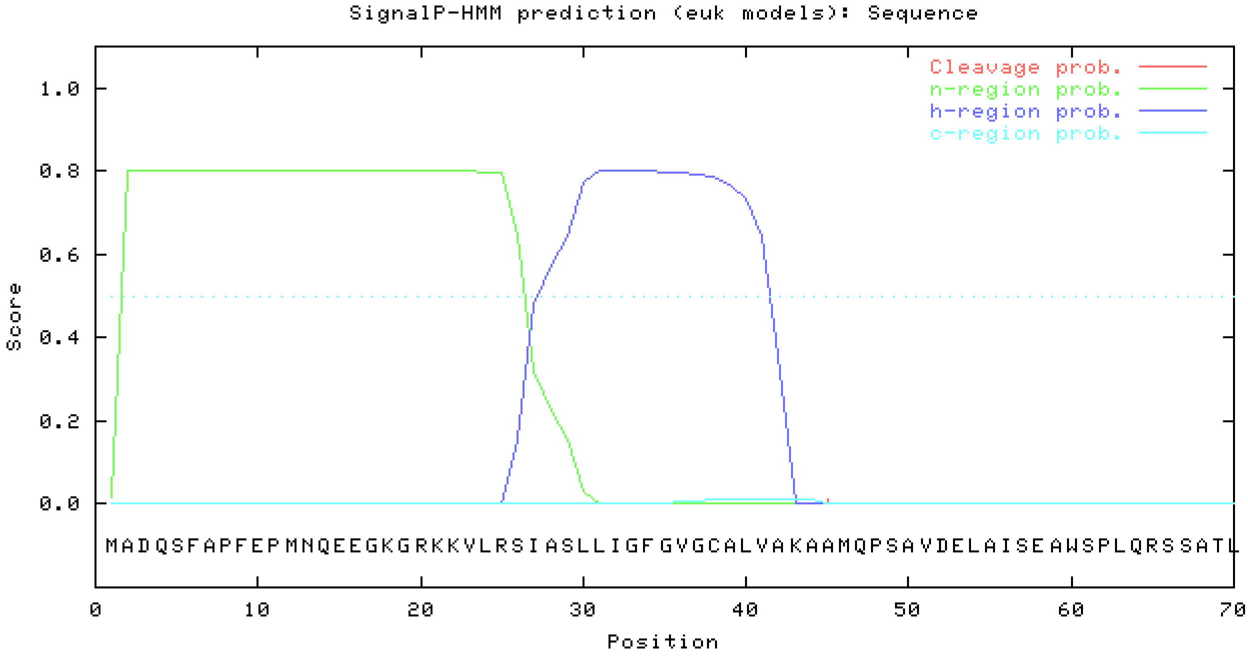

After N-27aa cut

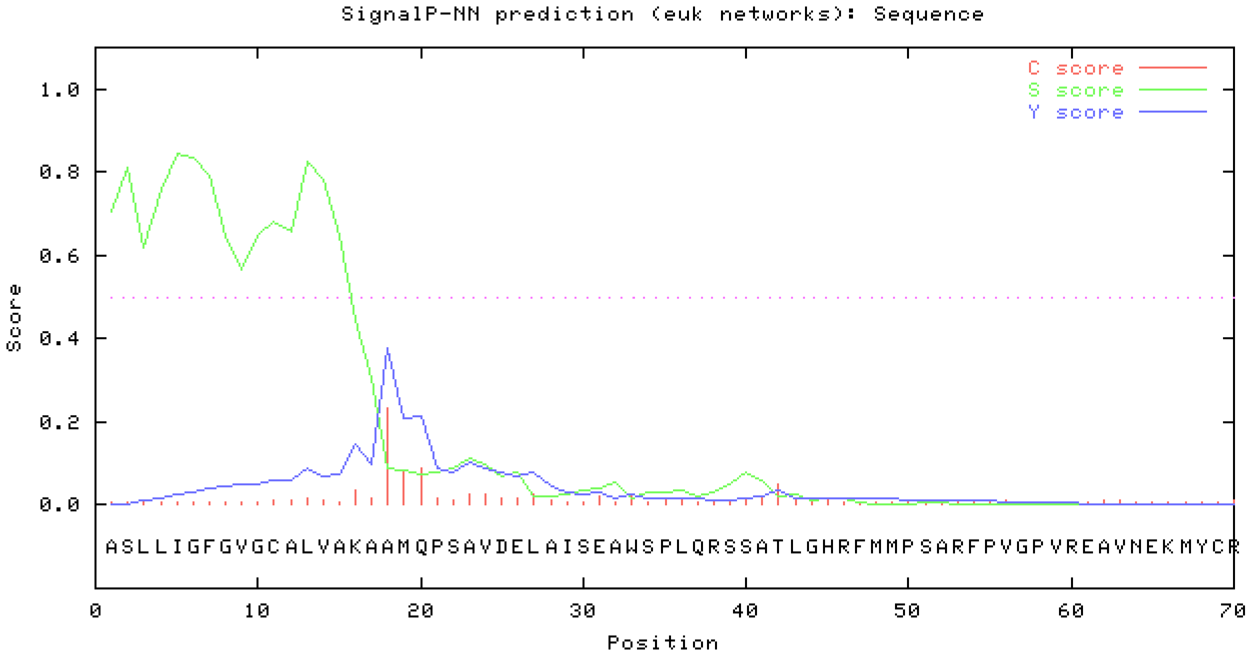

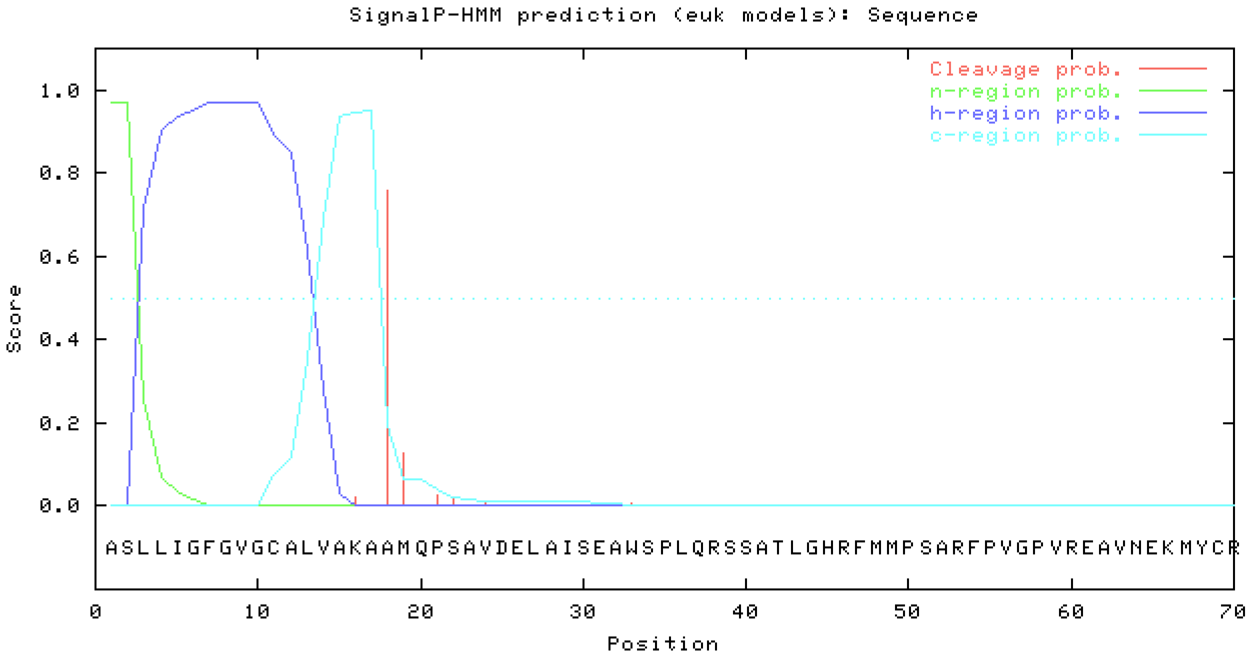

>gapdh_signal_transit_sequence

MAASYHPLGEQTVQEPGRRGTRVVQHVGALIIGFGMGCALIYASGGGQPLAVALPSESMAVRSKFMQPVMGSSPFGPEFA
ERRGPAPVRSTNPD

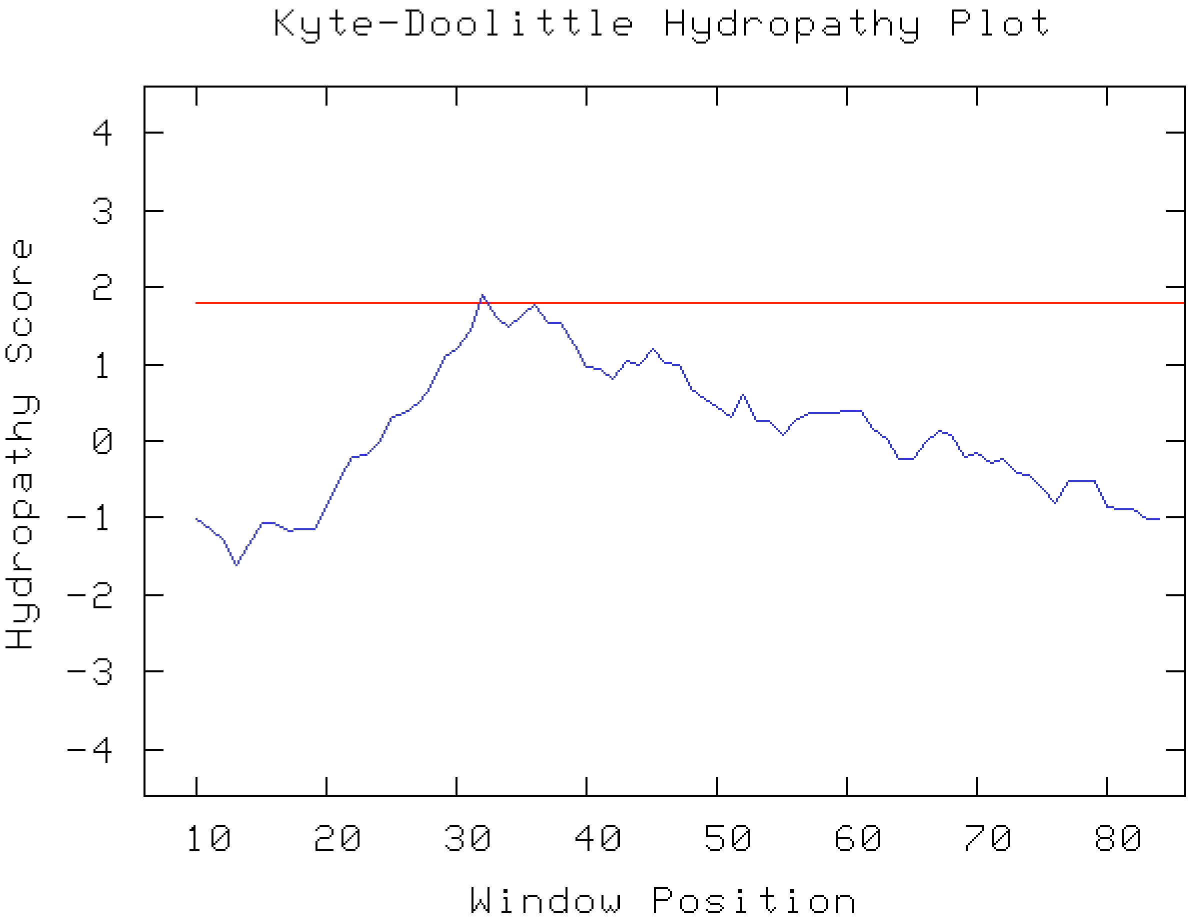

Kyte-Doolittle Hydropathy Plot
Hydropathy Score
4
3
2
1
0
-1
-2
-3
-4
10
20
30
40
50
60
70
80
Window Position

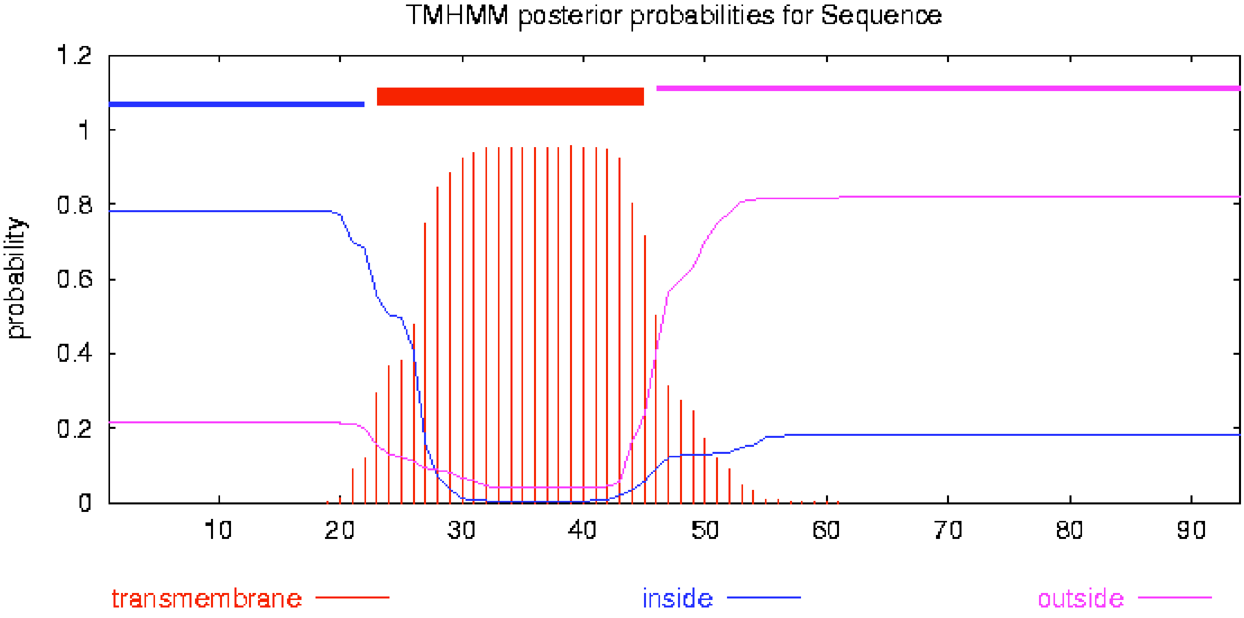

TMHMM posterior probabilities for Sequence
probability
1.2
1
0.8
0.6
0.4
0.2
0
10
20
30
40
50
60
70
80
90
transmembrane
inside
outside

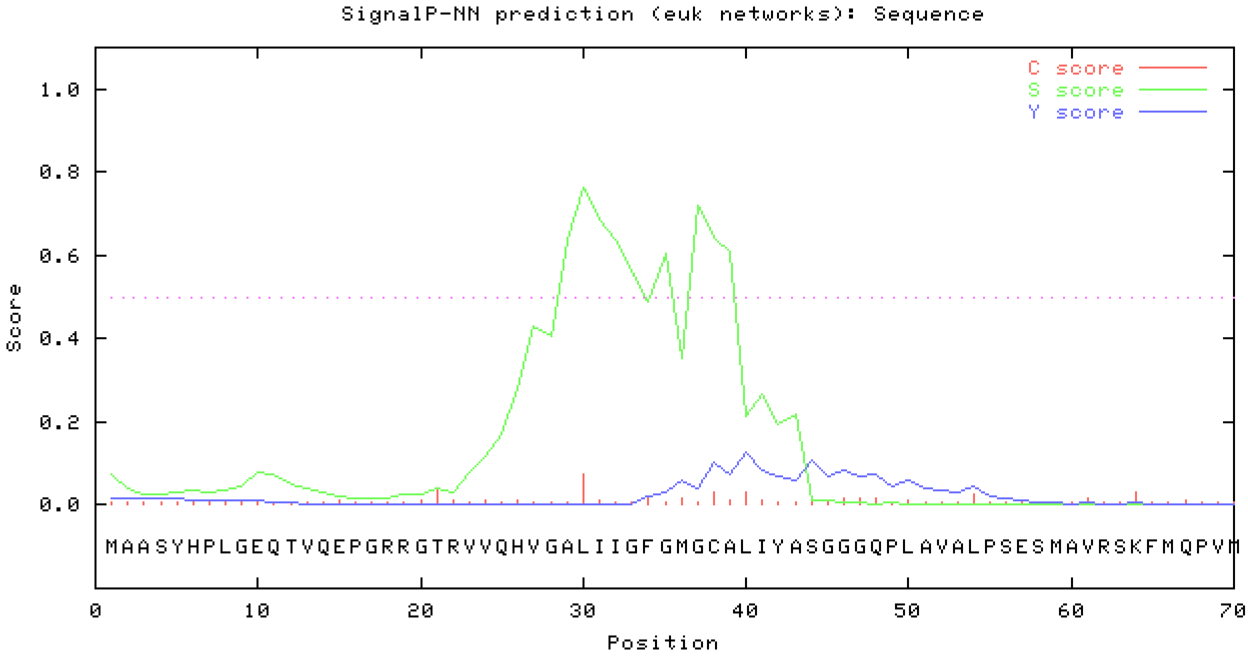

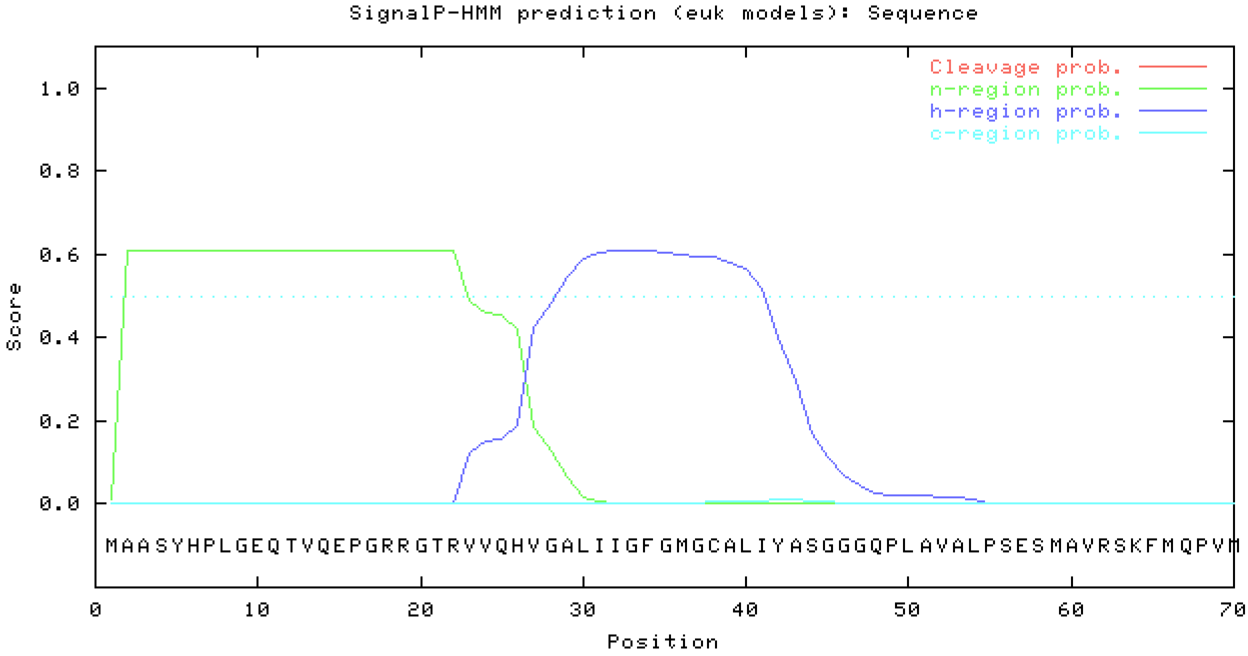

After N-24aa cut

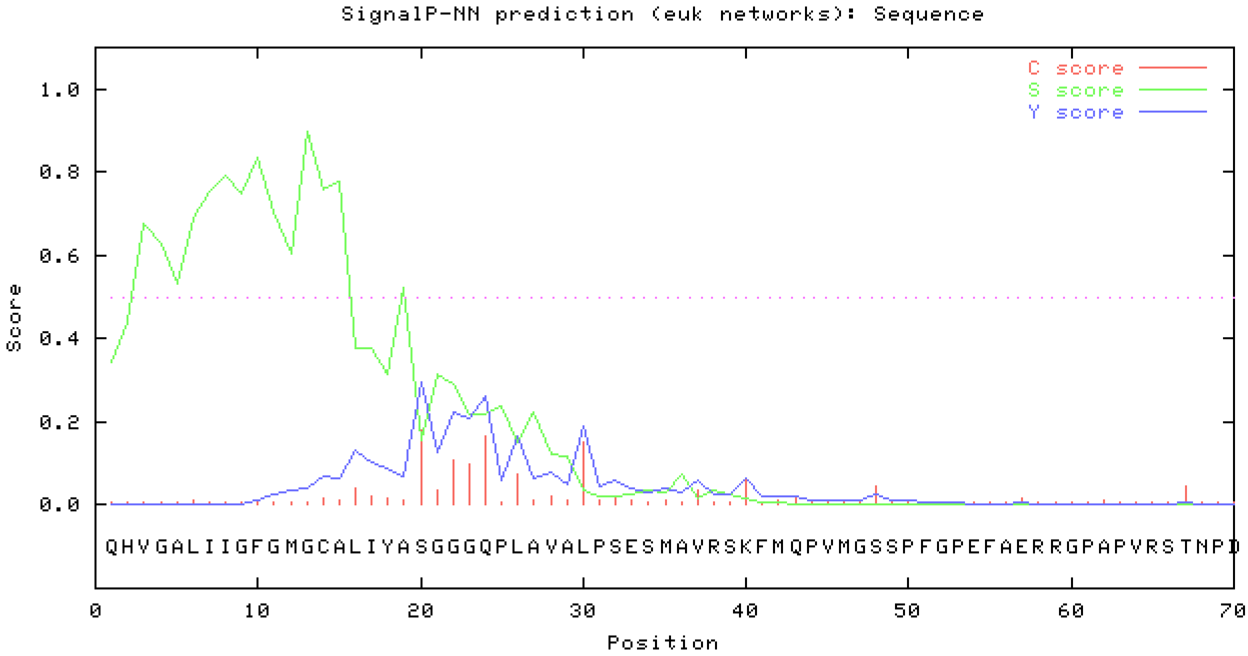

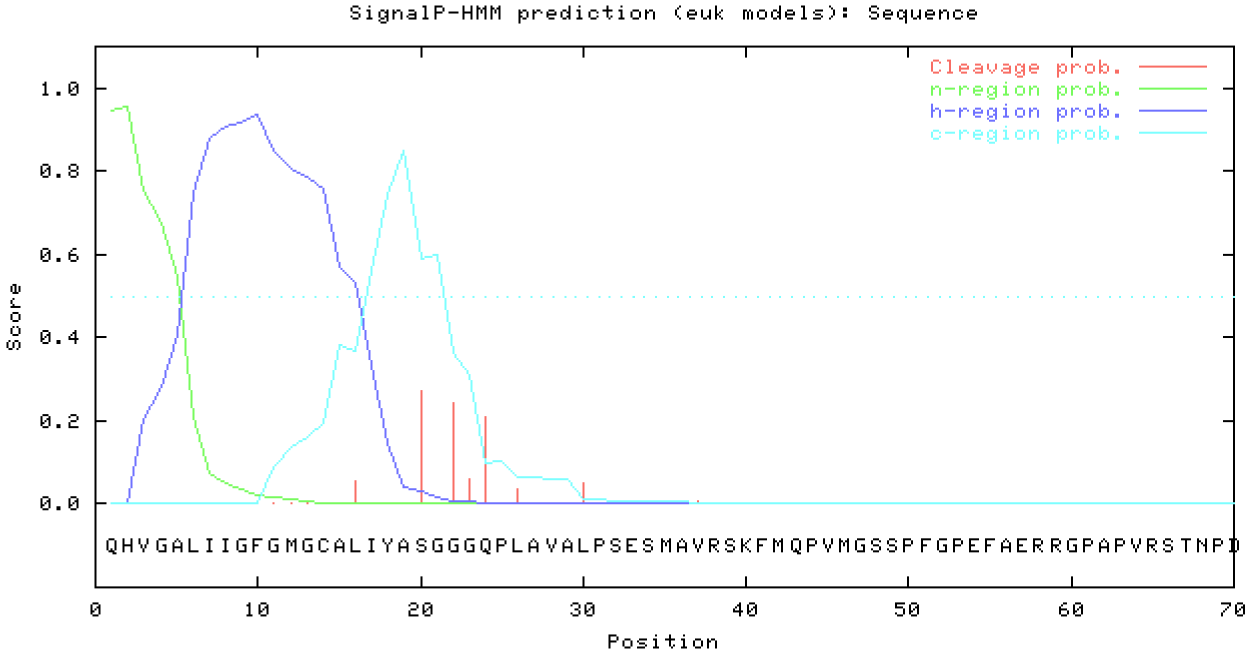

>hcf136_signal_transit_sequence

MQHPFFAEAGPESASSSGRYNSKSLVLISLLIGLGCVAFWSQSGLVKQPLPSISMSSIYSQPALSPTHGRLAKAPQPTQA

WTRQLGPSFWQLTKSASMSLPFLQPVRAEGEAAEDSVPLSASSEFTHGRRDVLRTAGLAMAIEAGSLGRSAKAERIES

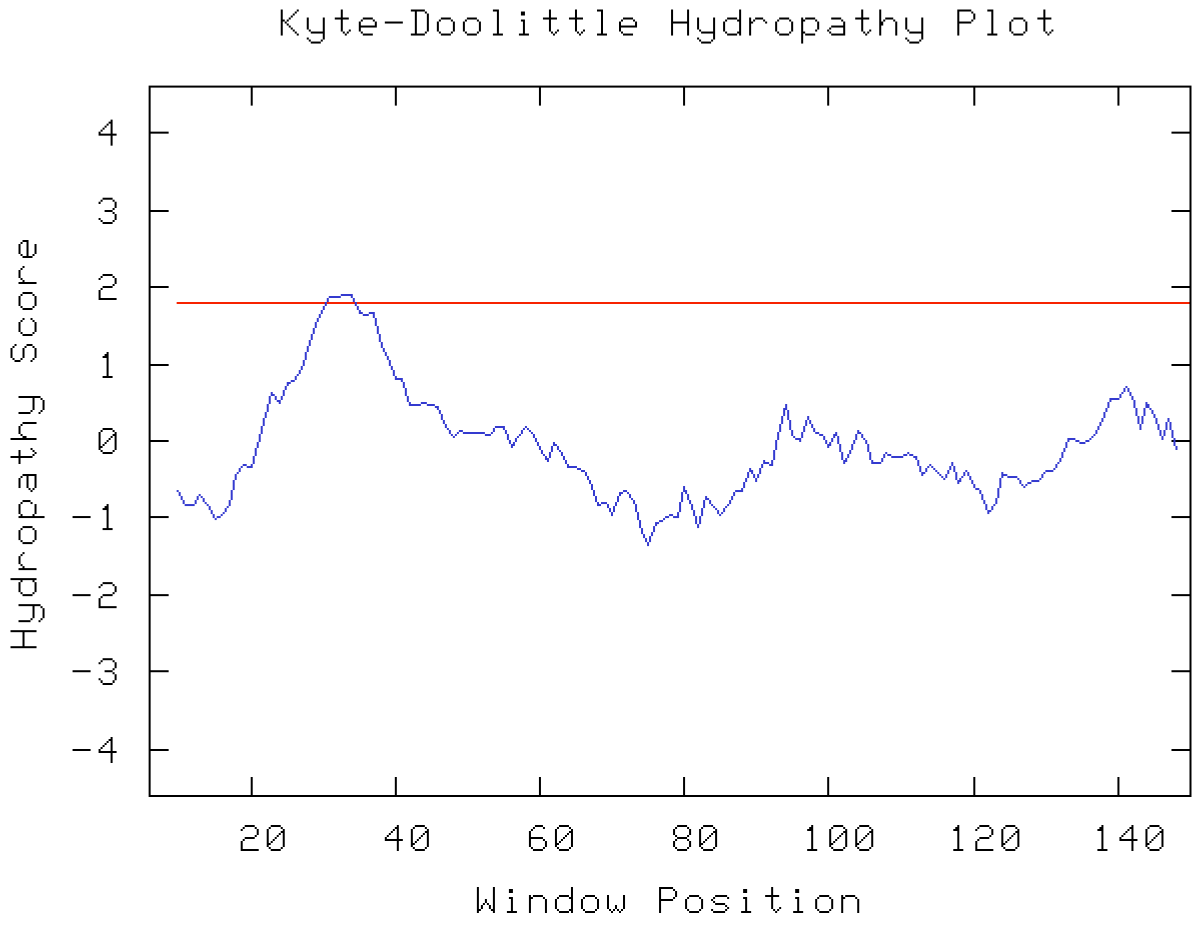

Kyte-Doolittle Hydropathy Plot
Hydropathy Score
4
3
2
1
0
-1
-2
-3
-4
20
40
60
80
100
120
140
Window Position

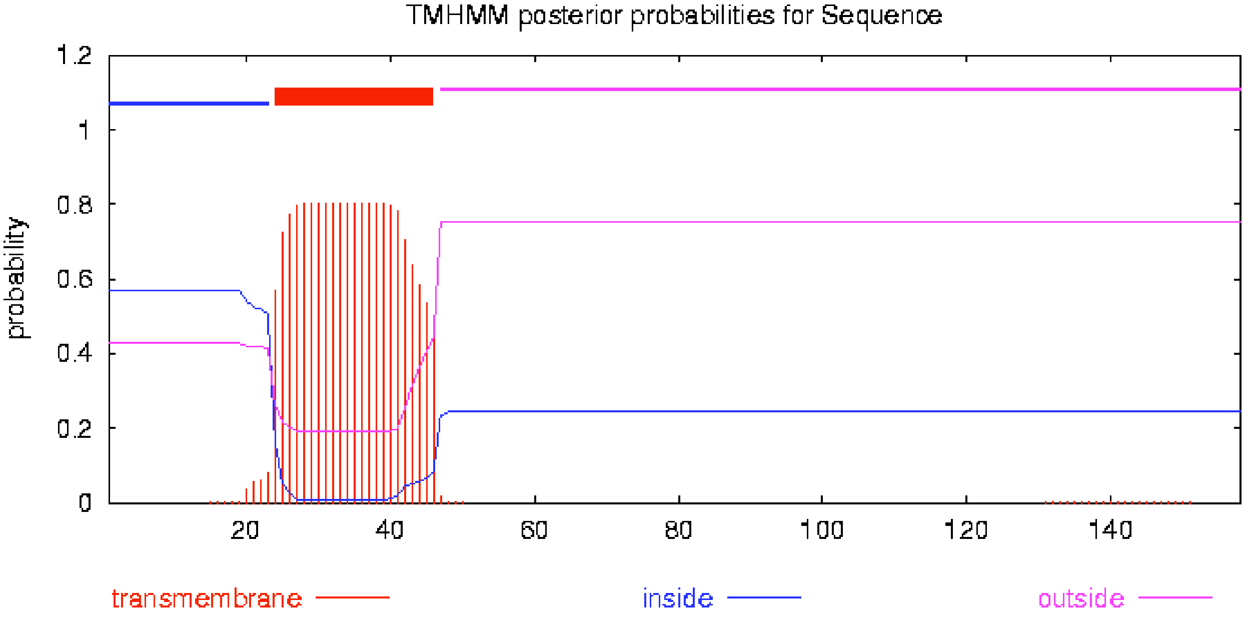

TMHMM posterior probabilities for Sequence
probability
1.2
1
0.8
0.6
0.4
0.2
0
20
40
60
80
100
120
140
transmembrane
inside
outside

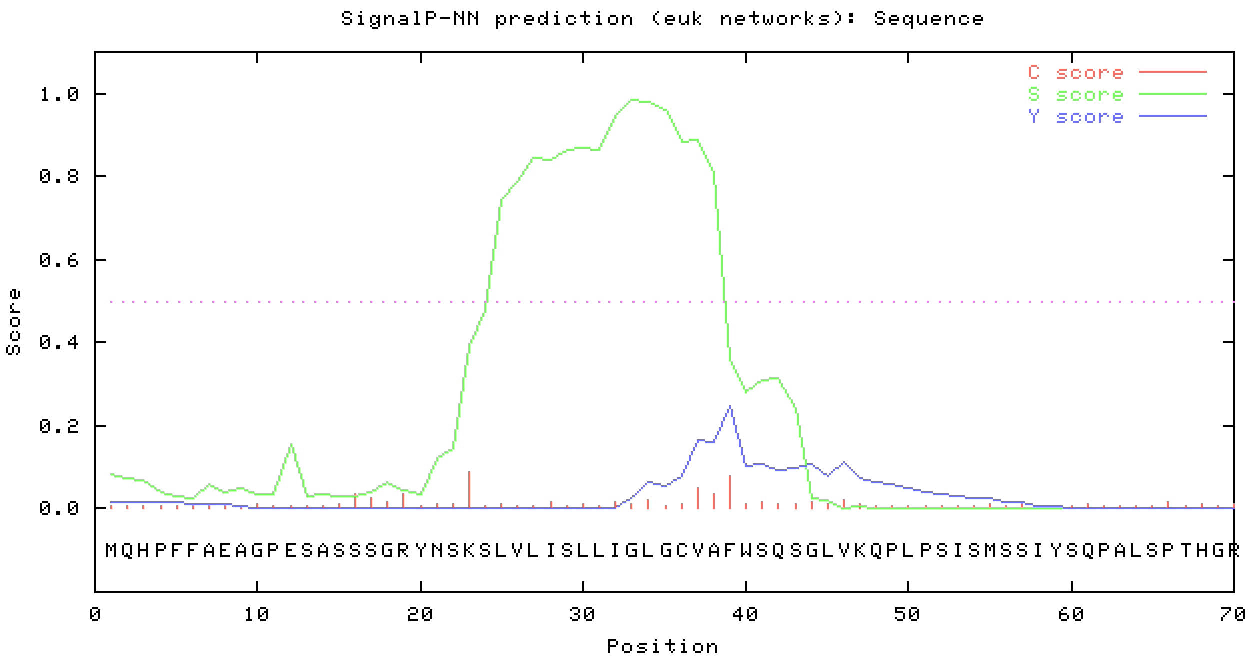

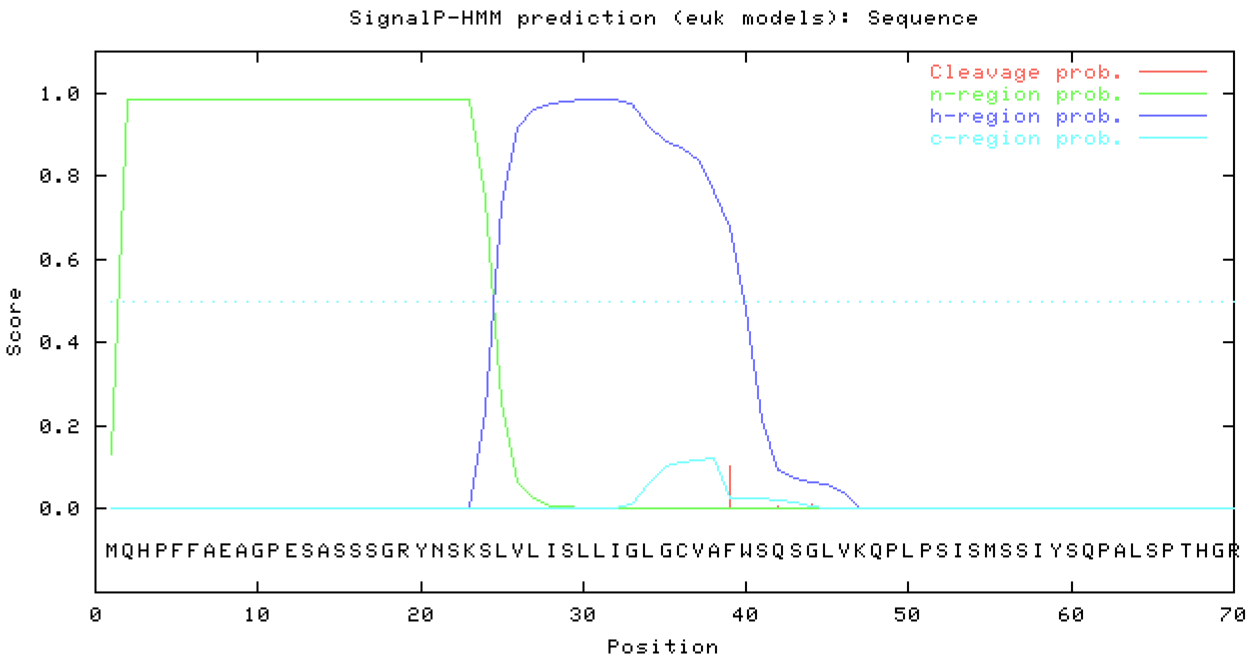

After N-24aa cut

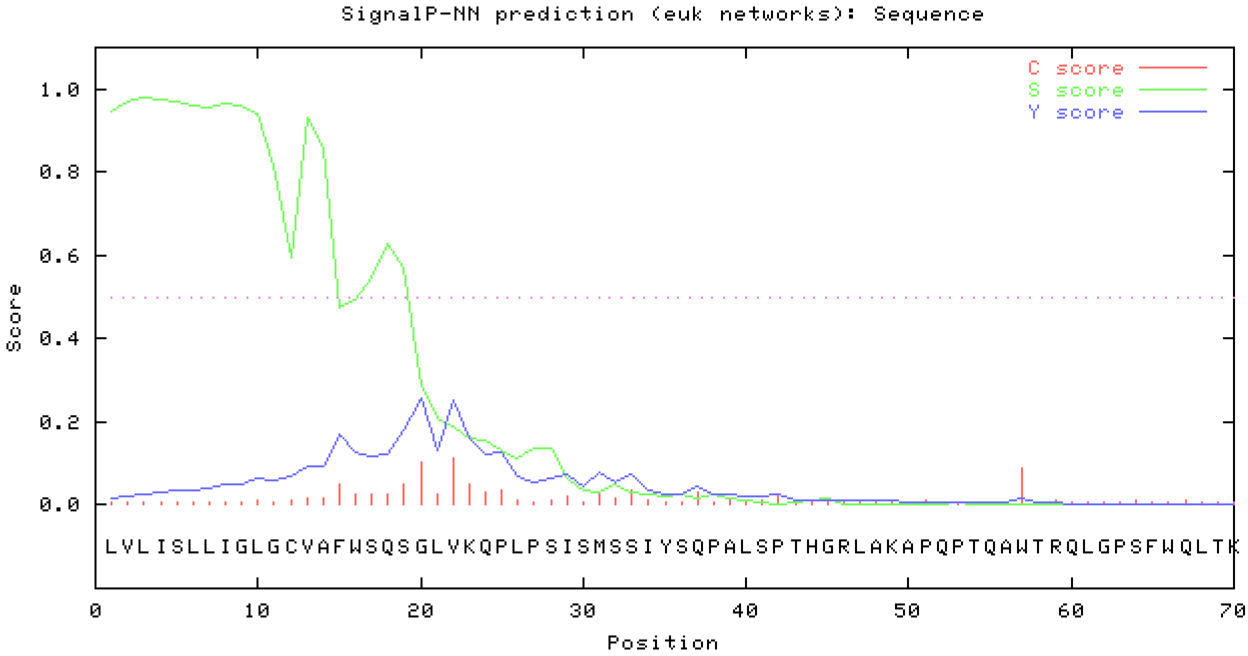

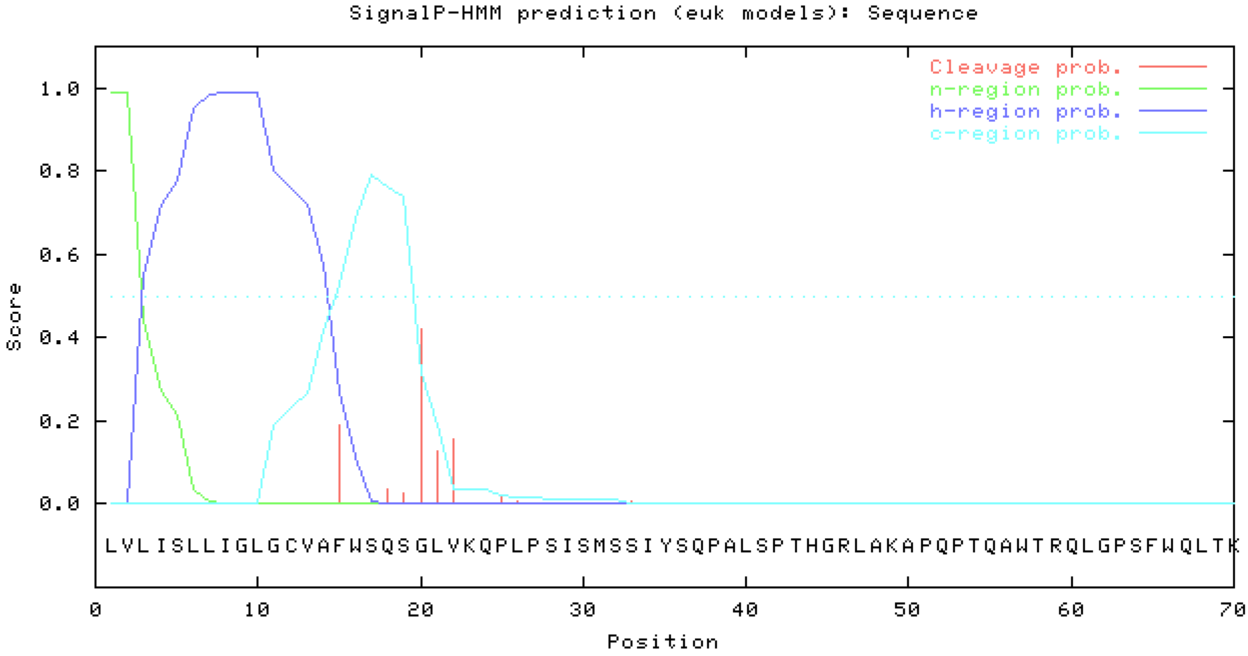

>prk_signal_transit_sequence

MSVAIHAVAEPLLQETSLAAHSGRKYLPLFALLLGIFASVAVFWSPVPSAGNVFEHQPVVEKVAIDMAASLSRGLVQSPQ

MRQPLDQCMYRAQSNLKATPNRHFGSVRTAAL

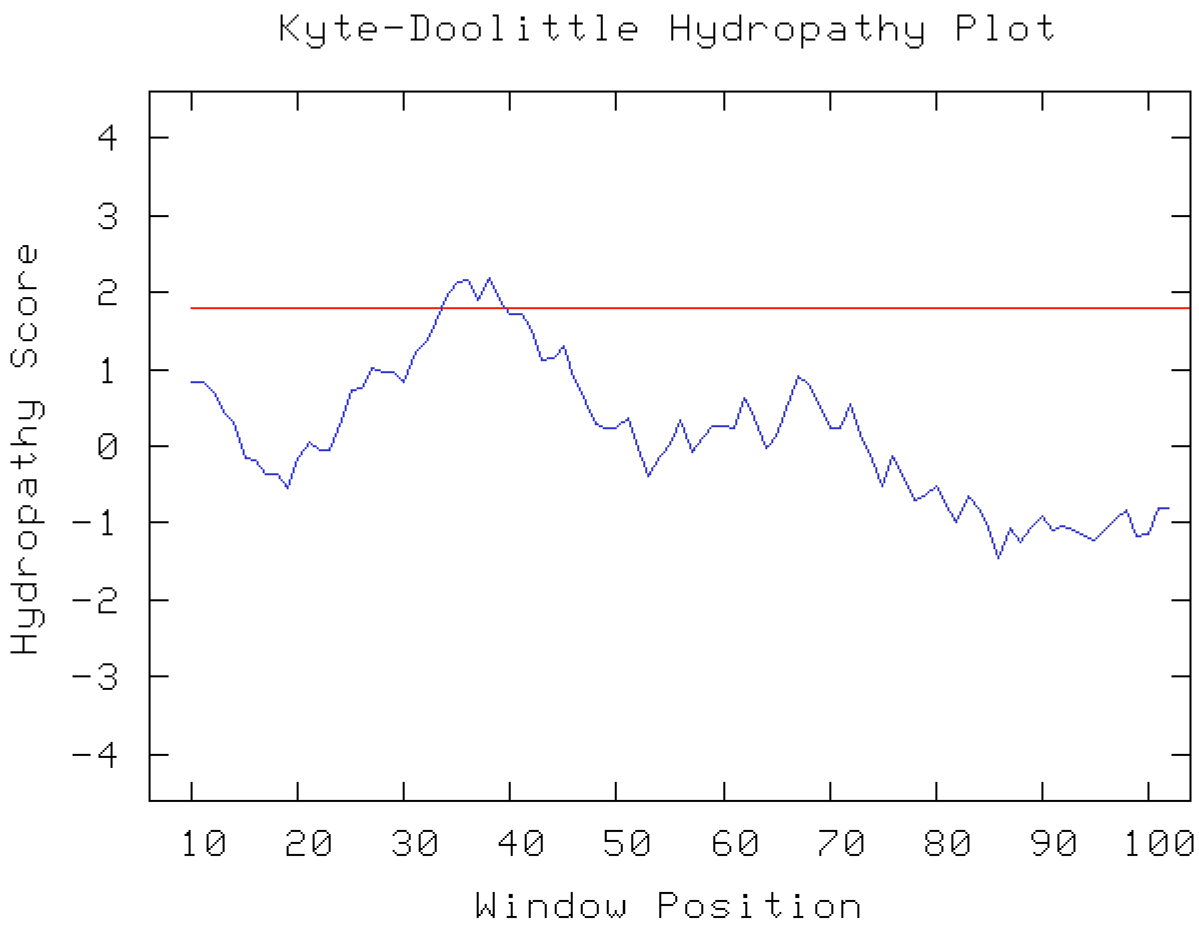

Kyte-Doolittle Hydropathy Plot
Hydropathy Score
4
3
2
1
0
-1
-2
-3
-4
10
20
30
40
50
60
70
80
90
100
Window Position

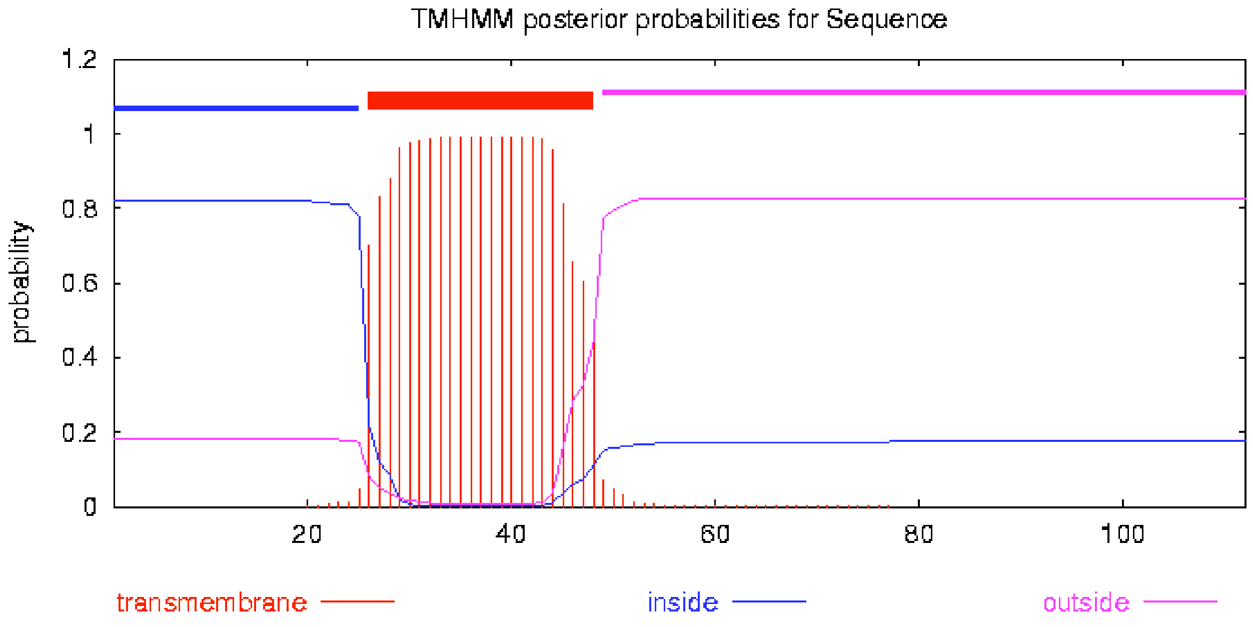

TMHMM posterior probabilities for Sequence
probability
1.2
1
0.8
0.6
0.4
0.2
0
20
40
60
80
100
transmembrane
inside
outside

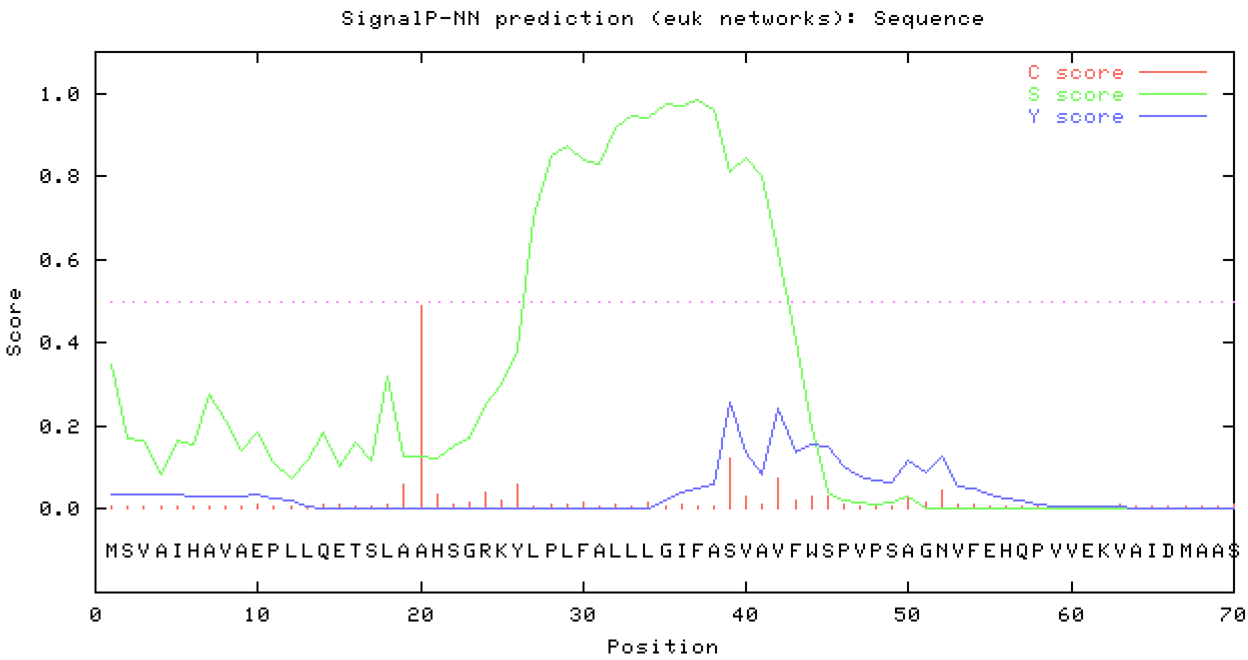

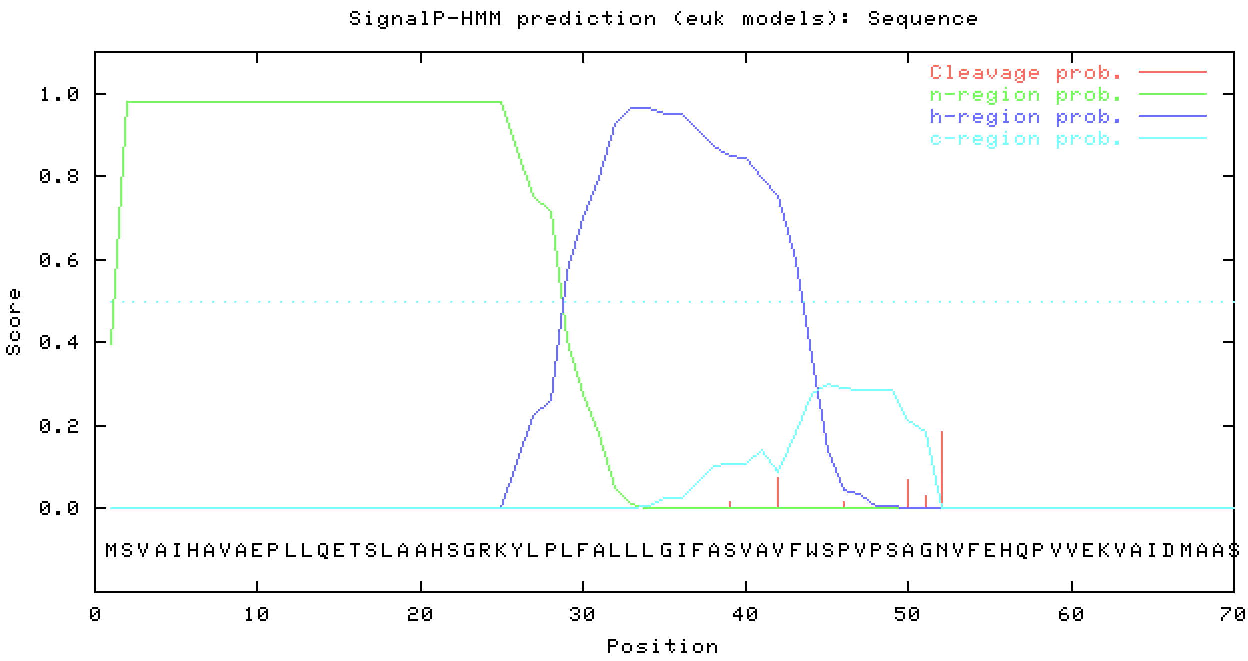

After N-26aa cut

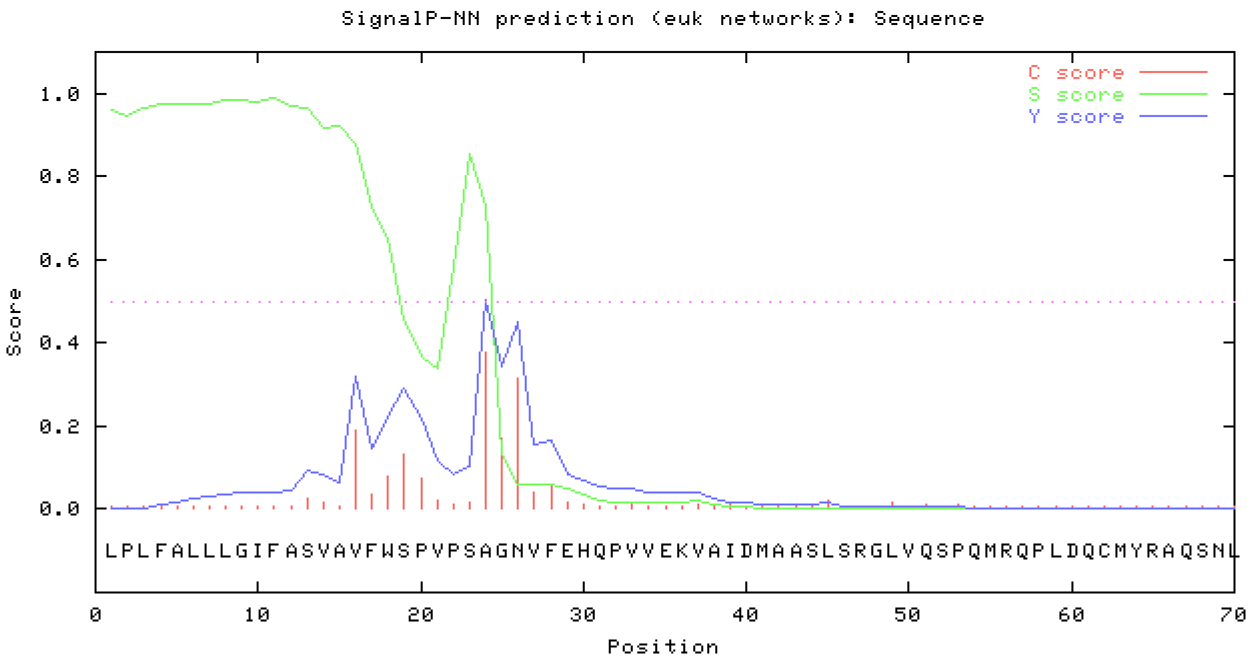

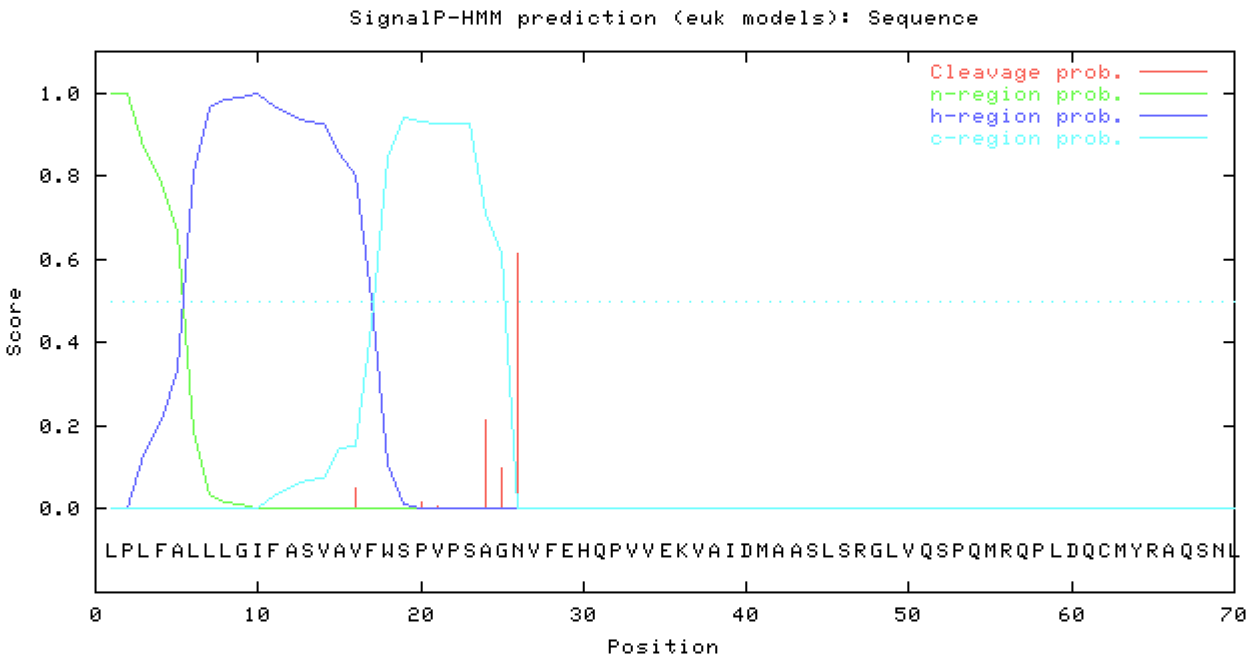

>psbO_signal_transit_sequence

MTLQEFSGKAKVLALISLLLLAAGLLS*LPFTNVDEGAIIGMAAAFSKATKVSQLTQPARIRLPYAGQLMQPTGWQYPV

TEEERSAVSVNSGRRELMNAATFASAATSVAKKAHA

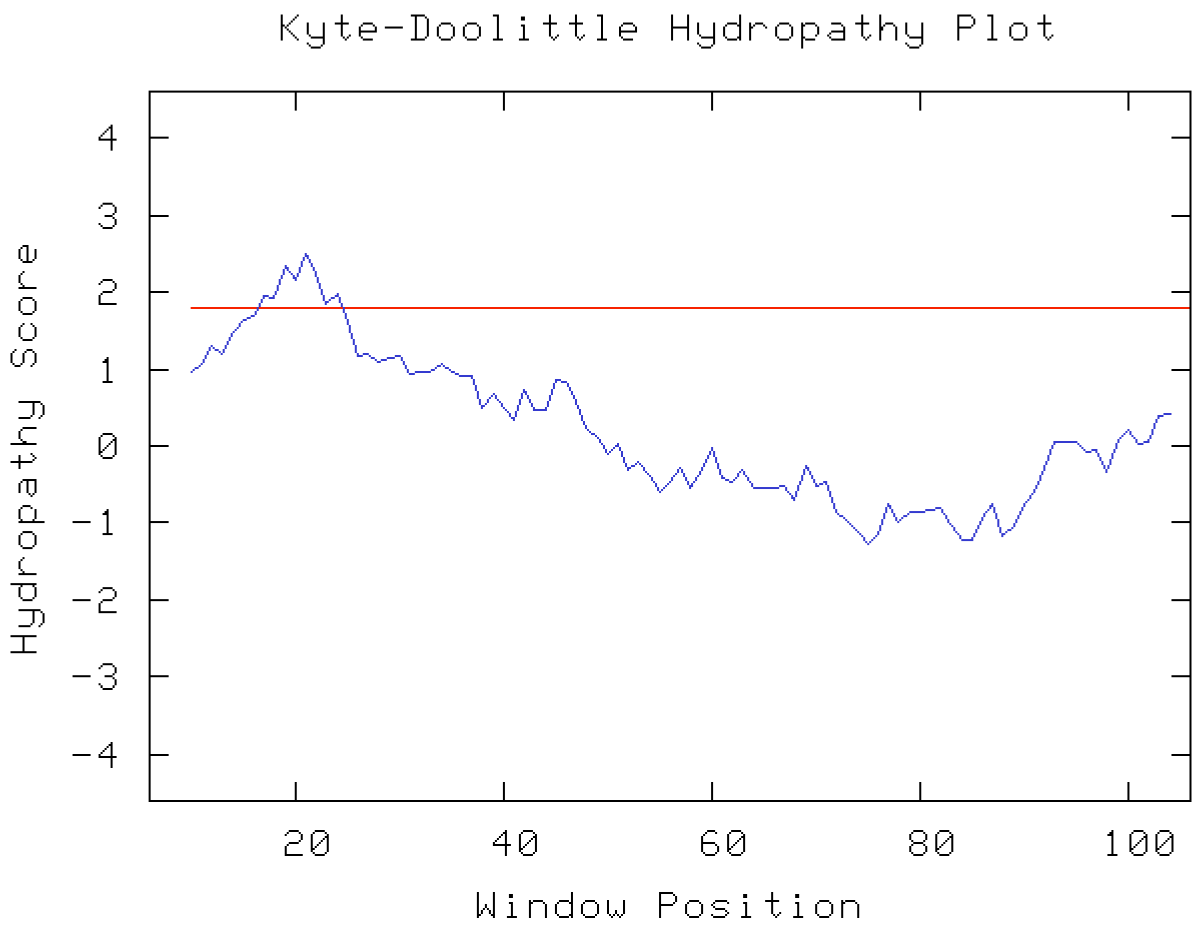
Kyte-Doolittle Hydropathy Plot
Hydropathy Score
4
3
2
1
0
-1
-2
-3
-4
20
40
60
80
100
Window Position

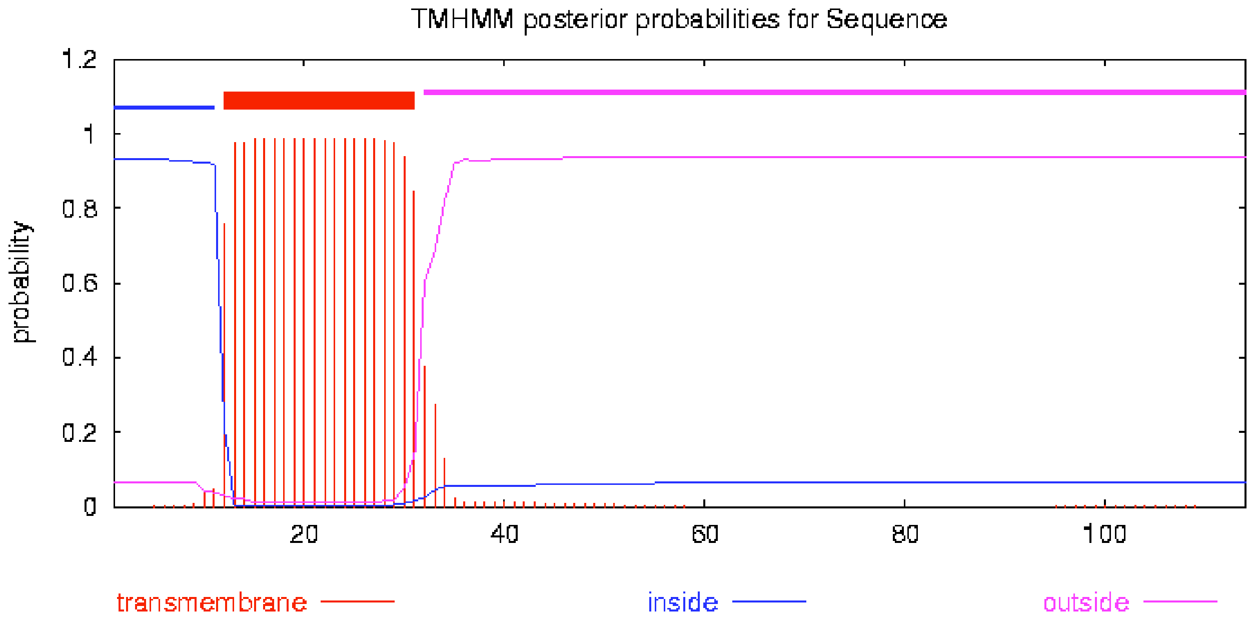
TMHMM posterior probabilities for Sequence
probability
1.2
1
0.8
0.6
0.4
0.2
0
20
40
60
80
100
transmembrane
inside
outside

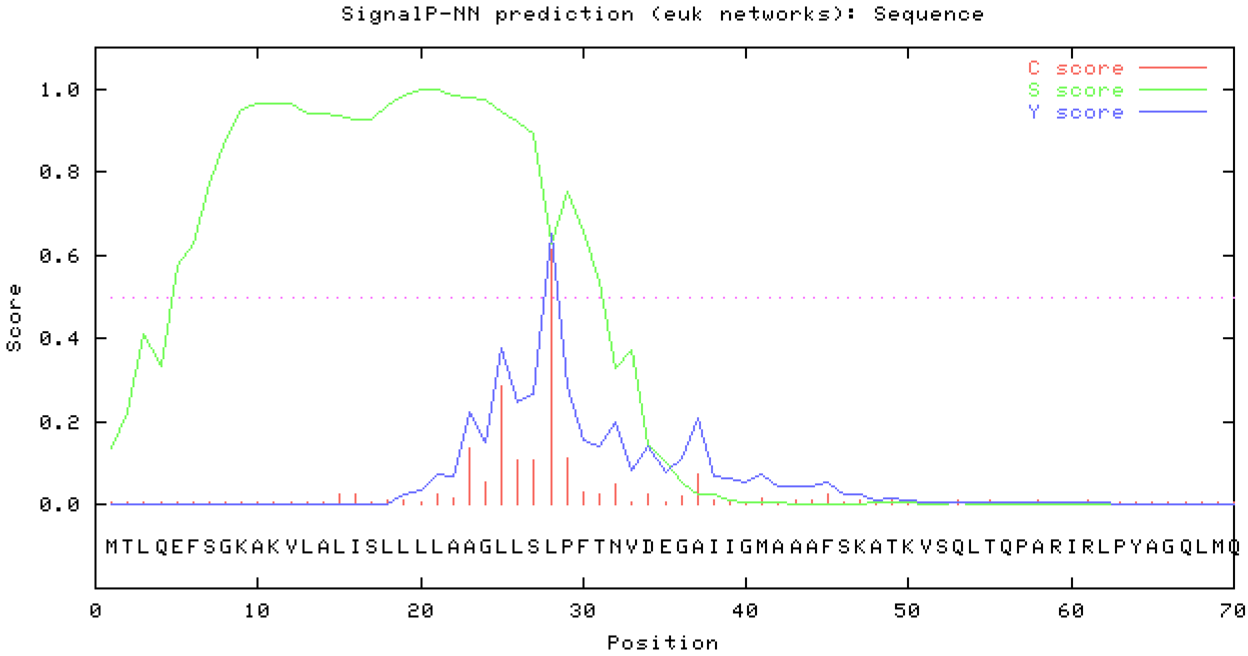

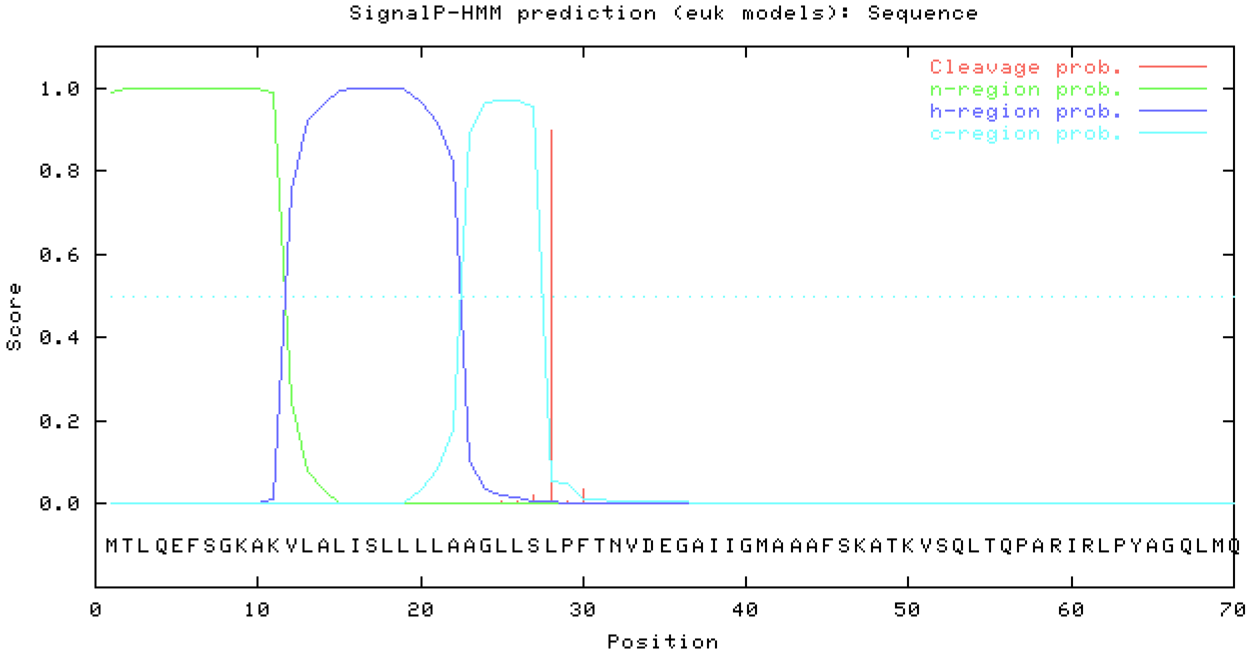

>psbP_signal_transit_sequence

MILRGSSLRSAHSERKTVLVSLLLGCAFAALLSLPQTRHEEDLATTLALASSAMTQPRFARQQMQRASAFTSEHEHDHSA
AHVEPGMVKSS

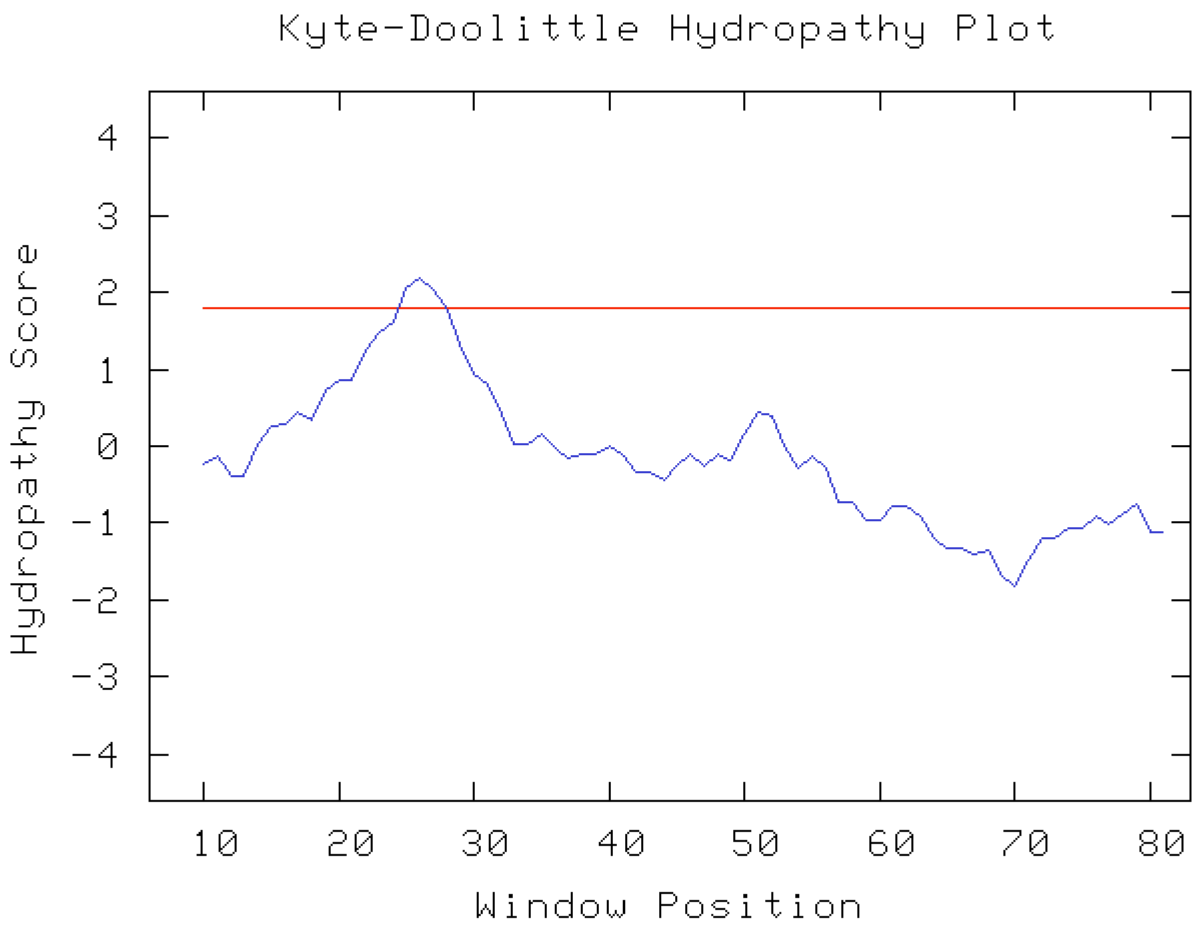

Kyte-Doolittle Hydropathy Plot
Hydropathy Score
4
3
2
1
0
-1
-2
-3
-4
10
20
30
40
50
60
70
80
Window Position

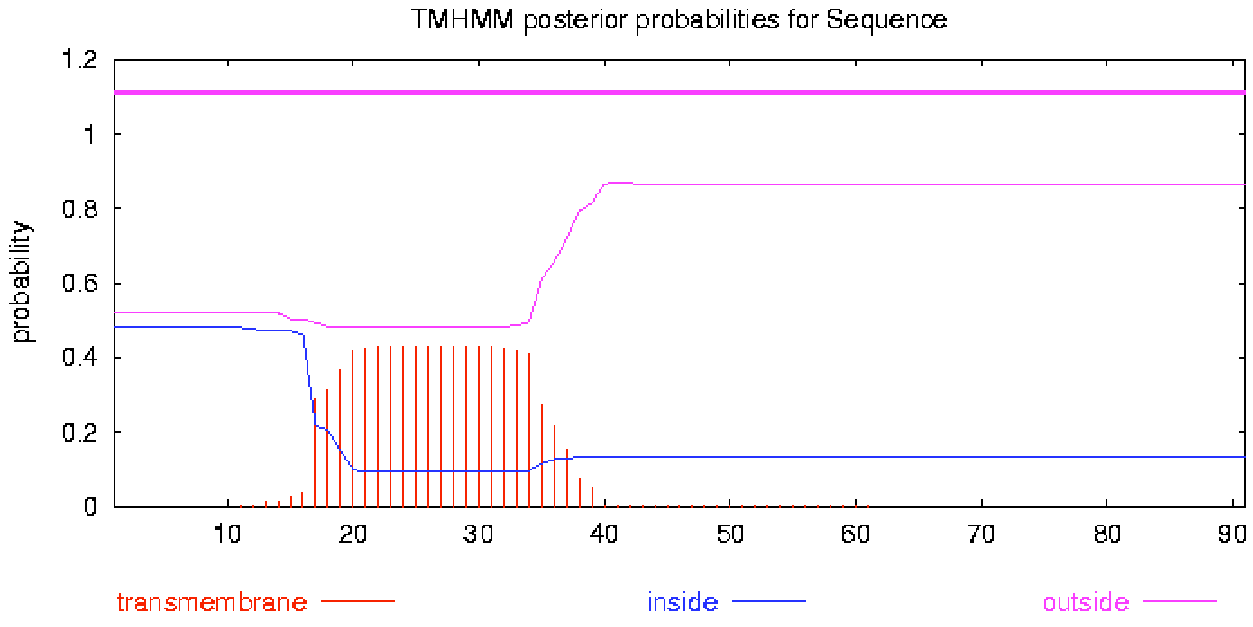

TMHMM posterior probabilities for Sequence
probability
1.2
1
0.8
0.6
0.4
0.2
0
10
20
30
40
50
60
70
80
90
transmembrane
inside
outside

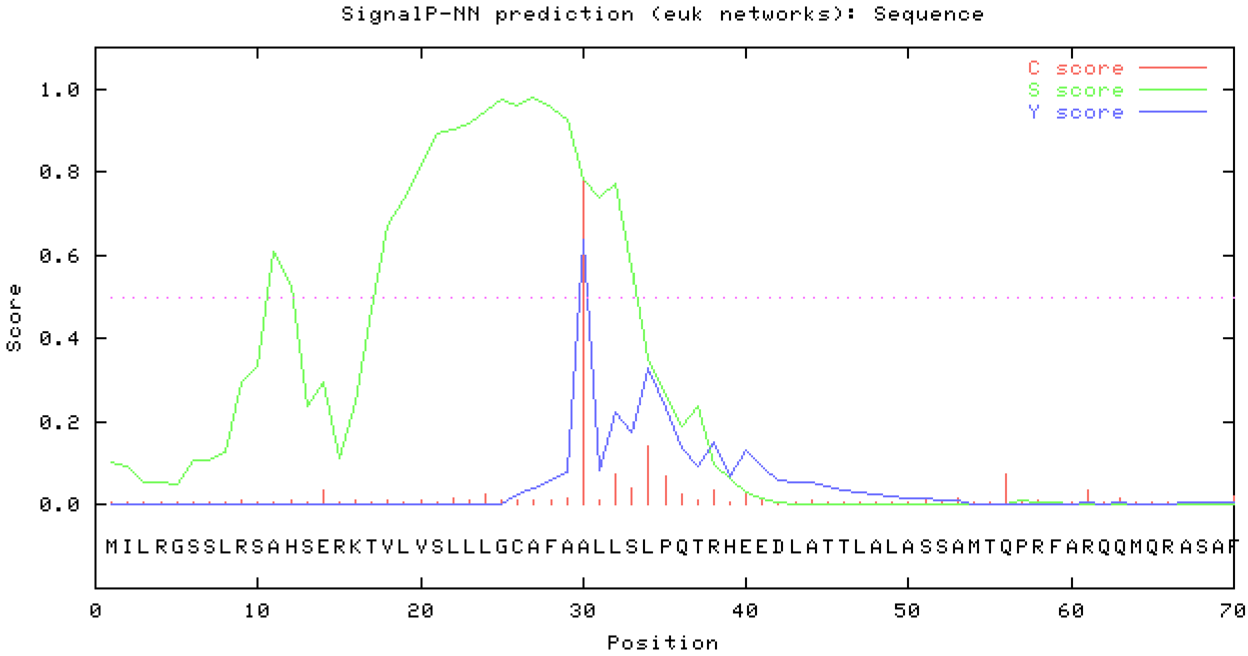

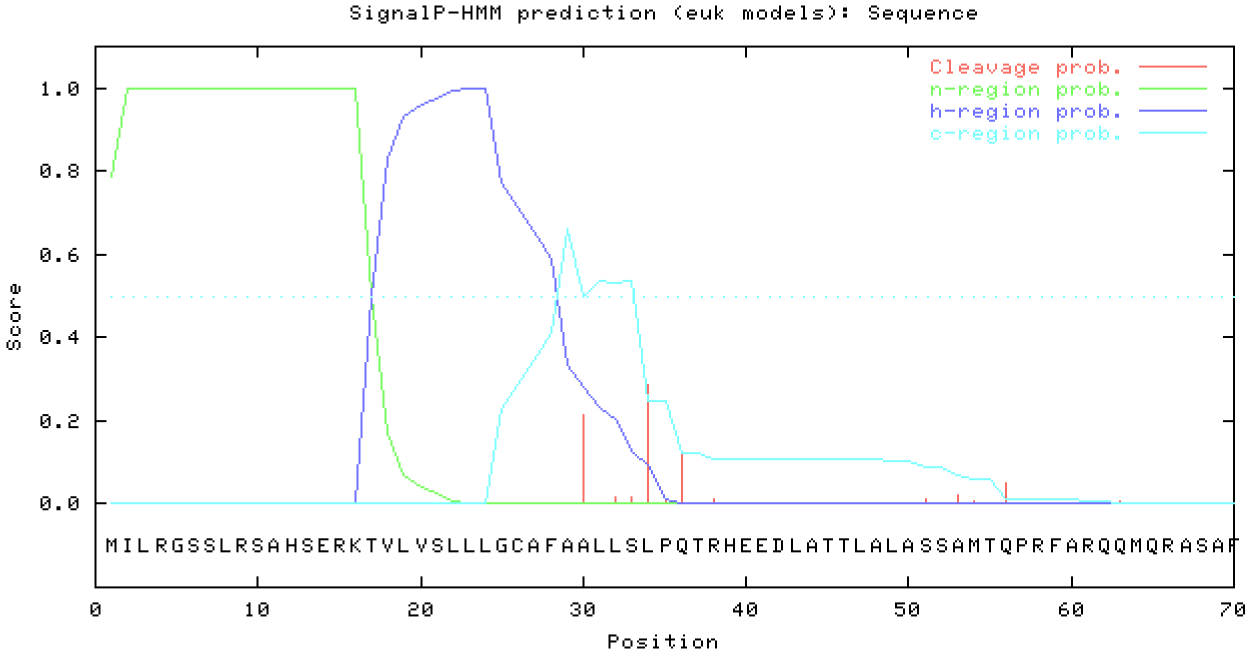

After N-9aa cut

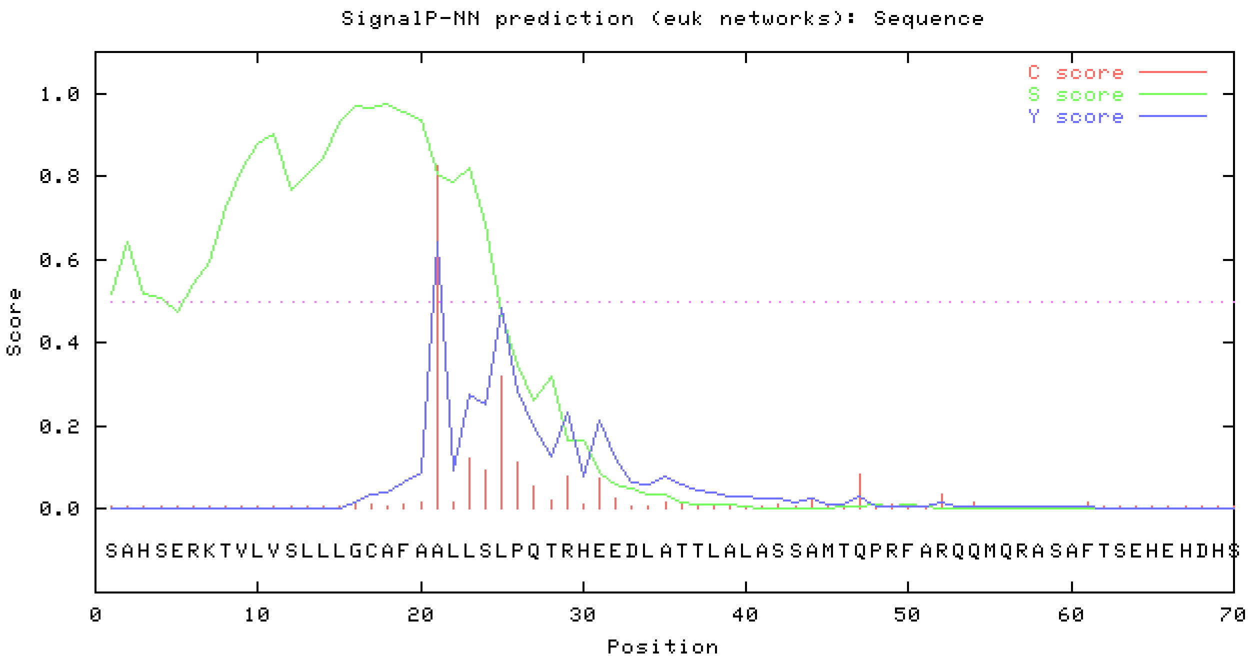

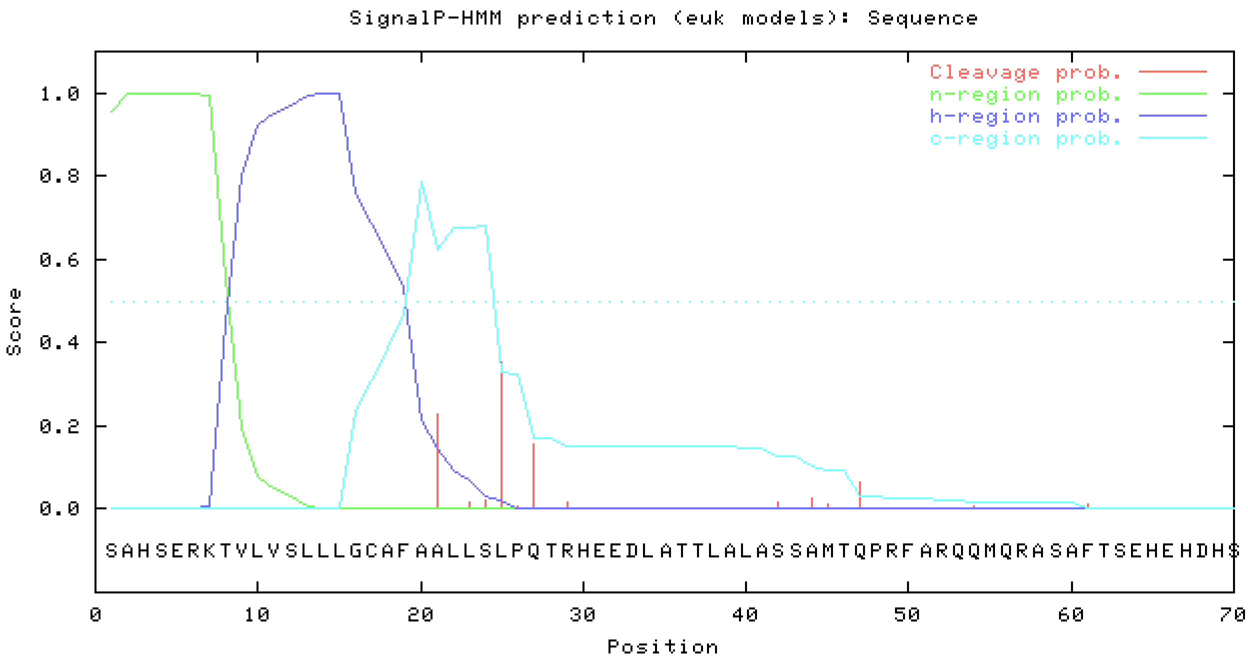

>psbR_signal_transit_sequence

MAVPYLALGEAVVPSRARSTLKMIVFPFLIGFGCALIWMSGRGDQPLEDFDGATSMIAEPMQLARQAMPTRAMPNTRQLV
IVEGGKGGKVNTGLSSVEDVVVK

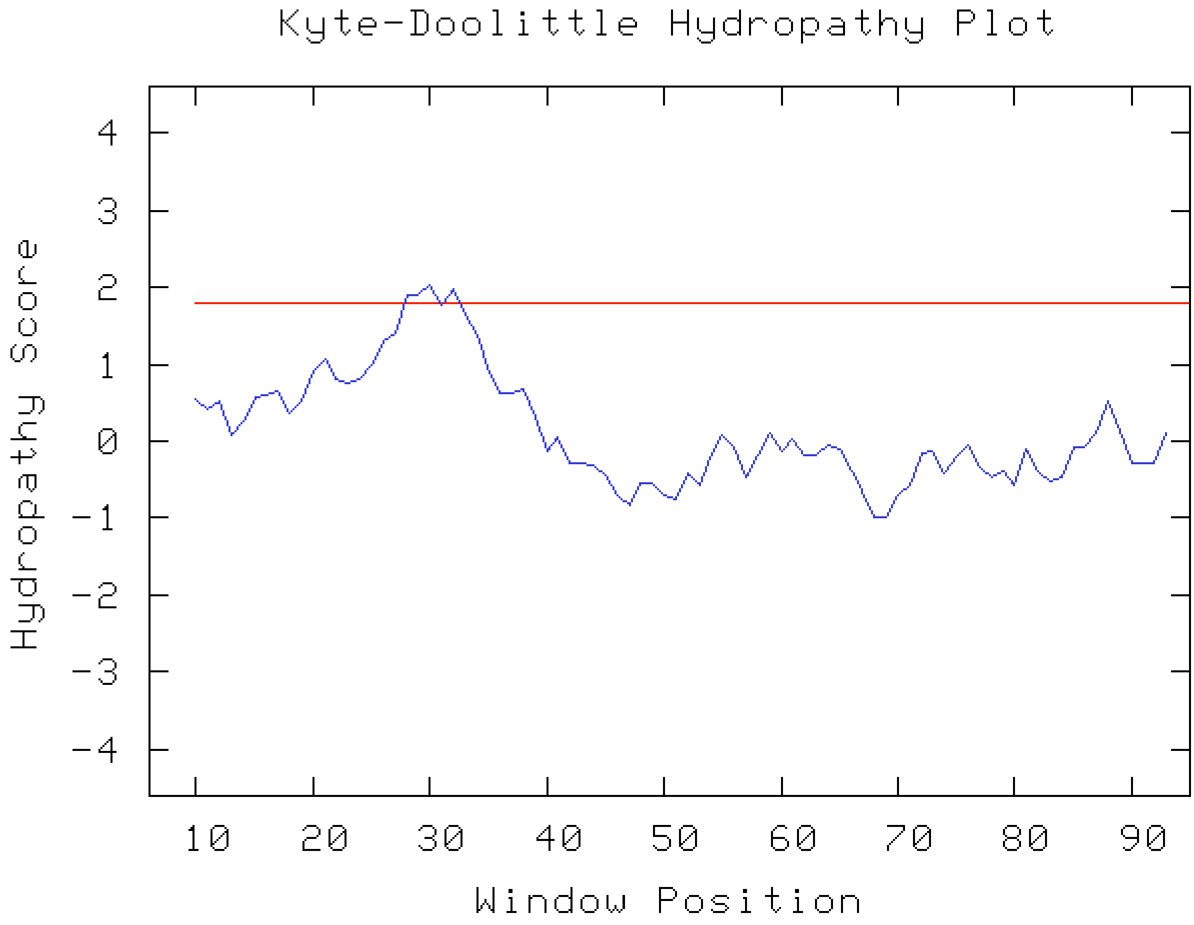

Kyte-Doolittle Hydropathy Plot
Hydropathy Score
Window Position
4
3
2
1
0
-1
-2
-3
-4
10
20
30
40
50
60
70
80
90

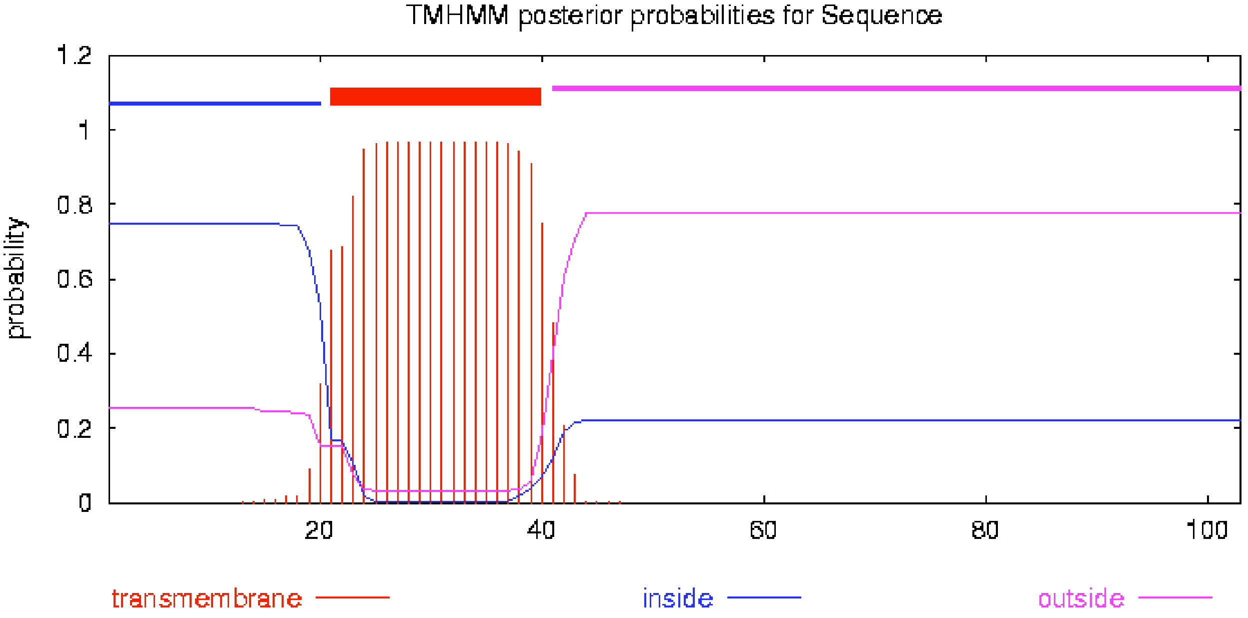

TMHMM posterior probabilities for Sequence
probability
1.2
1
0.8
0.6
0.4
0.2
0
20
40
60
80
100
transmembrane
inside
outside

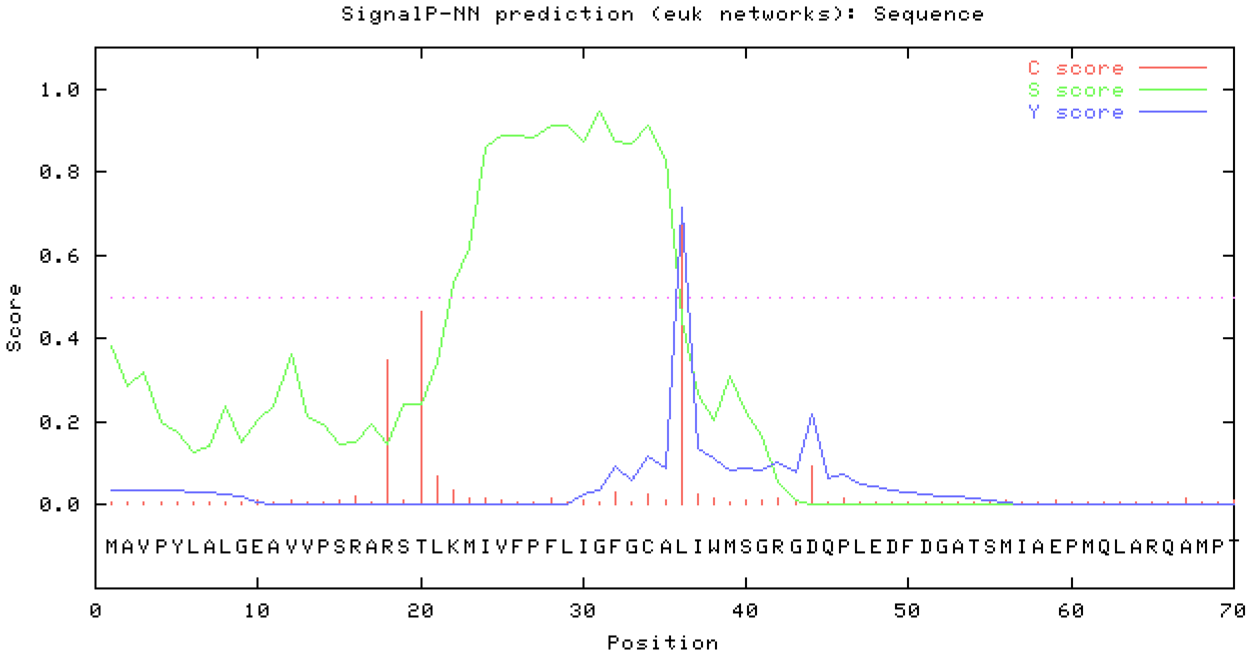

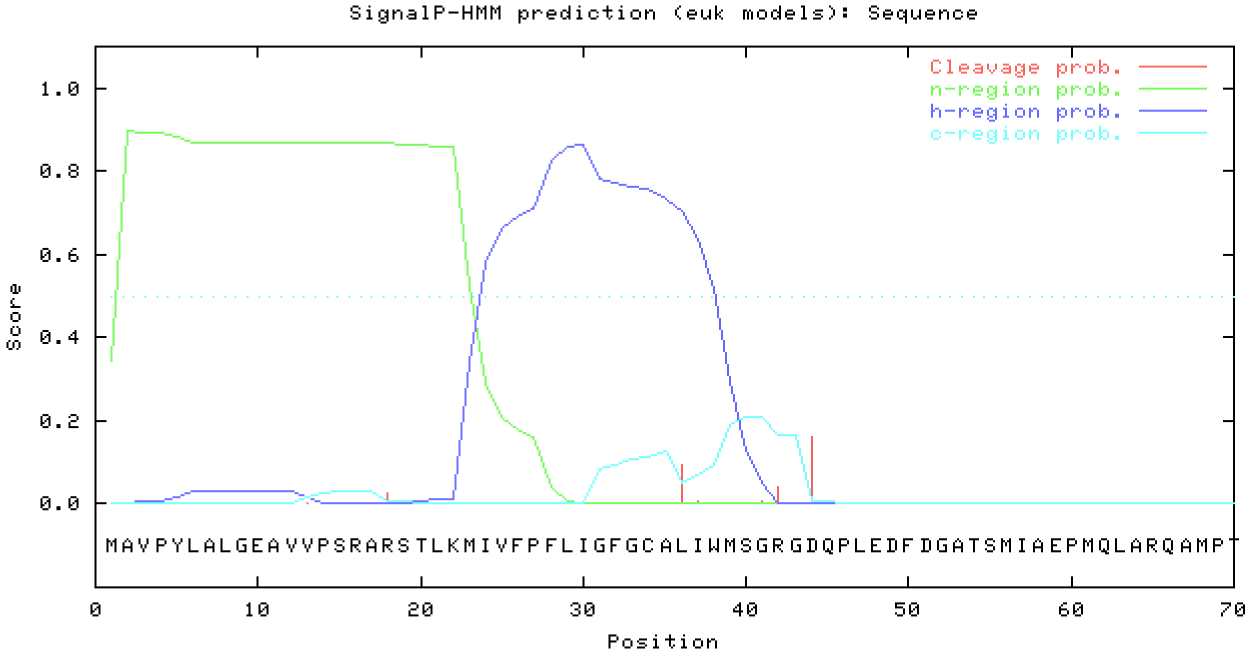

After N-21aa cut

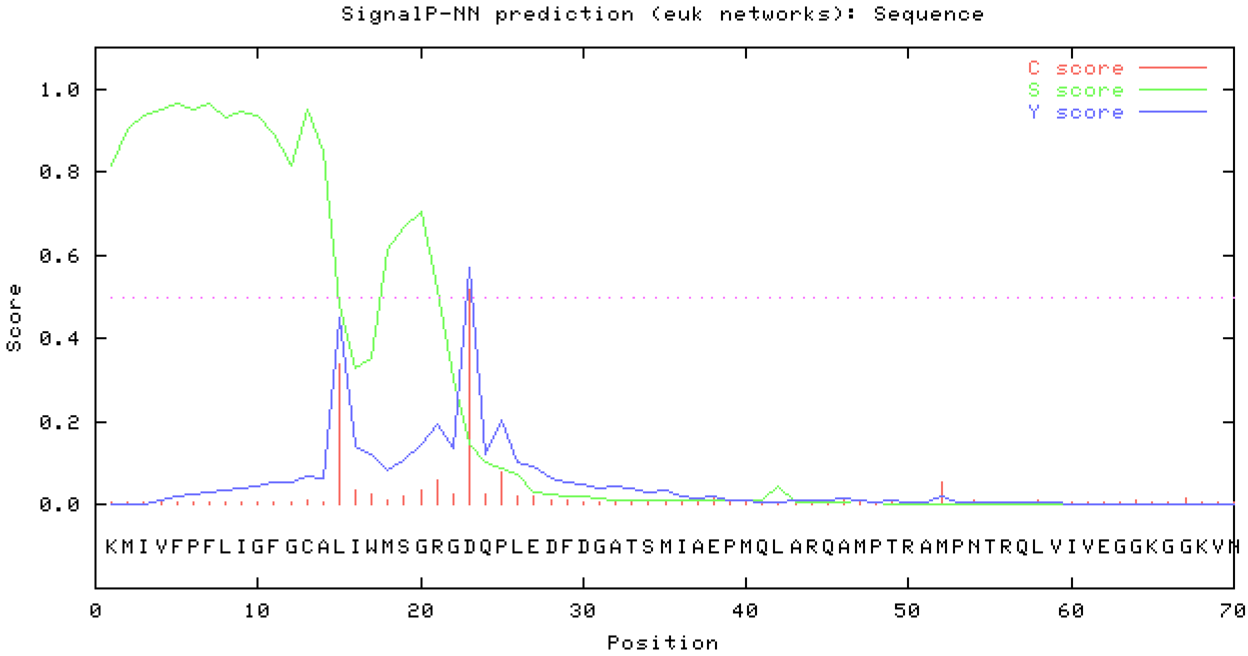

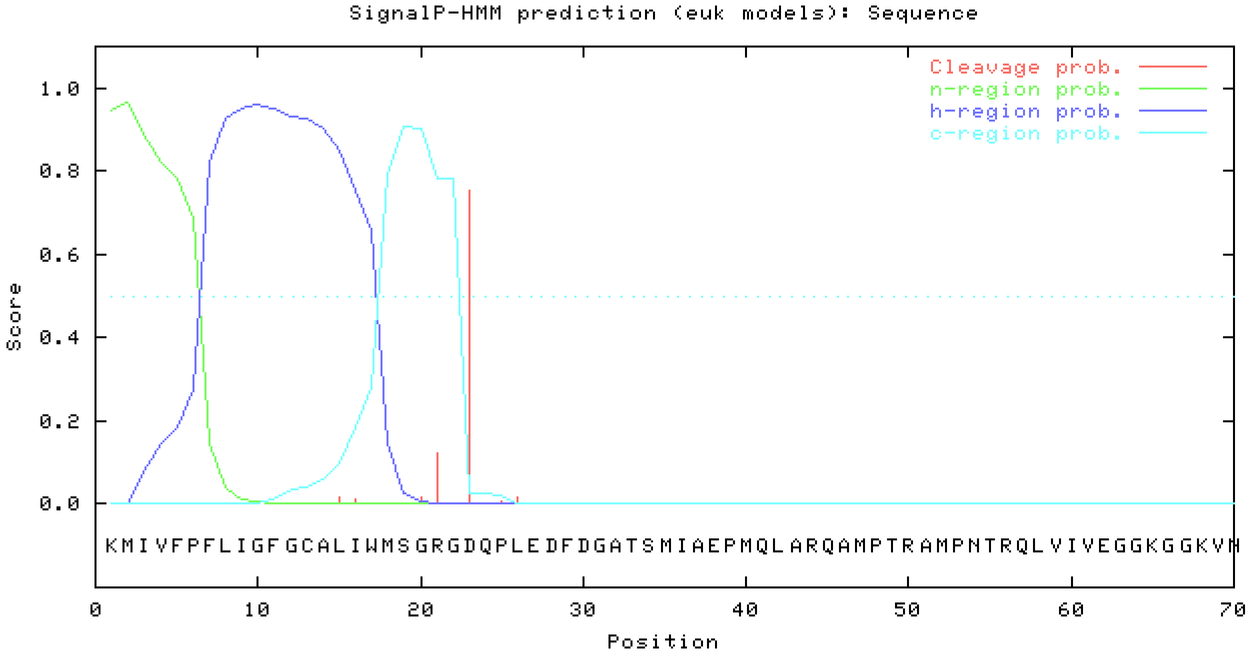

>rbcS_signal_transit_sequence

MAGSYLPFAEESGRRSAGLVHHVATMLVGFGLGCAMIYAAGGQPLAVIMPTNNMAAQVIQPAQGQFLEPLAGRPSPVRDQ
PLAAAHGAMQTAQIQ

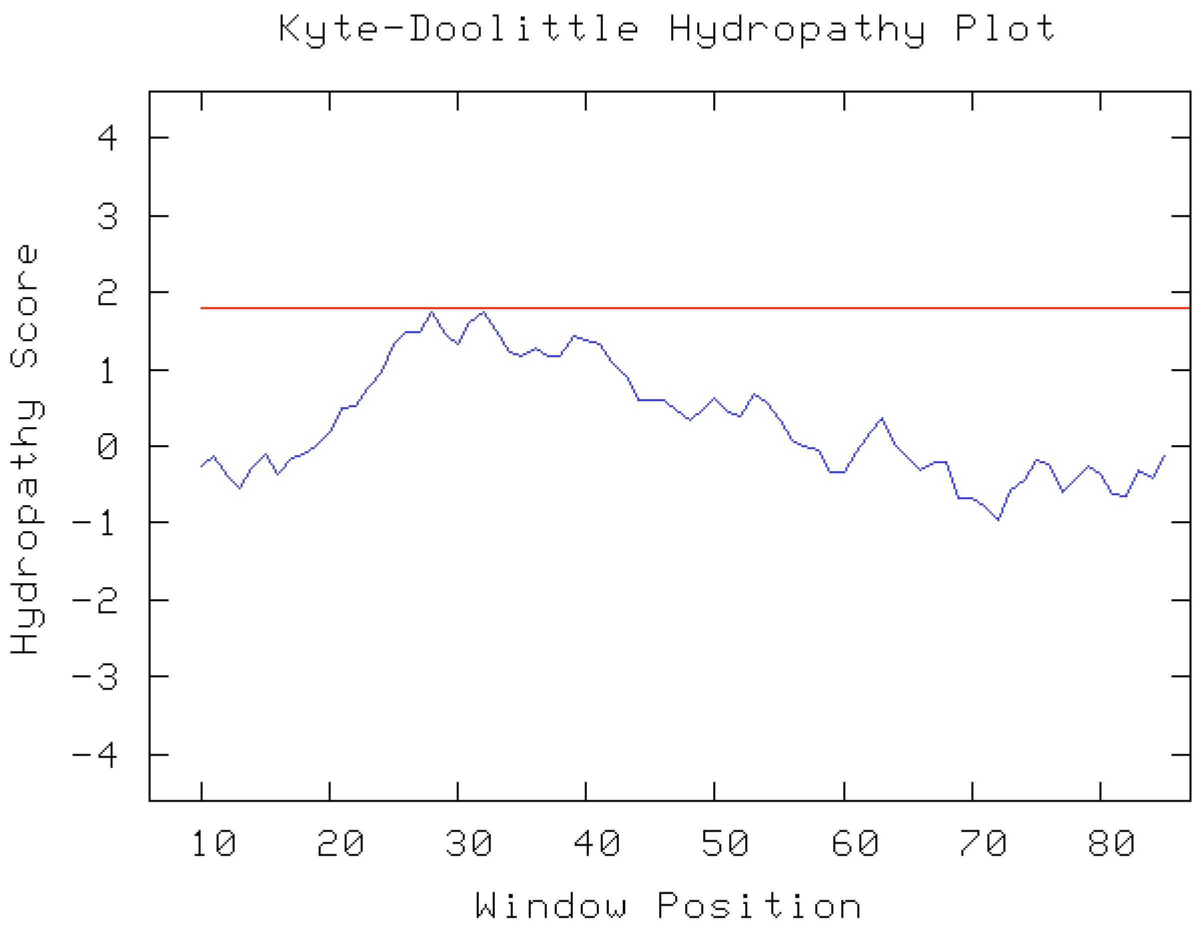
Kyte-Doolittle Hydropathy Plot
Hydropathy Score
4
3
2
1
0
-1
-2
-3
-4
10
20
30
40
50
60
70
80
Window Position

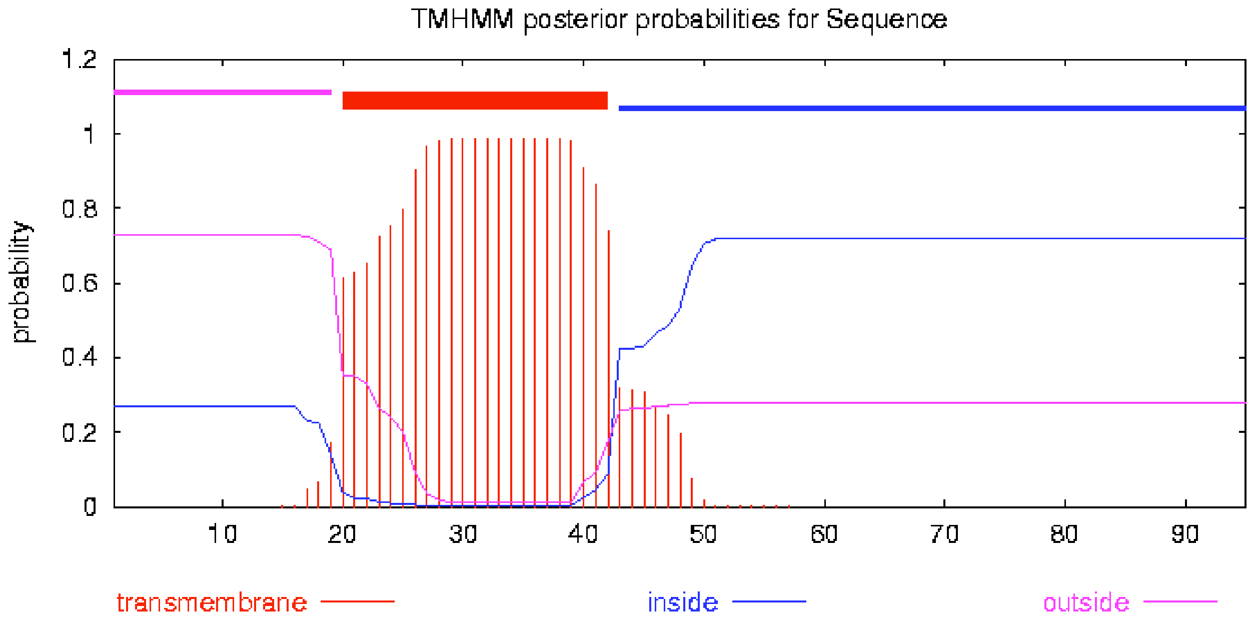
TMHMM posterior probabilities for Sequence
probability
1.2
1
0.8
0.6
0.4
0.2
0
10
20
30
40
50
60
70
80
90
transmembrane
inside
outside

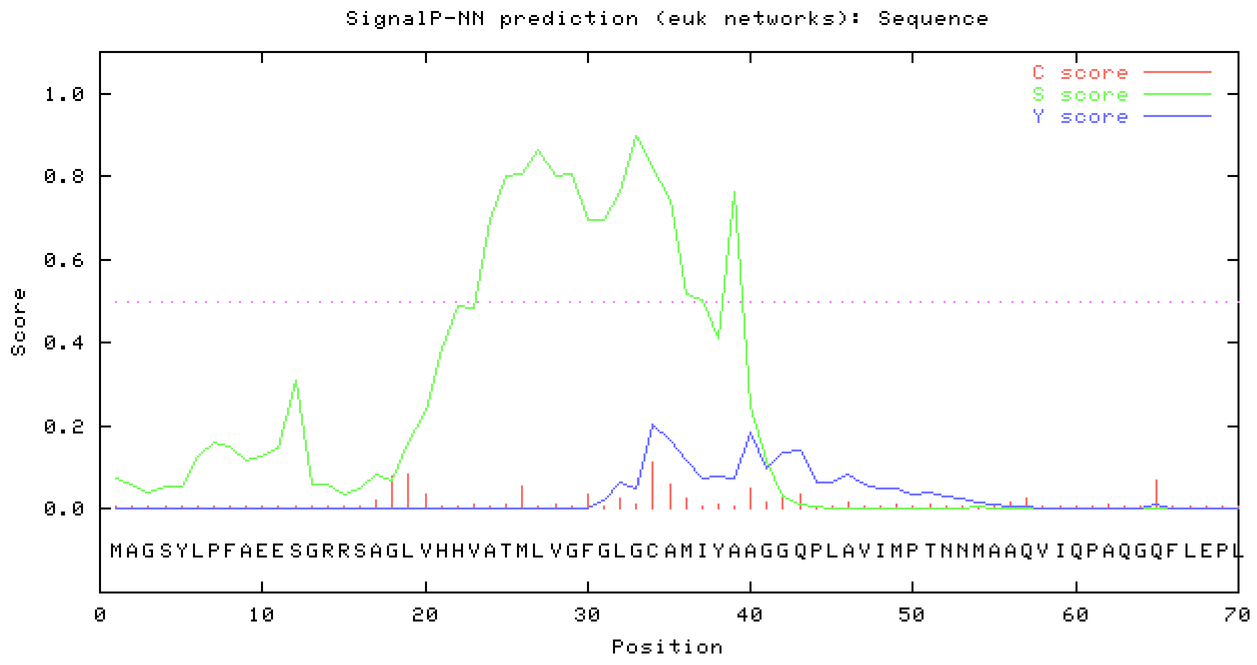

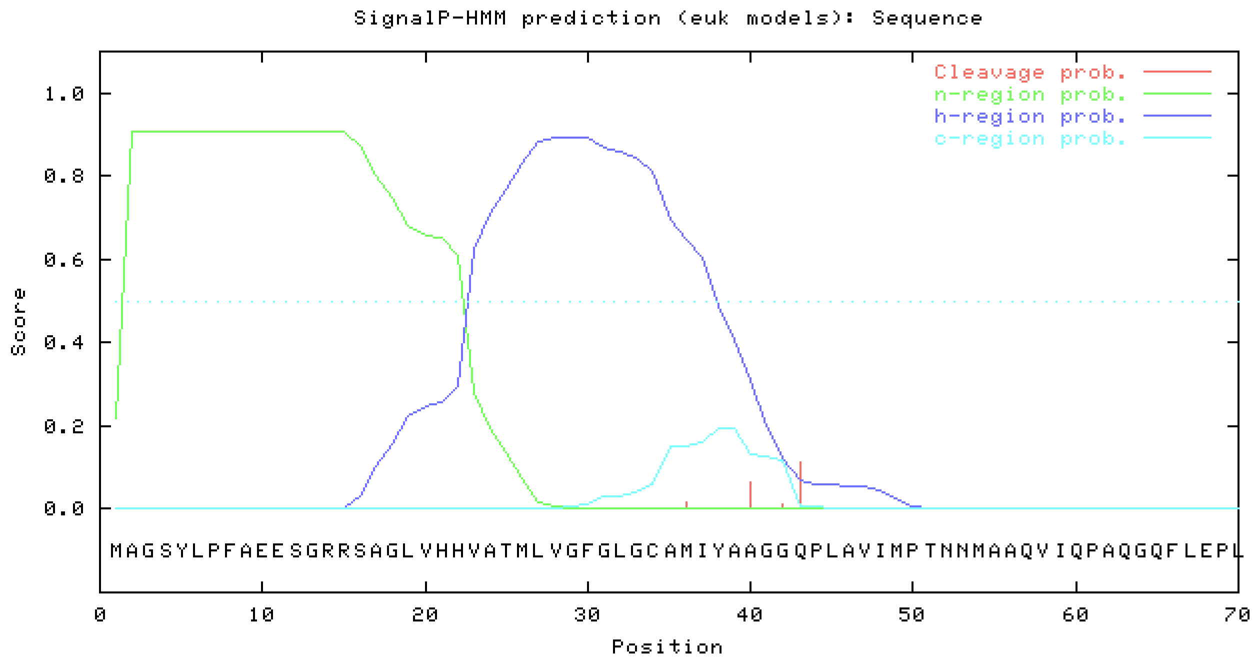

After N-21aa cut

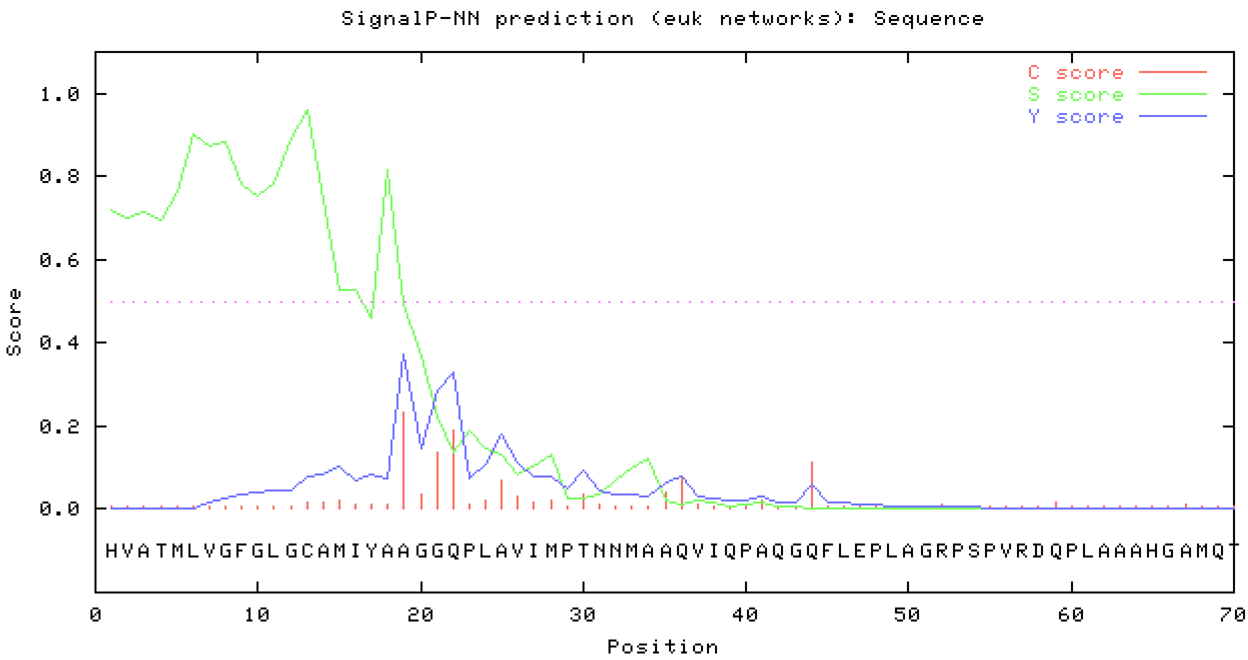

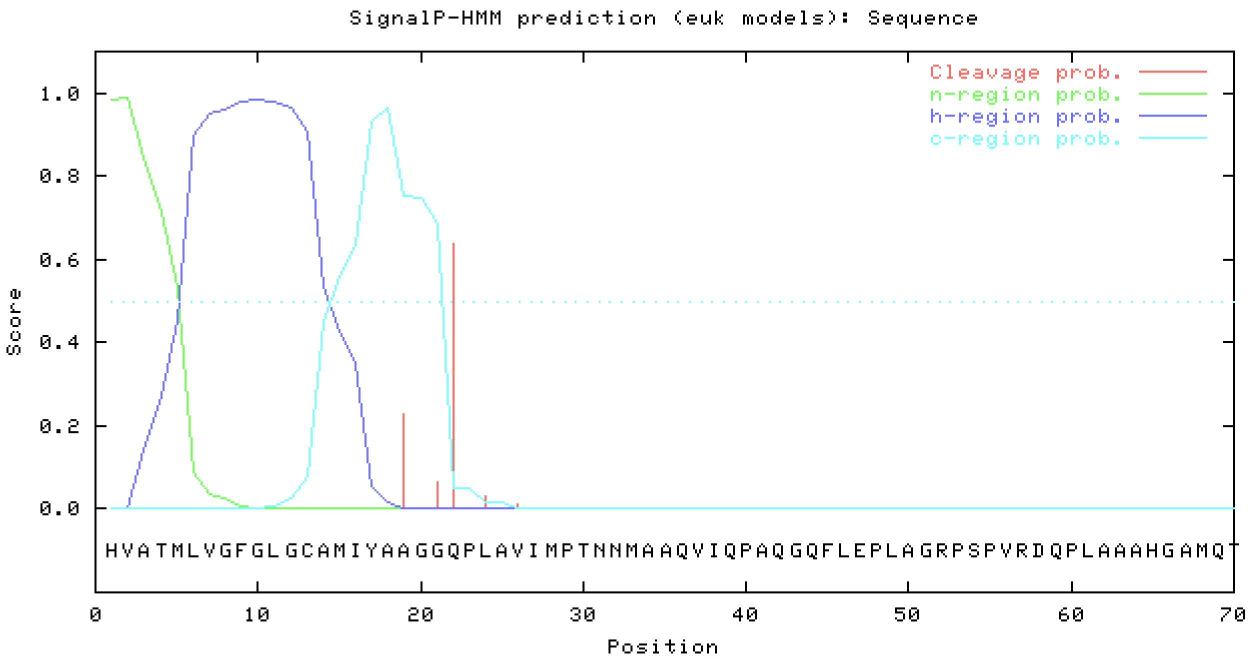

>rub_act_signal_transit_sequence

MPLPSSSQVDSMLREAIPSSIHLNSRKHAACFSAFTGLSFGGLLSFLLSASHAGQRLVADGSLIKMNSQFMKPKVARIGS
VSRKNPFVVHGGRWRGLDEDVSDDQQDITRGRHMVDSLFQGQQGLGGTHNAVMSSGPNVHQKNFGDSDVMQD

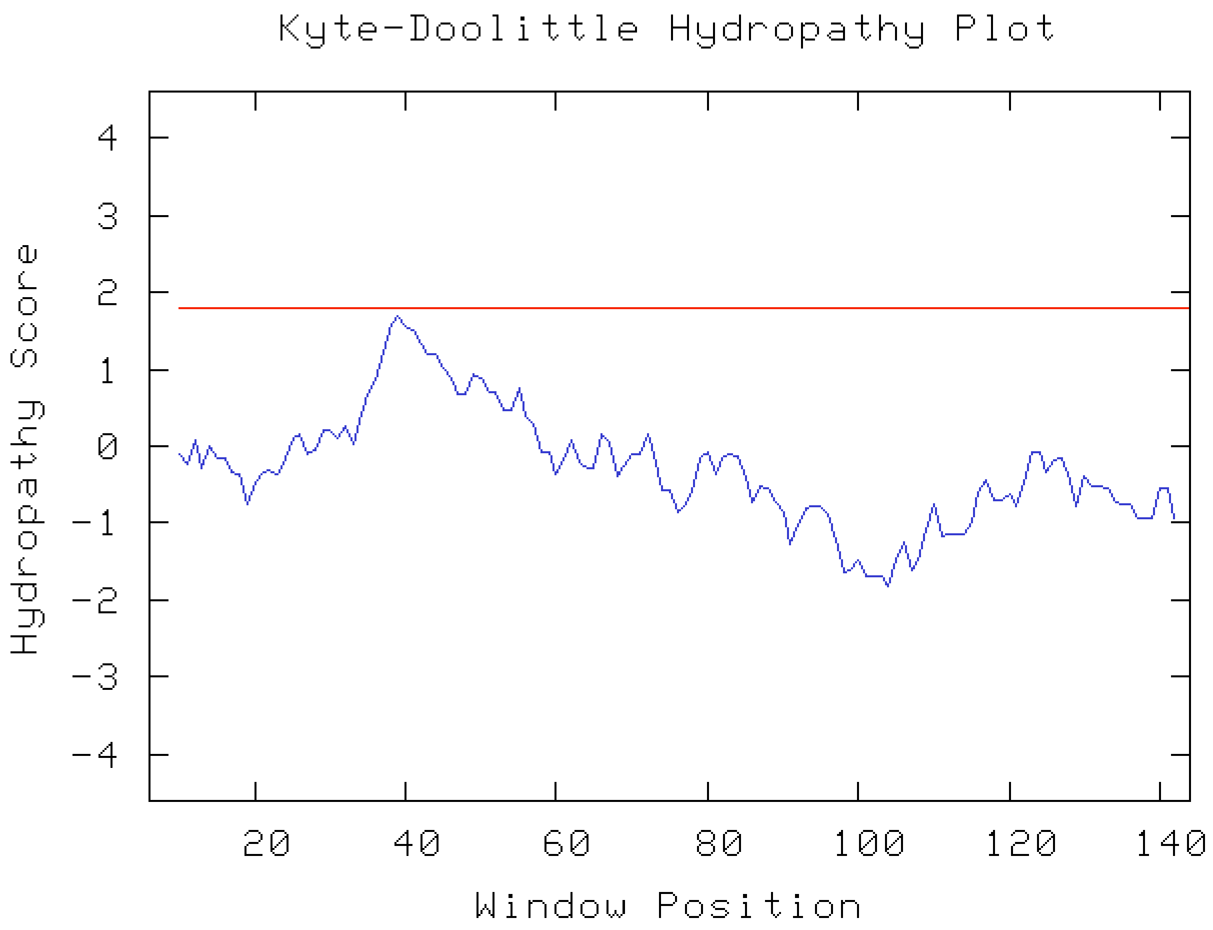

Kyte-Doolittle Hydropathy Plot
Hydropathy Score
4
3
2
1
0
-1
-2
-3
-4
20
40
60
80
100
120
140
Window Position

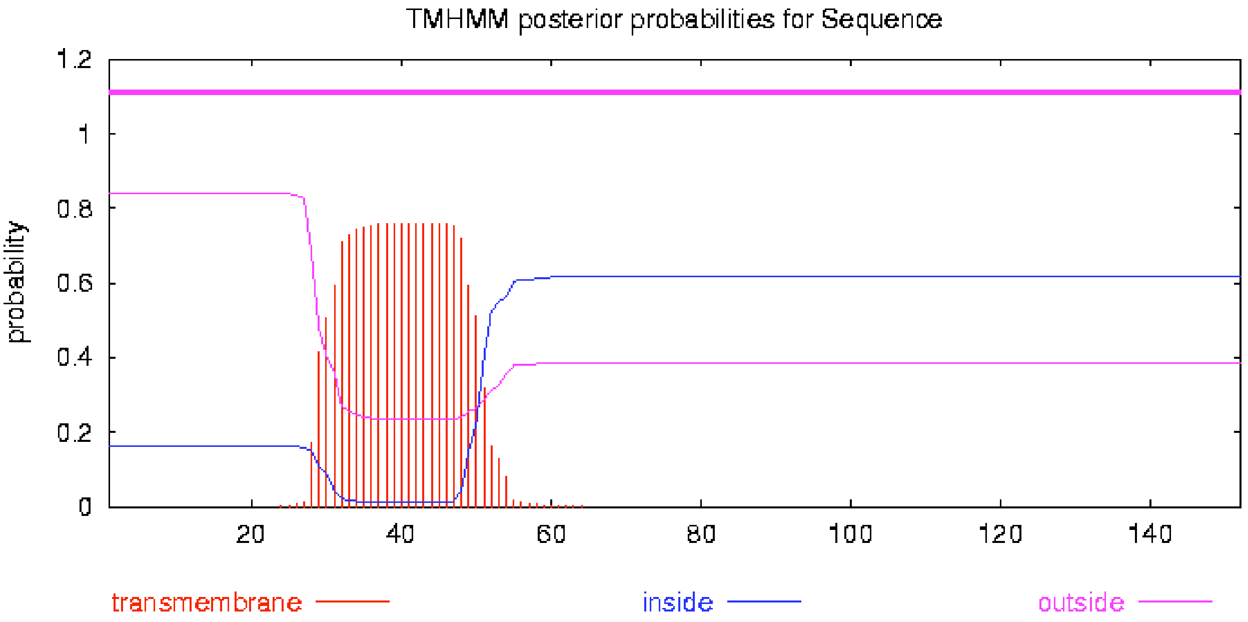

TMHMM posterior probabilities for Sequence
probability
1.2
1
0.8
0.6
0.4
0.2
0
20
40
60
80
100
120
140
transmembrane
inside
outside

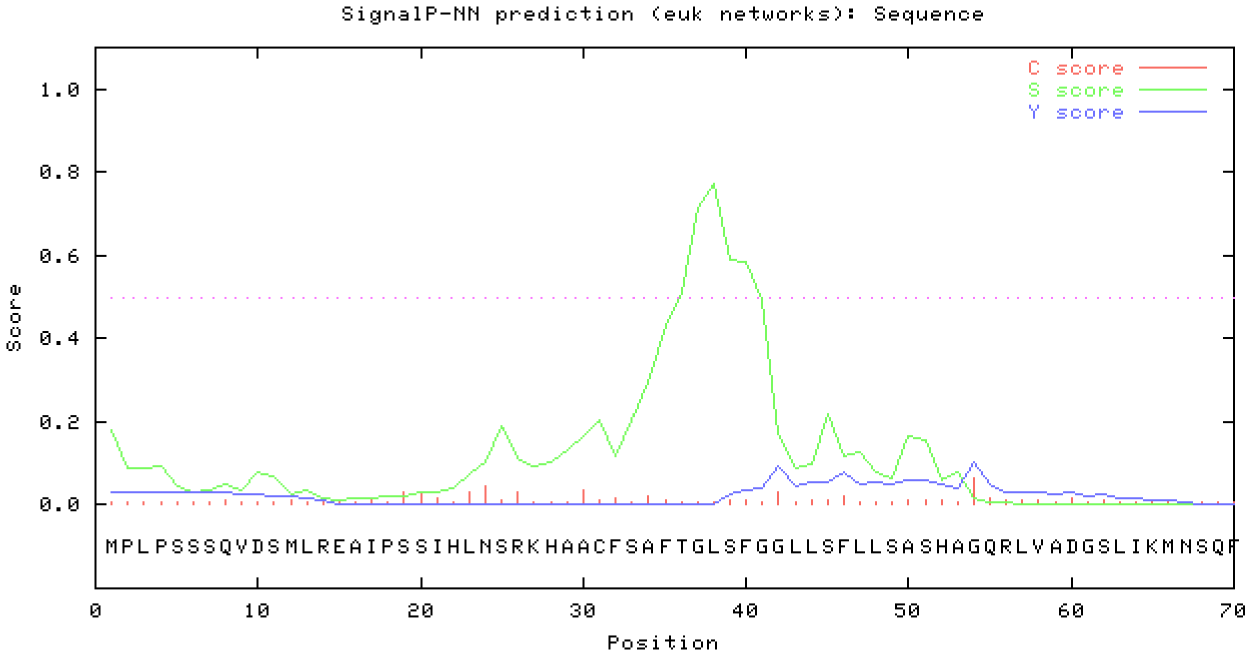

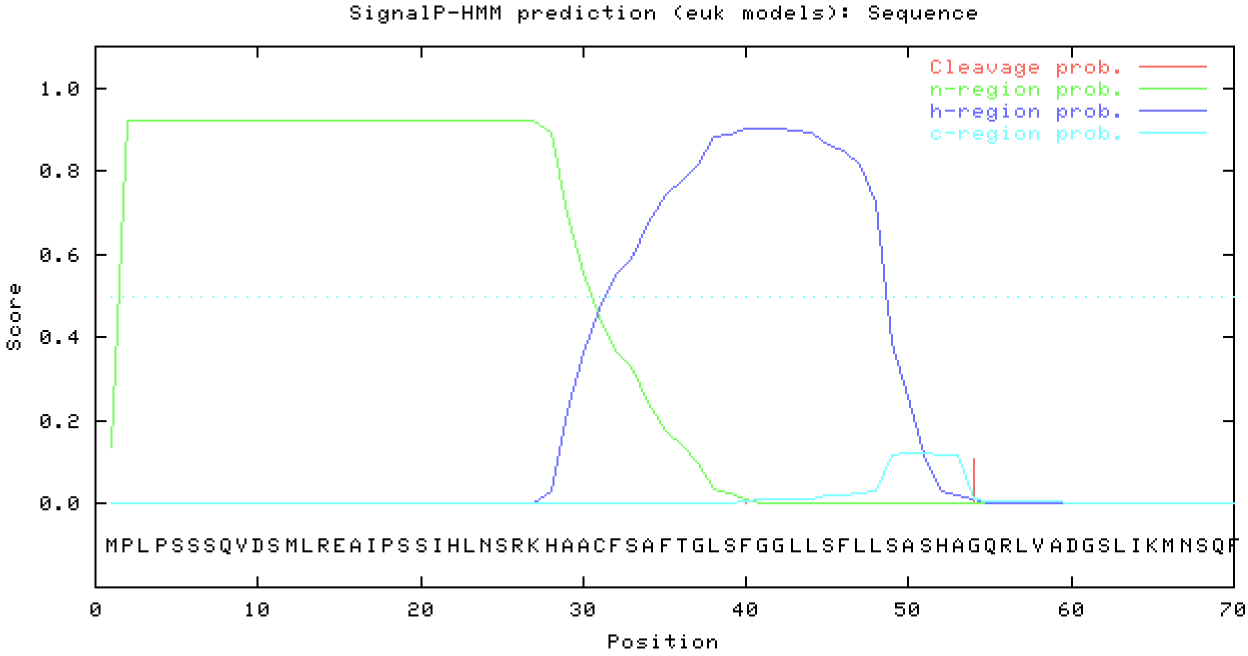

After N-30aa cut

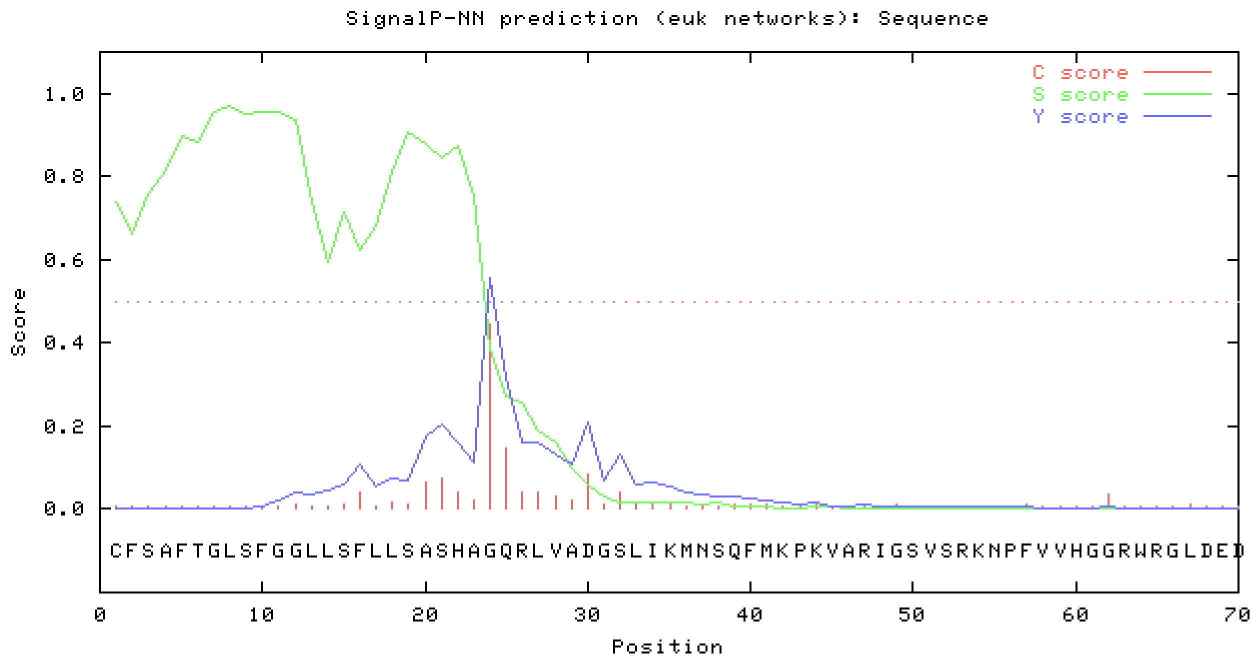

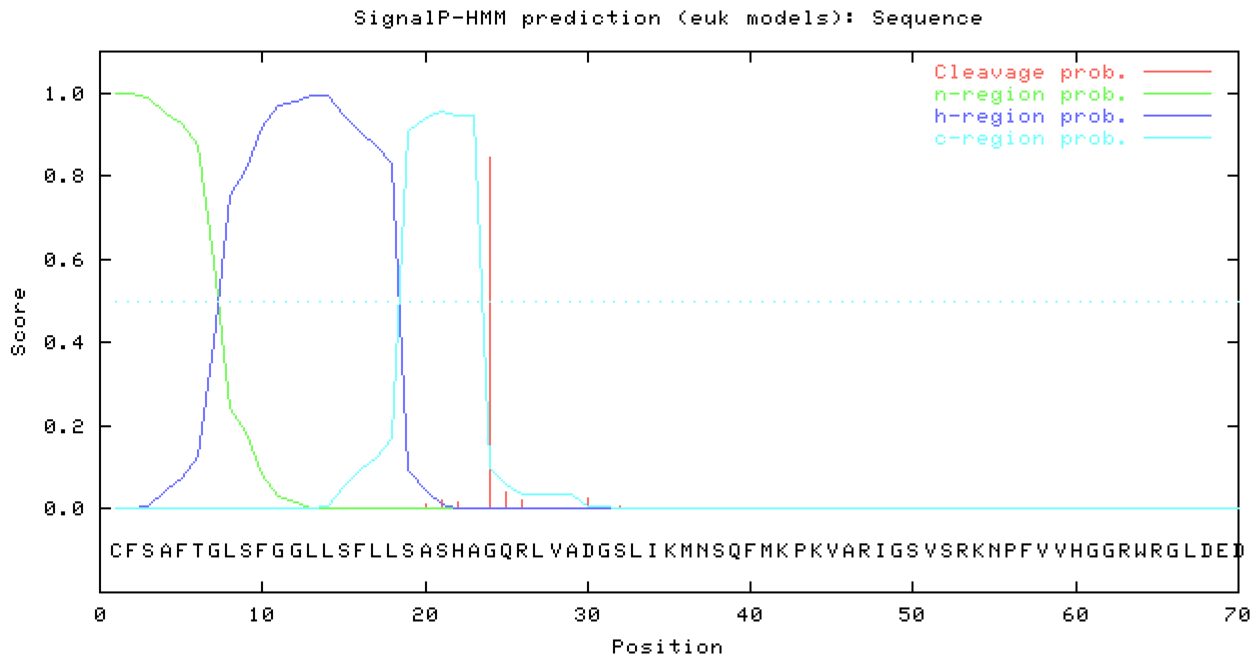

>sbp_signal_transit_sequence

MQGSMPETYVPLGEQMQEEAGLKRARHGRKIVAGLSMLIGVGLLMCLNSEASVPAETQEPAIATAWHGAMSRARSFIRPA
ARAKIHDVPHGKCMAPKGYRHSMHRGAAVGGKANFMKAEI

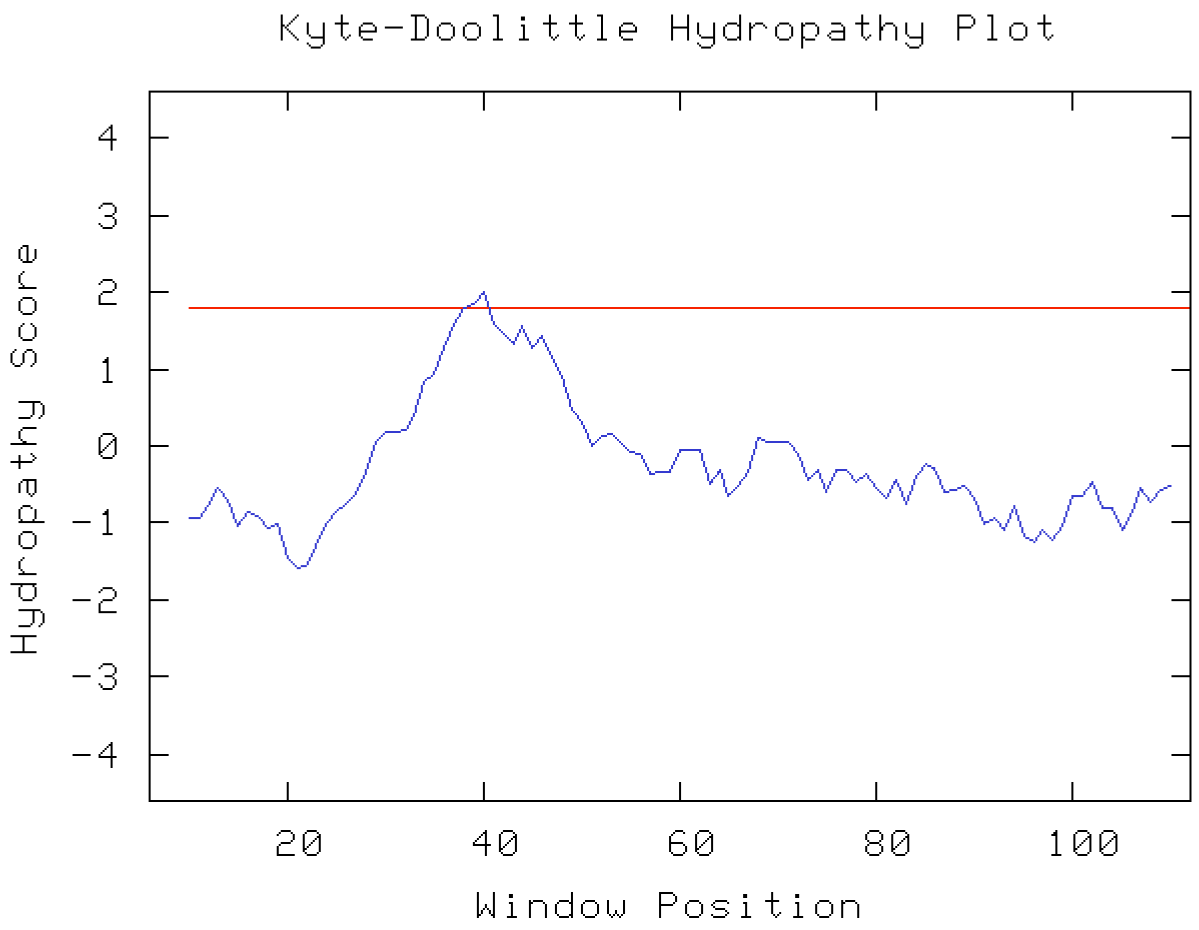

Kyte-Doolittle Hydropathy Plot
Hydropathy Score
4
3
2
1
0
-1
-2
-3
-4
20
40
60
80
100
Window Position

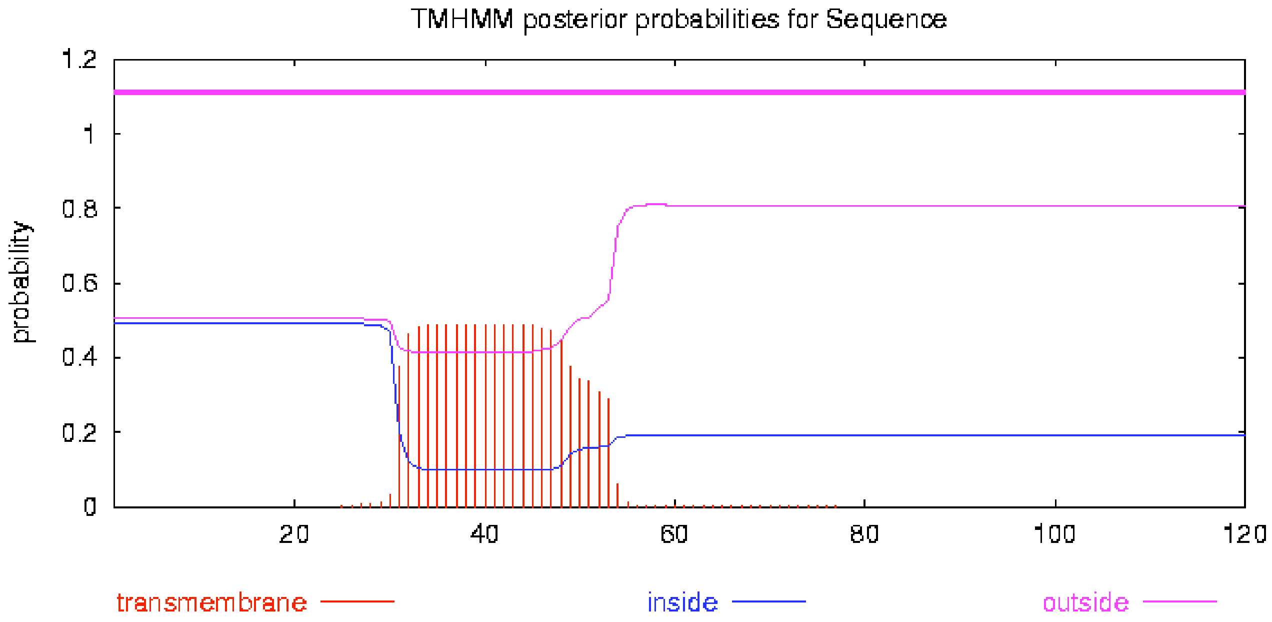

TMHMM posterior probabilities for Sequence
probability
1.2
1
0.8
0.6
0.4
0.2
0
20
40
60
80
100
120
transmembrane
inside
outside

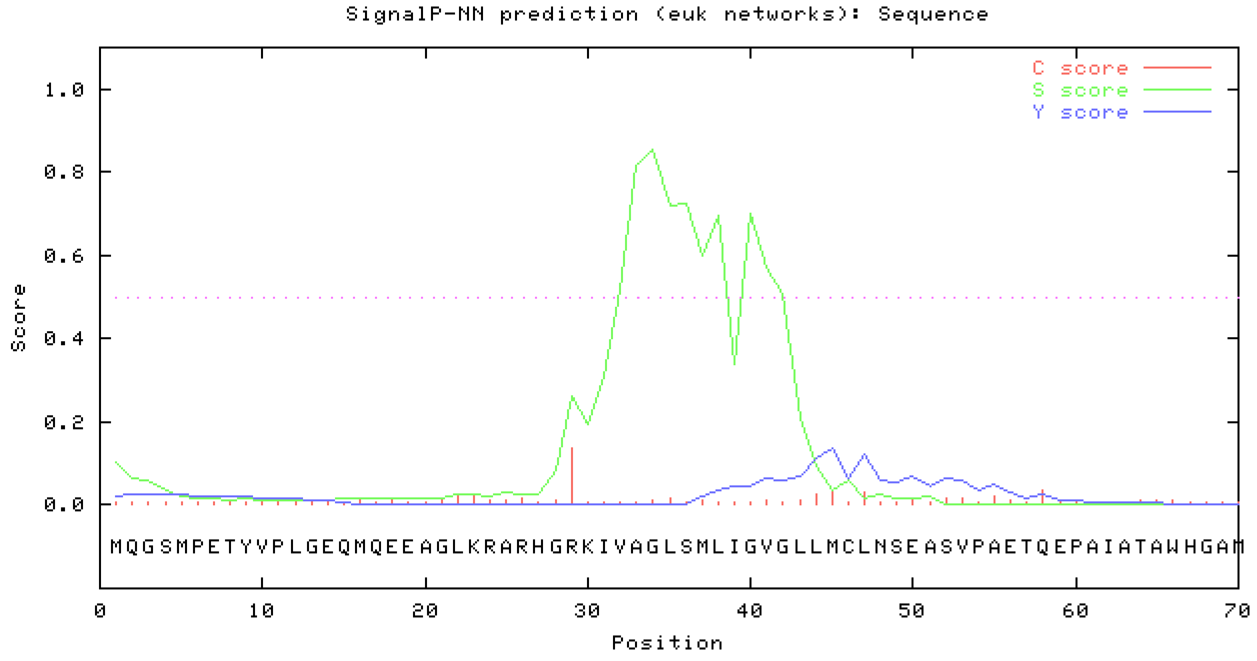

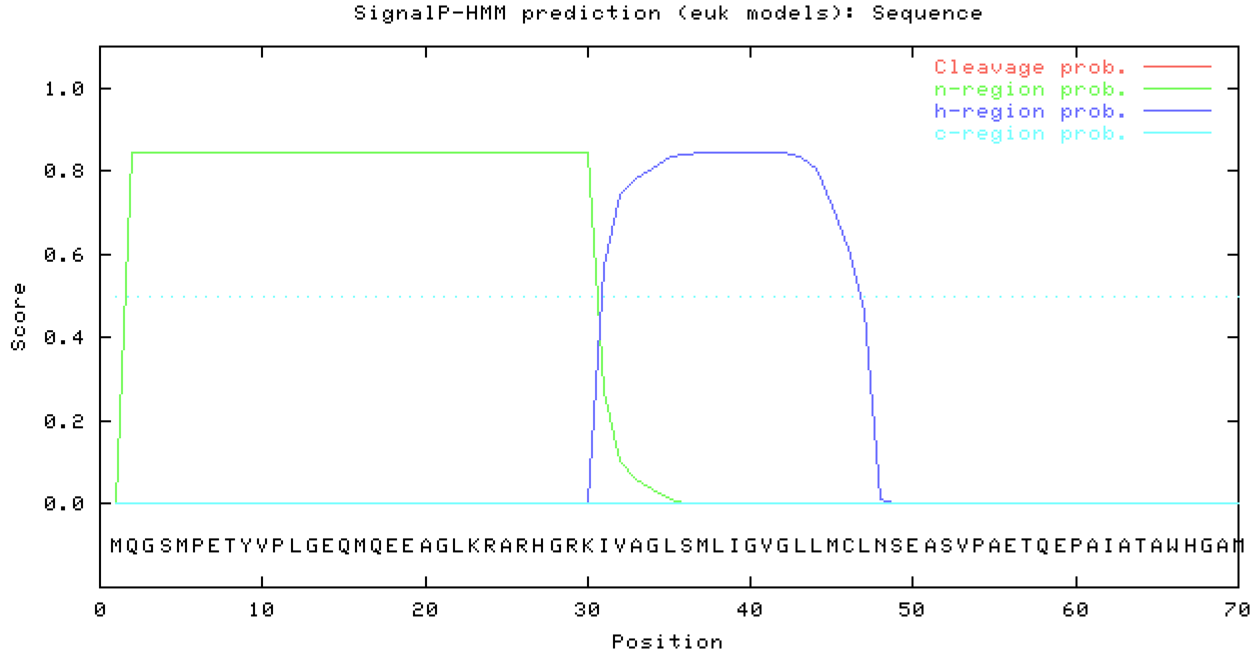

After N-30aa cut

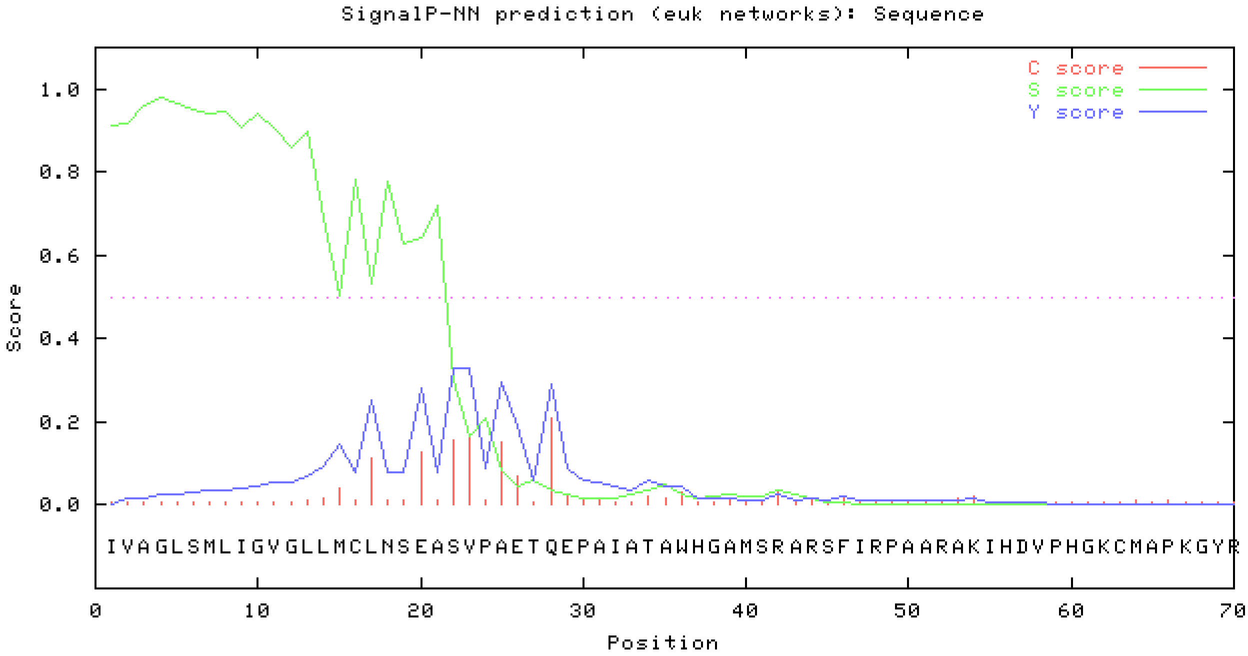

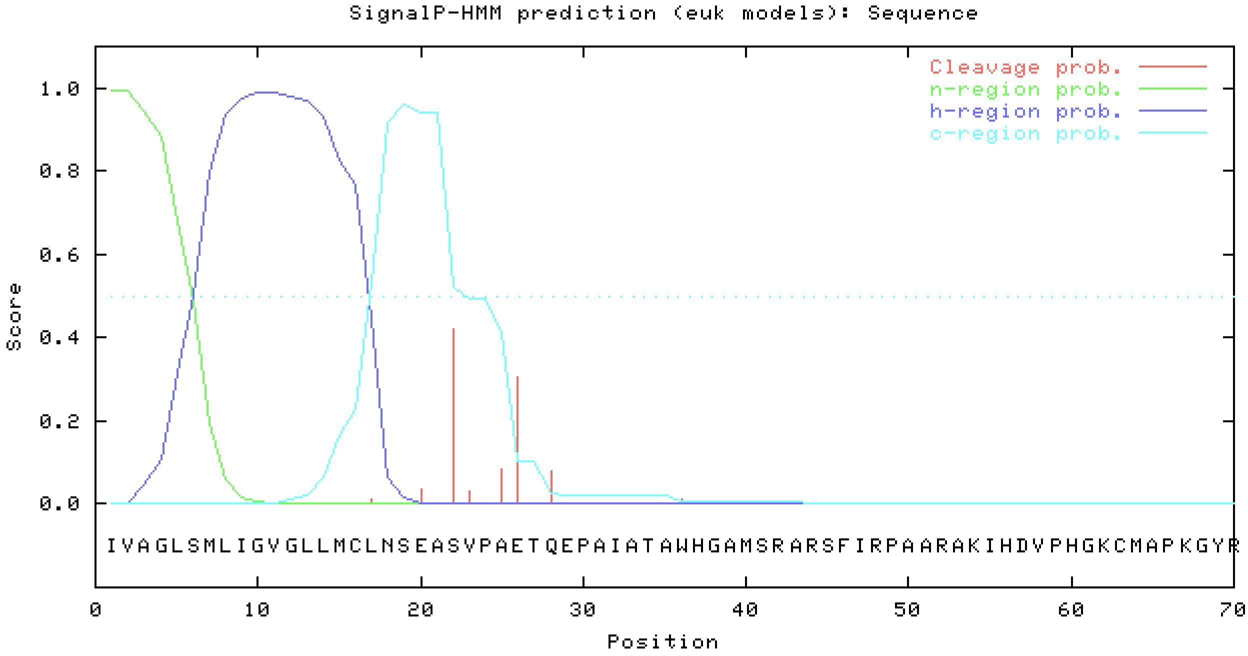

>transketolase_signal_transit_sequence

MCSIAAAMFNPHSALTEPLSDEAAPHSVRFGTKSLAFLLLGLGFAAMLSLPHGDLAANDSDITMALRHTQPARARFWGST

QSARARHMAYSVGSAGVADKVSTLLKENFPEAKDLNVKIHGGAADSTHFSVDLQFQSEAAPLEPALAAS

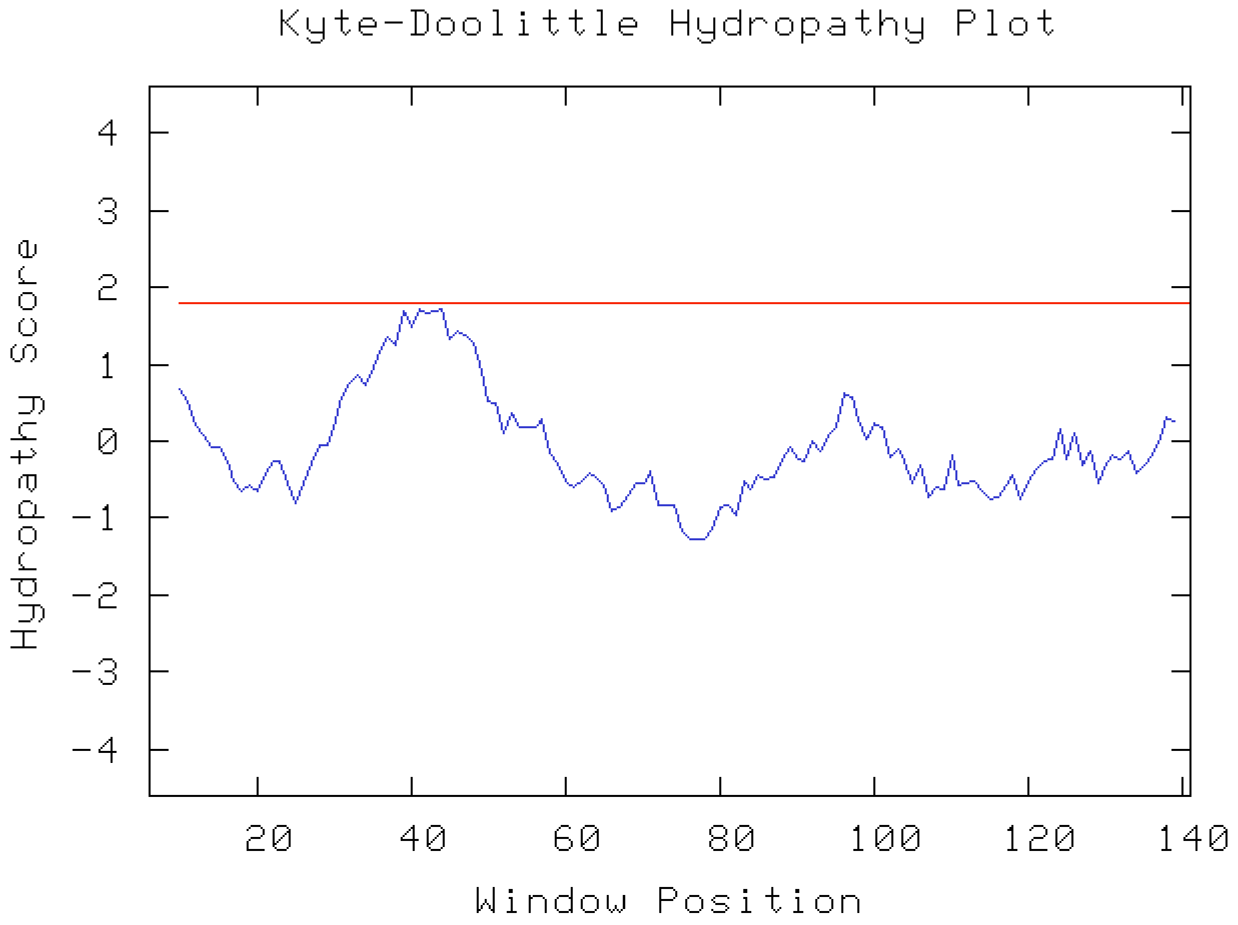
Kyte-Doolittle Hydropathy Plot
Hydropathy Score
4
3
2
1
0
-1
-2
-3
-4
20
40
60
80
100
120
140
Window Position

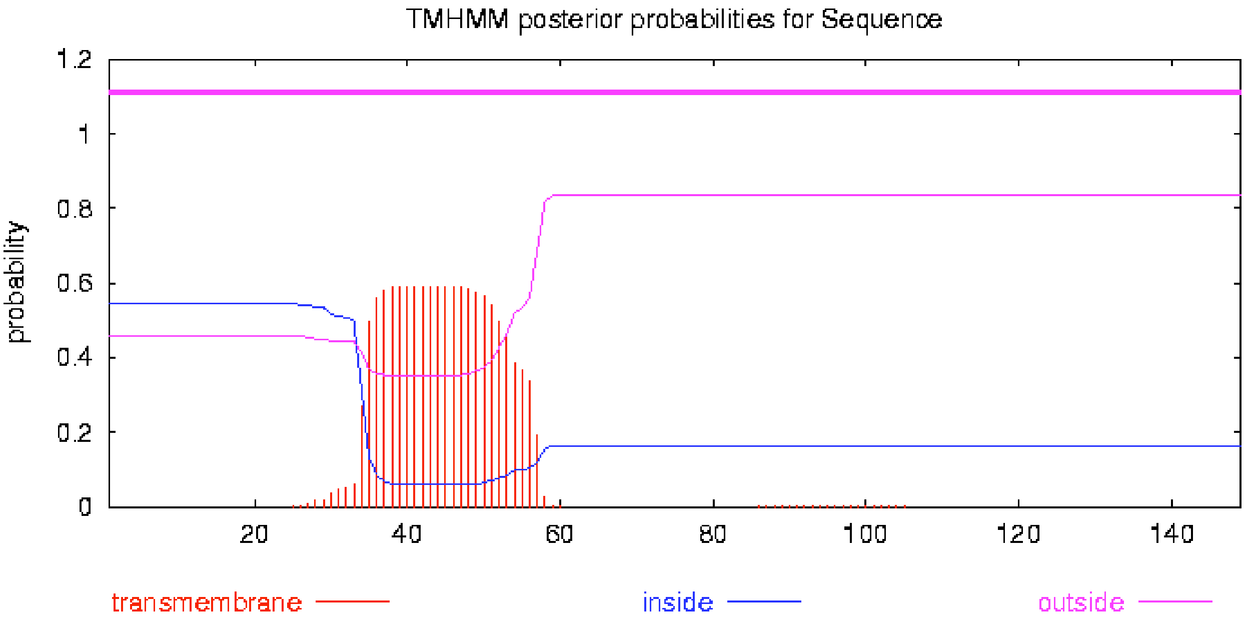
TMHMM posterior probabilities for Sequence
probability
1.2
1
0.8
0.6
0.4
0.2
0
20
40
60
80
100
120
140
transmembrane
inside
outside

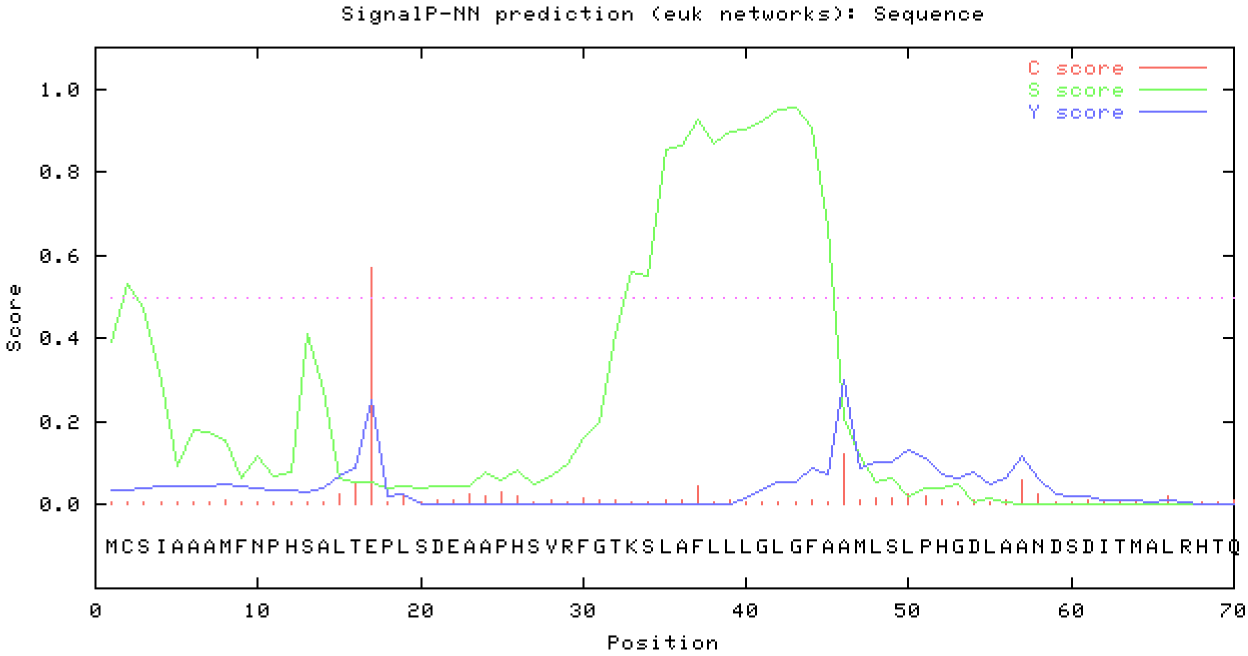

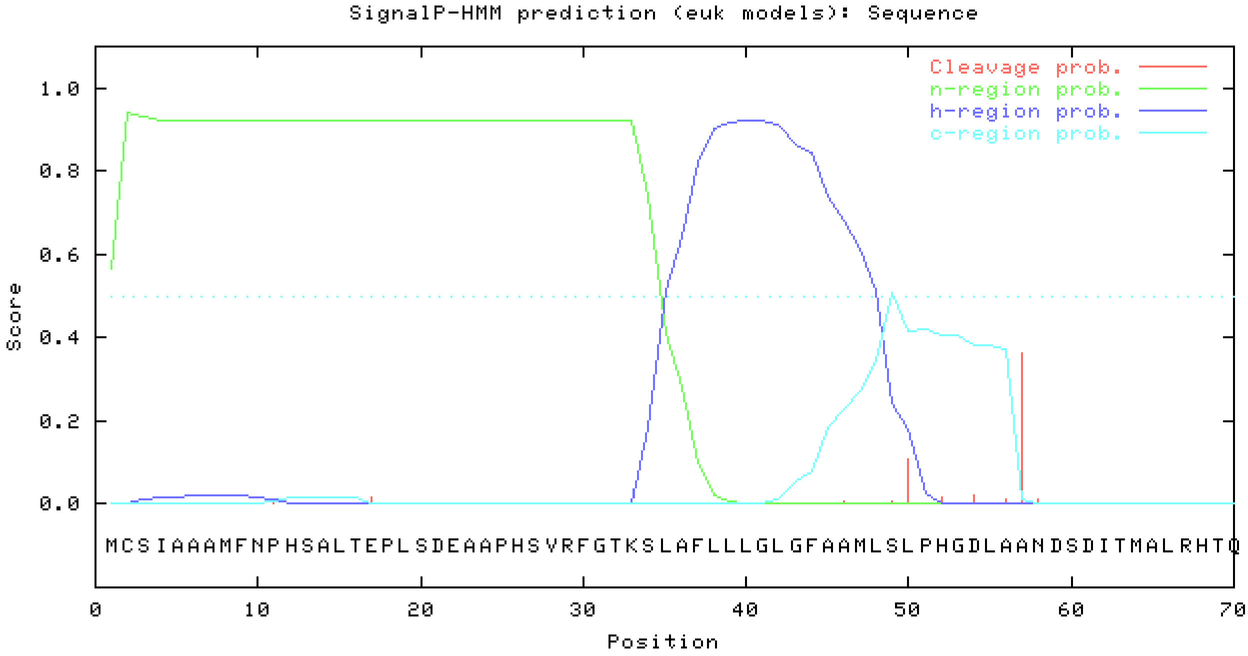

After N-35aa cut

SignalP-NN prediction (euk networks): Sequence

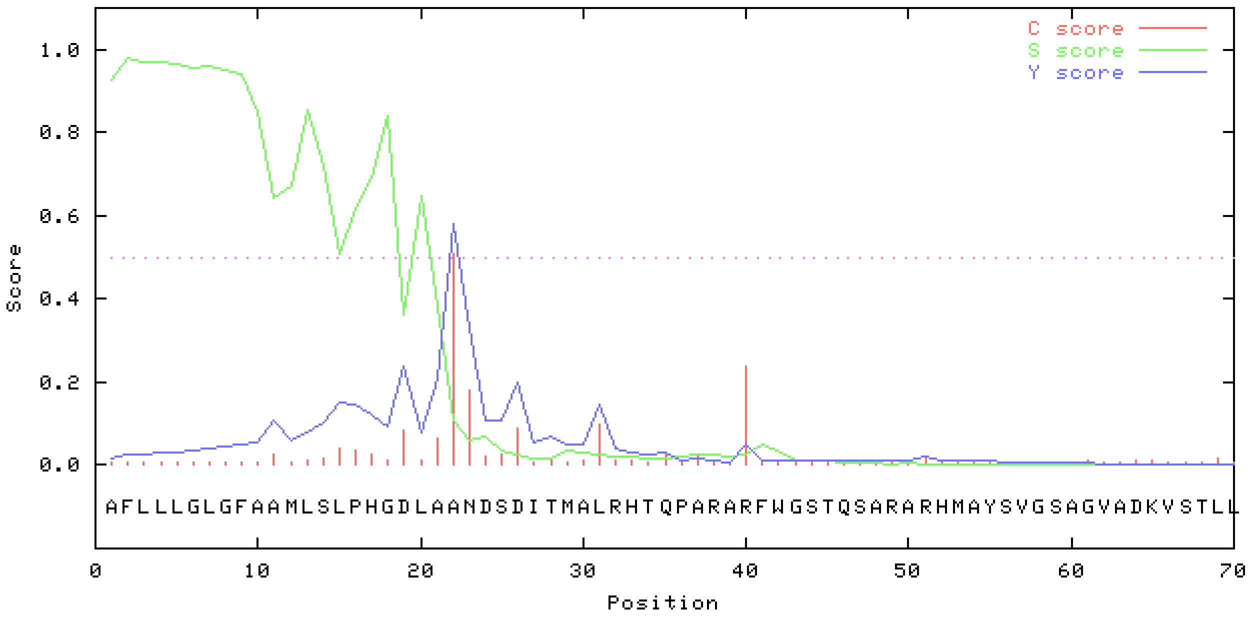

SignalP-HMM prediction (euk models): Sequence

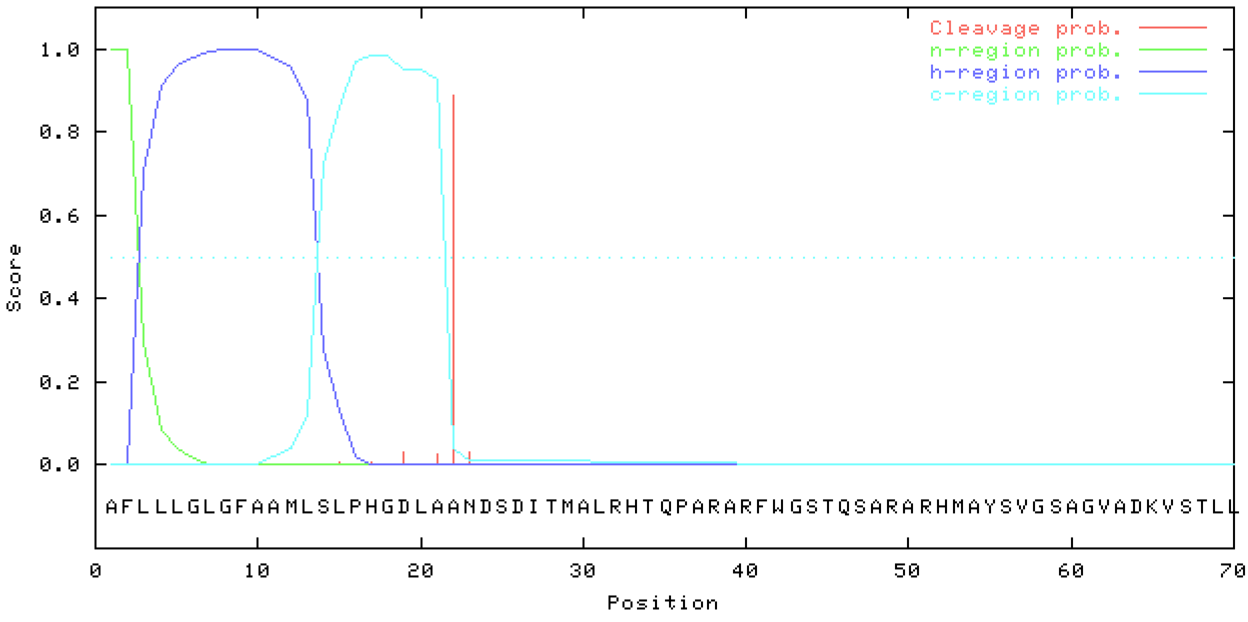

Supplement: Additional file 3 — Supplementary Figure S3: Kyte-Doolittle hydrophobicity plots of the N-terminal extensions (presequences). [file 1471-2148-10-191-S3.PDF]
