# Supplementary material for: A phylogenetic mosaic plastid proteome and unusual plastid-targeting signals in the green-colored dinoflagellate Lepidodinium chlorophorum
Source: BMC Evol Biol. 2010 Jun 21;10:191. doi: 10.1186/1471-2148-10-191 (PMC3055265; doi:10.1186/1471-2148-10-191)
Supplement: Additional file 6 — Supplementary Table S1: Accession numbers of sequences used in phylogenetic analyses. [file 1471-2148-10-191-S6.DOC]

PsbO

| *Alexandrium* | CF751886 |
| --- | --- |
| *Amphidinium* | CF065703 |
| *Arabidopsis* | Q9S841 |
| *Bigelowiella* | AAP79149_ |
| *Bruguiera* | Q9LRC4 |
| *Bryopsis* | BAF94219 |
| *Chara* | BAF94218 |
| *Chlamydomonas* | AB009092 |
| *Chlamydomonas* | P12853_1 |
| *Chlorarachnion* | BAF94212 |
| *Closterium* | BAF94220 |
| *Cyanothecesp* | Q9R6W6_ |
| *Emiliania* | CX772574 |
| *Euglena* | P46483 |
| *Eutreptiella* | BAF94211 |
| *Fritillaria* | O49079 |
| *Hafniomonas* | BAF94213 |
| *Heterocapsa* | Q8L879 |
| *Heterosigma* | AY130990 |
| *Isochrysis* | Q8L878 |
| *Karenia* | Q8L880 |
| *Lepidodinium* |  |
| *Lingulodinium* | BP742951 |
| *Micromonas* | EC846781 |
| *Nephroselmis* | BAF94217 |
| *Nicotiana* | Q40459 |
| *Nostoc* | P13907 |
| *Oedogonium* | BAF94214 |
| *Oryza* | Q943W1 |
| *Ostreococcus* | ES328969 |
| *Phaeodactylum* | Q84XB9 |
| *Pisum* | P14226 |
| *Prymnesium* | DV100611 |
| *Pyramimonas* | BAF94215 |
| *Scherffelia* | AJ919716 |
| *Solanum* | P23322 |
| *Spinacia* | P12359 |
| *Symbiodinium* | EH037912 |
| *Synechococcus* | P0A431 |
| *Tetraselmis* | BAF94216 |
| *Thalassiosira* | FC491926 |
| *Volvox* | Q9SBN6_1 |

**Rubisco activase**

| *Arabidopsis* | AAN18180 |
| --- | --- |
| *Chlamydomonas* | XP_001692244 |
| *Chlorococcum* | CAA71667 |
| *Deschampsia* | AAP83927 |
| *Gossipyum* | AAG61121 |
| *H_pluvialis* | DV203481 |
| *Ipomea* | ABX84141 |
| *Lepidodinium* |  |
| *Nicotiana* | Q40460 |
| *Oryza* | NP 001068555 |
| *Ostreococcus* | CAL51667 |
| *Ostreococcus* | XP 001417509 |
| *Phaseolus* | AAC12868 |
| *Physcomitrella* | XP_001778769 |
| *Physcomitrella* | XP 001752592 |
| *Physcomitrella* | XP 001776035 |
| *Triticum* | AAF71272 |
| *Vitis* | CAN83893 |
| *Volvox* | FD880922 |

PsbP

| *Arabidopsis* | Q42029 |
| --- | --- |
| *Brassica* | Q96334 |
| *Chlamydomonas* | ABA01138 |
| *Chlamydomonas* | P11471 |
| *Cucumis* | Q9SLQ8 |
| *Lepidodinium* |  |
| *Nicotiana* | P18212 |
| *Ostreococcus* | ABP00918 |
| *Sinapsis* | CAA35081 |
| *Solanum* | P93566 |
| *Synechococcus* | YP_730251 |
| *Thermosynechococcus* | BAC09627 |
| *Triticum* | CAA40669 |
| *Xerophyta* | AAN77240 |

PsbR

| *Arabidopsis* | NP 178025 |
| --- | --- |
| *Bigelowiella* | AAP79212 |
| *Brassica* | AAA74957 |
| *Brassica* | ABM54168 |
| *Chlamydomonas* | XP_001696588 |
| *Euglena* | EC671521 |
| *Hordeum* | Q40070 |
| *Lepidodinium* |  |
| *Mesostigma* | ABD37897 |
| *Nicotiana* | Q40519 |
| *Ostreococcus* | ABO96160 |
| *Physcomitrella* | XP_001762403 |
| *Prosopis _* | ABW35320 |
| *Solanum* | Q40163 |
| *Solanum* | P06183 |
| *Spinacia* | P10690_ |
| *Vitis* | CAN71762 |
| *Xerophyta* | AAN60205 |

PAP kinase

| *Arabidopsis* | NP_191914 |
| --- | --- |
| *Chlamydomonas* | XP_001689833 |
| *Dunaliella* | CX119726 |
| *Lepidodinium* |  |
| *Oryza_sativa* | NP_001053499 |
| *Ostreococcus* | CAL57996 |
| *Physcomitrella* | XP_001784533 |
| *Picea_sitchensis* | ABK21701 |
| *Vitis_vinifera* | CAO70588 |
| *Volvox* | FD920380 |

1-deoxy-D-xylulose-5 phosphate reductoisomerase

| *Artemisia* | AAD56391 |
| --- | --- |
| *Artemisia* | NP_001041780 |
| *Chlamydomonas* | XP_001693958 |
| *Chrysanthemum* | BAE79548 |
| *Elaeis* | AAS99589 |
| *Hanusia* | ABI96273 |
| *Hevea* | AAS94121 |
| *Lepidodinium* |  |
| *Mentha* | Q9XES0 |
| *Nicotiana* | ABH08964 |
| *Oryza* | EAZ10228 |
| *Ostreococcus* | ABO95578 |
| *Ostreococcus* | CAL51744 |
| *Paulinella* | ACB42486 |
| *Perkinsus* | BAG14386 |
| *Phaeodactylum* | ABI96274 |
| *Picrorhiza* | ABC74566 |
| *Prochlorococcus* |  |
| *Prymnesium* | ABI96271 |
| *Pyrocystis* | ABI96272 |
| *Rauvolfia* | AAY87151 |
| *Stevia* | CAD22156 |
| *Synechococcus* | ZP_01470404 |

Ferredoxin NADP oxireductase

| *Alexandrium* | CF947060 |
| --- | --- |
| *Amphidinium* | CF067646 |
| *Arabidopsis* | NP_564355 |
| *Bigelowiella* | AAP79145_ |
| *Chlamydomonas* | XP001697352 |
| *Heterocapsa* | AAW79314 |
| *Isochrysis* | AAW79315 |
| *Karenia* | ABF73016 |
| *Karlodinium* | ABA55546 |
| *Lepidodinium* |  |
| *Oryza* | ABF99221 |
| *Ostreococcus* | CCE9901 |
| *Phaeodactylum* | XP002184856 |
| *Prochlorococcus* | YP_291869 |
| *Prototheca* | AAV65380 |
| *Symbiodinium* | EG057925 |
| *Synechococcus* | YP_730216 |
| *Synechococcus* | NP_896844 |
| *Synecococcus* | P31973 |
| *Thalassiosira* | XP002295321 |
| *Volvox* | AAB40978 |

**4-disphosphocytidyl-2C-methyl-D erythriol kinase**

| *Arabidopsis* | NP_180261 |
| --- | --- |
| *Chlamydomonas* | XP_001694876 |
| *Clostridium* | ZP_02438267 |
| *delta_proteobacterium* | ZP_01289708 |
| *Emiliania* | EG357831 |
| *Ginkgo_biloba* | AAZ80385 |
| *Gloeobacter* | NP_923048 |
| *Gracilaria* | DV964176 |
| *Hevea* | BAF98293 |
| *Karlodinium* | EC160532 |
| *Lepidodinium* |  |
| *Leptospirillum* | UBA_EAY55988 |
| *Mesostigma* | ABO27194 |
| *Nicotiana* | ABO87658 |
| *Oryza* | EAY76173 |
| *Ostreococcus* | XP_001421612 |
| *Ostreococcus* | CAL58202 |
| *Perkinsus* | BAG14387 |
| *Physcomitrella* | XP_001772320 |
| *Plasmodium* | BQ739720 |
| *Prochlorococcus* | YP_001009319 |
| *Protochlamydia* | YP_008588 |
| *Salvia* | ABP96842 |
| *Solanum* | P93841 |
| *Synechococcus* | YP_381498 |
| *Thalassiosira* | FC536324 |
| *Thalassiosira* | FC536323 |
| *Verru_comicrobium* | ZP_02927615 |
| *Vitis* | CAO67301 |

Csp41

| *Alexandrium* | CF774717 |
| --- | --- |
| *Amphidinium* | CF064730 |
| *Anabaena* | YP_322618 |
| *Arabidopsis* | NP_191873 |
| *Aureococcus* | FC054206 |
| *Bigelowiella* | AAP79206 |
| *Chlamydomonas* | AAO22241 |
| *Emiliania* | GE147589 |
| *Isochrysis* | ABA55513 |
| *Karlodinium* | ABA55543 |
| *Laminaria* | CN467383 |
| *Lepidodinium* |  |
| *Nicotiana* | AAP87140 |
| *Nostoc_sp* | NP_488871 |
| *Oryza* | NP_001059177 |
| *Ostreococcus* | CAL57507 |
| *Phaeodactylum* | CU703912 |
| *Porphyra* | AU193288 |
| *Porphyra* | EG016776 |
| *Spinacia* | AAC49424 |
| *Synechocystis* | NP_440784 |
| *Trichodesmium* | YP_724075 |

Rbcs

| *Acetabularia* | P16132 |
| --- | --- |
| *Antithamnion* | AAM08169 |
| *Batophora* | P26985 |
| *Bigelowiella* | AAP79189 |
| *Chlamydomonas* | XP_001702409 |
| *Chloromonas* | AAD00448 |
| *Cyanidium* | BAA22829 |
| *Cylindrotheca* | P24683 |
| *Dunaliella* | AAS48504 |
| *Ectocarpus* | P24395 |
| *Emiliania* | YP277314 |
| *Euglena* | P16881 |
| *Gracilaria* | YP063671 |
| *Guillardia* | NP050706 |
| *Haematococcus* | ABB17556 |
| *Lemna* | P19308 |
| *Lepidodinium* |  |
| *Nannochloris* | BAD42333 |
| *Oryza* | NP001066606 |
| *Ostreococcus* | CAL58650 |
| *Phaeocystis* | BAF80672 |
| *Phaeodactylum* | YP874417 |
| *Pleurochrysis* | Q08052 |
| *Porphyra* | BAC84936 |
| *Porphyridium* | Q09125 |
| *Pteris* | CAA67061 |
| *Pteris* | CAA67061 |
| *Pylaiella* | P23652 |
| *Rhodomonas* | YP001293519 |
| *Saccharum* | Q41373 |
| *Secale* | CAA10497 |
| *Thalassiosira* | YP874497 |
| *Vaucheria* | YP002327543 |
| *Volvox* | AAO46871 |
| *Zantedeschia* | Q48550 |

**Phosphoribulokinase**

| *Arabidopsis* | NP_174486 |
| --- | --- |
| *Bigelowiella* | AAX13967 |
| *Chlamydomonas* | XP_001694038 |
| *Chondrus* | AAX13965 |
| *Cyanothece* | ZP_02939368 |
| *Euglena* | AAX13964 |
| *Guillardia* | AAX13960 |
| *Heterocapsa_* | AAW79321 |
| *Isochrysis_galbana* | AAW79322 |
| *Karlodinium* | ABA55537 |
| *Lepidodinium* |  |
| *Lingulodinium* | AAX13961 |
| *Mesembryanthemum* | P27774 |
| *Odontella* | CAA69902 |
| *Oryza_sativa* | NP_001047825 |
| *Ostreococcus* | CAL51688 |
| *Ostreococcus* | XP_001417260 |
| *Pavlova* | AAX13959 |
| *Physcomitrella* | XP_001756013 |
| *Pisum* | CAA72118 |
| *Prymnesium* | AAX13966 |
| *Pyrocystis* | AAX13963 |
| *Pyrocystis* | AAX13962 |
| *Synechococcus* | YP_479066 |
| *Trichodesmium* | YP_723190 |
| *Triticum* | CAA41020 |
| *Vaucheria* | AAK21910 |
| *Vitis* | CAN78901 |

**Fructose 1,6 bisphosphatase**

| *Arabidopsis* | CAA41154 |
| --- | --- |
| *Bigelowiella* | AAP79192 |
| *Brassica* | AAD12243 |
| *Chlamydomonas* | XP001690872 |
| *Cryptococcus* | XP566475 |
| *Euglena* | ABF68597 |
| *Galderia* | CAC82800 |
| *Guillardia* | ABF68599 |
| *Guillardia* | ABF68595 |
| *Isochrysis* | EC145974 |
| *Karenia* | ABF73027 |
| *Karlodinium* | EC155245 |
| *Lepidodinium* |  |
| *Lingulodinium* | ABF68596 |
| *Oryza* | NP001049664 |
| *Ostreococcus* | CAL52685 |
| *Phytophthora* | AAN31471 |
| *Phytophthora* | AAN31471 |
| *Pisum* | AAK59929 |
| *Populus* | XP002322946 |
| *Porphyra* | ABF68598 |
| *Porphyra* | BAG09539 |
| *Prymnesium* | DV098749 |
| *Solanum* | AAD25541 |
| *Spinacia* | P14766 |
| *Tetrahymena* | XP001007592 |
| *Vitis* | CBI30453 |

**Transketolase**

| *Acaryochloris* | YP001519794 |
| --- | --- |
| *Arabidopsis* | NP567103 |
| *Aspergillus* | XP752720 |
| *Aspergillus* | XP001268905 |
| *Bigelowiella* | ABQ23349 |
| *Capsicum* | CAA75777 |
| *Chlamydomonas* | XP001701881 |
| *Citrobacter* | YP001455802 |
| *Clostridium* | YP001311708 |
| *Cyanophora* | CAB58135 |
| *Dictyostelium* | XP644368 |
| *Enterobacter* | YP001178054 |
| *Erwinia* | YP048970 |
| *Escherichia* | CAA48166 |
| *Euglena* | ABQ23342 |
| *Euglena* | ABQ23343 |
| *Geobacillus* | YP001125304 |
| *Guillardia* | CAH25336 |
| *Haemophilus* | ZP01789898 |
| *Hetercapsa* | AAW79357 |
| *Isochrysis* | ABQ23345 |
| *Karlodinium* | ABP35605 |
| *Karlodinium* | ABQ23347 |
| *Karlodinium* | ABQ22346 |
| *Klebsiella* | YP001336984 |
| *Leishmania* | IR9J |
| *Lepidodinium* |  |
| *Monosiga* | XP001744412 |
| *Ostreococcus* | XP0014187845 |
| *Photorhabdus* | NP928282 |
| *Physarum* | A6YB01 |
| *Porphyra* | BAG09543 |
| *Salmonella* | ZP03214969 |
| *Serratia* | YP001479706 |
| *Shigella* | YP001881708 |
| *Sodalis* | YP455395 |
| *Solanum* | Q43848 |
| *Sorangium* | YP001614627 |
| *Trypanosoma* | ABU55008 |
| *Ustilago* | XP761114 |
| *Vibrio* | ZP04940612 |
| *Yersinia* | NP670609 |

**ATP synthase gamma**

| *Acaryochloris* | YP001515253 |
| --- | --- |
| *Anabaena* | YP323120 |
| *Arabidopsis* | NP567265 |
| *Bigelowiella* | AAP79136 |
| *Chlamydomonas* | XP001696335 |
| *Chlorobium* | YP912955 |
| *Cyanidioschyzon* | BAD36770 |
| *Guillardia* | CAH04622 |
| *Heterocapsa* | AAW79296 |
| *Isochrysis* | AAW79297 |
| *Karenia* | ABF73008 |
| *Karlodinium* | ABA55545 |
| *Lepidodinium* |  |
| *Moorella* | YP431204 |
| *Nicotiana* | P29790 |
| *Nodularia* | ZP01629865 |
| *Nostoc* | YP001868154 |
| *Odontella* | Q06908 |
| *Oryza* | NP001059768 |
| *Ostreococcus* | XP001418858 |
| *Pavlova* | ABA55584 |
| *Pelotomaculum* | YP001213363 |
| *Phaeodactylum* | AAO43198 |
| *Picea* | ABK26845 |
| *Prochlorococcus* | YP292173 |
| *Psychrobacter* | YP265306 |
| *Spinacea* | P05435 |
| *Synechococcus* | YP382481 |
| *Synechococcus* | NP896590 |
| *Synechococcus* | YP01468572 |
| *Thermosynechococcus* | NP681175 |
| *Trichodesmium* | YP721900 |
| *Vitis* | XP002518477 |

**Hcf136**

| *Acaryochloris* | YP001516953 |
| --- | --- |
| *Arabidopsis* | NP197703 |
| *Cyanophora* | NP043177 |
| *Guillardia* | XP001713238 |
| *Hemiselmis* | XO002181754 |
| *Lepidodinium* |  |
| *Microcystis* | CAO87924 |
| *Oryza* | EEX81352 |
| *Ostreococcus* | XP001420924 |
| *Pavlova* | ABA55570 |
| *Physcomitrella* | XP001756806 |
| *Synechococcus* | YP400195 |
| *Thermosynechococcus* | NP682485 |
| *Vitis* | XP002278958 |
| *Zea* | ABQ53629 |
